# Supplementary material for: Cluster randomised controlled trial to assess a tailored intervention to reduce antibiotic prescribing in rural China: study protocol
Source: BMJ Open. 2022 Jan 3;12(1):e048267. doi: 10.1136/bmjopen-2020-048267 (PMC8724711; doi:10.1136/bmjopen-2020-048267)
Supplement: Supplementary data [file bmjopen-2020-048267supp001.pdf]

Study Number:

Participated Clinic Identification Number for this trial:

CONSENT FORM

Title of Project: Design and evaluate a tailored intervention to modify antibiotic prescribing behaviour among health professionals and reduce antibiotic consumption: a cluster randomised controlled trial

Name of Researcher:

Please initial all boxes

1. I confirm that I have read and understand the information sheet dated [**DATE**] (version 1) for the above study. I have had the opportunity to consider the information, ask questions and have had these answered satisfactorily. ☐
2. I understand that my participation is voluntary and that I am free to withdraw at any time without giving any reason, without my medical care or legal rights being affected. ☐
3. I understand that any information I provide will be anonymised by removing all identifying details and will be kept strictly confidential and used only for research purposes. After the study, the anonymised information will be made publicly available for potential further research, but it will not be possible to identify me from the data. ☐
4. I understand that with my agreement, the interview may be audio-recorded but no one except the research team will hear this recording. I am aware that anonymised quotes from the interview may be included in reports of the study findings. ☐
5. I agree to take part in the above study. ☐

______________________	______________________	______________________
**Name of Participant**	**Date**	**Signature**

______________________	______________________	______________________
**Name of Person taking consent (if applicable)**	**Date**	**Signature**

______________________	______________________	______________________
**Name of Researcher**	**Date**	**Signature**

Consent form date of issue: **[DATE]**
Consent form version number: **[VERSION]**

Cong W, *et al. BMJ Open* 2022; 12:e048267. doi: 10.1136/bmjopen-2020-048267
