# Supplementary material for: Cluster randomised controlled trial to assess a tailored intervention to reduce antibiotic prescribing in rural China: study protocol
Source: BMJ Open. 2022 Jan 3;12(1):e048267. doi: 10.1136/bmjopen-2020-048267 (PMC8724711; doi:10.1136/bmjopen-2020-048267)
Supplement: Supplementary data [file bmjopen-2020-048267supp002.pdf]

Township health centre (THC) background information questionnaire
乡镇卫生院基本情况调查表

This questionnaire should be filled by the head of the THC
这张问卷请由此乡镇卫生院的负责医生填写。

名称：_______（市）_______县（区）________乡（镇）___________村
_______township (town),_______county, _______city

一、乡镇卫生院的基本情况
Basic information of THC

1、按照设置规划，本乡镇卫生院服务<br>1．1 覆盖地域面积为_______平方公里；<br>1．2 服务覆盖总人口__________人；<br>1．3 其中，常住人口________人；	1.The information about services of the township health centre<br>1.1 What is the service coverage area? ________square kilometers;<br>1.2 What is the total population covered by the service? ______;<br>1.3 What is the number of permanent residents covered by the service? ______;
2、目前，本乡镇卫生院所服务的行政村，实际共有其他村级医疗机构______个，并请说明医疗机构名称_______	2. At present, the administrative townships served by the THC actually have ______other township level medical institutions, *[please specify their names]* ___________
3、是否提供中医服务？（1）是 （2）否<br>3.1 包括哪些中医服务？<br>□草药；□针灸；□推拿；□其他______	3. Is TCM service provided here?<br>(1) Yes; (2) No<br>3.1 What TCM services are included?<br>□ herbal; □acupuncture; □ massage; □ other *[please specify]* __________
4、本乡镇卫生院能提供的医学检查有哪些？<br>□ 血液检查<br>□ X 射线<br>□ CT<br>□ B 超<br>□ 心电图检查<br>□ 没有<br>□ 其他检查手段，请描述______	4. What kinds of RTI related tests can this THC provide?<br>□Blood test<br>□X-Ray<br>□CT<br>□B Ultrasound<br>□ECG<br>□None<br>Other (Please Specify) _______
5、本乡镇卫生院有医生数量_________个.	5. The THC has_____ outpatient clinics; these clinics have _____doctors in total.

二、乡镇卫生院的医疗服务情况

Medical services in village clinics

2.过去一年接诊多少门诊病人？__________<br>2．1 其中，呼吸道感染的病人多少人？___	2. How many outpatients are there in the past year?<br>2.1 How many patients with respiratory infection?
