# Supplementary material for: Cluster randomised controlled trial to assess a tailored intervention to reduce antibiotic prescribing in rural China: study protocol
Source: BMJ Open. 2022 Jan 3;12(1):e048267. doi: 10.1136/bmjopen-2020-048267 (PMC8724711; doi:10.1136/bmjopen-2020-048267)
Supplement: Supplementary data [file bmjopen-2020-048267supp003.pdf]

Health practitioner background information questionnaire

乡镇卫生院医生基本情况调查表

This questionnaire should be filled by all clinicians working in this township health centre.

全部在此乡镇卫生院工作的医生都需要填写此表。

Gender?<br>□Male<br>□Female	性别?<br>□男<br>□女
Date of birth? ___________<br>Age: _____years.	出生日期：_____年_____月_____日；<br>或年龄？______岁
Education?<br>□ middle school and below;<br>□ high school /polytechnic school / technical school;<br>□ junior college;<br>□ undergraduate;<br>□ postgraduate and above;<br>□ others, please specify_____	学历？<br>□初中及以下；<br>□高中/中专/技校；<br>□专科；<br>□大学本科；<br>□研究生及以上；<br>□其他，请说明_____
How many years of medical training have you received? _______years；	你受到多少年的医学专业训练？<br>_______年；
What kind of medical training/learning do you have? What is your specialty?<br>□ Clinical medicine;<br>□ TCM;<br>□ Nursing;<br>□ Others, please specify_______	所学专业？<br>□临床医学；<br>□中医学；<br>□护理学；<br>□其他，请说明_______
How long have you worked as a physician? _______years；	您从事医生的工作年限？ _______年；
How long have you worked here? _______years；	你在这里工作多久了？ _______年；
Type of certificate you obtained:<br>□ licensed physician;<br>□ Certified assistant physician;<br>□ Registered nurse;<br>□ Village doctor;<br>□ No certificate	你获得证书类别：<br>□执业医师；<br>□执业助理医师；<br>□注册护士；<br>□乡村医生；<br>□无证书
Category of doctor practice:<br>□ Clinical;<br>□ Chinese medicine;<br>□ Public health<br>□ Others, please specify_______	医师执业类别：<br>□临床；<br>□中医；<br>□公共卫生<br>□其他，请说明_______
Do you have any further study experience? How many months in total?；<br>□ Yes (if yes, total months? _______months) ;<br>□ No;	你有没有进修的经历？<br>□有（如果有，共_____个月？）；<br>□无；
Have you ever participated in the training on the use of antibiotics? □Yes;□No;<br>If yes, how many times? _______times;<br>How many months in total? _______months.	有没有参加过关于抗菌药物使用的培训？<br>如果有，几次？<br>累计多少个月？
