# Supplementary material for: Cluster randomised controlled trial to assess a tailored intervention to reduce antibiotic prescribing in rural China: study protocol
Source: BMJ Open. 2022 Jan 3;12(1):e048267. doi: 10.1136/bmjopen-2020-048267 (PMC8724711; doi:10.1136/bmjopen-2020-048267)
Supplement: Supplementary data [file bmjopen-2020-048267supp004.pdf]

Study Number:

Patient Identification Number for this trial:

CONSENT FORM

Title of Study: Appropriate use of antibiotics for RTI in primary care: a cluster randomised controlled trial

Name of Researcher: XXX (contact details)

	**Please initial all boxes**
I confirm that I have read information sheet /went through information sheet with the researcher. I understand it and has had the opportunity to ask questions.	
I understand that my participation is voluntary and that I am free to withdraw at any time without giving any reason, without my medical care being affected.	
I understand that all the information is confidential. No name or personal information will be used when the results of this study is published.	
I agree to take part in the above study.	

Name of Participant **Date** **Signature**

Name of Person taking consent
(if applicable) **Date** **Signature**

Name of Researcher **Date** **Signature**

**Participant contact details (used for telephone questionnaire)**	
Phone number	
WeChat ID	

Consent form date of issue: **[DATE]**
Consent form version number: **[VERSION]**

Cong W, *et al. BMJ Open* 2022; 12:e048267. doi: 10.1136/bmjopen-2020-048267
