# Supplementary material for: Cluster randomised controlled trial to assess a tailored intervention to reduce antibiotic prescribing in rural China: study protocol
Source: BMJ Open. 2022 Jan 3;12(1):e048267. doi: 10.1136/bmjopen-2020-048267 (PMC8724711; doi:10.1136/bmjopen-2020-048267)
Supplement: Supplementary data [file bmjopen-2020-048267supp005.pdf]

Decision Support System & in clinic data collection items – for HP to use within consultation 诊断支持系统

Page 1: Eligibility criteria

第一页：入选条件

**Does patient have a suspected RTI?**	**病人是否疑似呼吸系统疾病?**
☐ Yes<br>☐ No – exclude from study	☐ 是<br>☐ 否一从本研究中排除
**Is today the first time the patient has sought treatment for this illness or a repeat visit?**	**今天是病人为此病的首次还是再次就诊?**
☐ First time consultation<br>☐ Repeat consultation – exclude from study	☐ 首次就诊<br>☐ 再次就诊--从本研究中排除
**Does patient need to be transferred due to COVID-19 suspected symptoms?**	**病人是否因疑似新冠需要被转入发热门诊?**
☐ Yes – exclude from study<br>☐ No	☐ 是 – 从本研究中排除<br>☐ 否

Page 2: Patient Symptoms

第二页：病人症状

**Consultation – Respiratory illness – Initial consultation – Illness history**	
**What symptoms are patient suffering from?** (tick all that apply)	**病人具体都有哪些症状?** (勾选所有符合的症状)
☐ Nasal congestion and running nose<br>☐ Cough<br>☐ Expectoration<br>☐ Sore throat<br>☐ Congestion/swelling of tonsils<br>☐ Fever<br>☐ Headache<br>☐ Earache	☐ 鼻塞，流鼻涕<br>☐ 咳嗽<br>☐ 咳痰<br>☐ 嗓子疼<br>☐ 扁桃体肿大<br>☐ 发烧<br>☐ 头疼<br>☐ 耳朵疼

☐ Muscular stiffness<br>☐ Chest congestion or shortness of breath<br>☐ Others	☐ 肌肉僵硬<br>☐ 胸闷或呼吸急促<br>☐ 其他_____________

Page 3 : Diagnosis, prognosis & patient reassurance

第三页：诊断，预后及病人安慰

Tick one	**What is the diagnosis?**	选一项打勾	**诊断是什么?**
	1. Acute upper respiratory tract infection including:<br>• Infectious cold / Acute rhinitis / upper respiratory tract catarrh<br>• Acute inflammation of the throat<br>• Acute viral pharyngitis<br>• Acute viral laryngitis		1.急性上呼吸道感染:<br>• 传染性感冒/急性鼻炎/上呼吸道粘膜炎<br>• 喉咙急性炎症<br>• 急性病毒性咽炎<br>• 急性病毒性喉炎
	2. Acute tonsil inflammation / Tonsillitis / Pharyngitis		2.急性化脓性扁桃体炎/ 扁桃体炎/咽炎
	3. Acute ear inflammation / Otitis Media		3.急性耳朵炎症/中耳炎
	4. Acute sinusitis		4.急性鼻窦炎
	5. Acute tracheal/ bronchial inflammation / Acute Tracheobronchitis / Bronchitis / Lower respiratory tract infection		5.急性气管/支气管炎症/急性气管支气管炎/支气管炎/下呼吸道感染
	6. Pneumonia		6.肺炎
	Other please state [free text box]		其他请说明

**Patient Reassurance**	**病人安慰**
• If you have diagnosed condition 1-5 reassure the patient that there is no sign of serious infection. Here are some things you can say to reassure patients:<br>○ There is no evidence of a serious infection<br>○ In your case the body can fight infections well on its own<br>○ You can take remedies to ease the symptoms<br>• If you have diagnosed Pneumonia, move straight on to Prognosis (next step) and Treatment (page 4).	• 如果您确诊病人所患疾病为 1-5 中之一，请确保该病人没有严重感染的迹象。以下是一些可以让患者放心的语句:<br>○ 没有严重感染的证据<br>○ 就你的情况而言，你的身体可以自己抵抗感染<br>○ 您可以使用一些疗法来缓解症状<br>• 如果您确诊病人所患疾病为肺炎，请直接进行预后（下一步）和治疗（第四页）

**Prognosis (Note only one option will apply)**			**病人预后 （只选一项）**		
Tick one	Most infections of this type are better by:		选一项打勾	此类感染一般情况下的好转时间是:	
	1. Acute upper respiratory tract infection	14 days		1.急性上呼吸道感染	14 天
	2. Acute tonsil inflammation / Tonsillitis / Pharyngitis	7-8 days		2. 急性化脓性扁桃体炎/ 扁桃体炎/咽炎	7-8 天
	3. Acute ear inflammation / Otitis Media	8 days		3. 急性耳朵炎症/中耳炎	8 天
	4. Acute sinusitis	14-21 days		4. 急性鼻窦炎	14-21 天
	5. Acute tracheal/ bronchial inflammation / Tracheobronchitis / Bronchitis / Lower respiratory tract infection	21 days		5. 急性气管/支气管炎症/急性气管支气管炎/支气管炎/下呼吸道感染	21 天
	6. Pneumonia	● 1 week: fever should have resolved<br>● 4 weeks: chest pain and sputum production should have substantially reduced<br>● 6 weeks: cough and breathlessness should have substantially reduced<br>● 3 months: most symptoms should have resolved but fatigue may still be present<br>● 6 months: most people will feel back to normal		6. 肺炎	• 1 周：发烧应该已经解决了<br>• 4 周：胸痛和痰的产生应该大幅减轻<br>• 6 周：咳嗽和呼吸困难应该大幅减轻<br>• 3 个月：大多数症状应该已经解决，但仍可能存在疲劳<br>• 6 个月：大多数人都会感觉恢复正常

Page 4: treatment recommendations (Note only one option will apply – select correct option for your patient)

第四页：治疗建议（只选一项， 请为您的病人选择正确的治疗建议）

**Treatment recommendation for each type of RTI (only one type of RTI below will appear based on the diagnosis choice in page 3)**	**每类呼吸道疾病的治疗建议（根据您第三页的诊断结果，以下六种疾病的治疗建议只有一种会出现）**
1. Acute upper respiratory tract infection<br>• Treatment should prioritize targeting symptoms and supporting the body to recover<br>• Antibiotics are usually not to be used and can be harmful, potentially causing side effects that can be severe, and increasing the risk of future serious antibiotic resistant infections in the patient, their family and the community<br>• See table below for symptomatic treatment options (there are just suggestions that come from Chinese National Guidelines)	1. 急性上呼吸道感染<br>• 治疗应优先针对症状并支持身体恢复<br>• 抗生素通常不被使用。其可能有害，并且可能导致严重的副作用并增加患者，其家人和社区未来严重抗生素耐药性感染的风险<br>• 有关症状治疗方案，请参见下表一（表中为国家指南的建议，供参考）
2. Acute tonsil inflammation / Tonsillitis / Pharyngitis<br>• Treatment should prioritize targeting symptoms and supporting the body to recover<br>• Antibiotics are usually not to be used and can be harmful, potentially causing side effects that can be severe, and increasing the risk of future serious antibiotic resistant infections in the patient, their family and the community<br>• See table 1 below for symptomatic treatment options<br>• Consider using antibiotics when:<br>○ There is suppuration of tonsils<br>○ Temperature of >38.5ºC	2. 急性扁桃体炎/扁桃体炎/咽炎<br>• 治疗应优先针对症状并支持身体恢复<br>• 抗生素通常不被使用。其可能有害，并且可能导致严重的副作用并增加患者、其家人和社区未来严重抗生素耐药性感染的风险<br>• 有关症状治疗方案，请参见表一<br>• 在下列情况下考虑使用抗生素<br>- 有扁桃体的化脓<br>- 体温> 38.5 ºC
3. Acute ear inflammation / Otitis Media<br>• Antibiotics are usually not to be used and can be harmful, potentially causing side effects that can be severe, and increasing the risk of future serious antibiotic resistant infections in the patient, their family and the community<br>• See table 1 below for symptomatic treatment options<br>• Consider using antibiotics if one or more of these symptoms are present (although note that the first three symptoms are common in viral ear infections):<br>○ loss of hearing<br>○ elevated temperature<br>○ progressive swelling and hyperemia in the tympanic membrane<br>○ perforation of the tympanic membrane accompanied by yellow exudate	3. 急性耳朵炎症/中耳炎<br>• 抗生素通常不被使用。其可能有害，并且可能导致严重的副作用并增加患者、其家人和社区未来严重抗生素耐药性感染的风险<br>• 有关症状治疗方案，请参见表一<br>• 如果存在以下一种或多种症状，请考虑使用抗生素（尽管研究已表明前三种症状在病毒性耳朵炎症中也是常见的）：<br>- 丧失听力<br>- 体温升高

	- 鼓膜中的进行性肿胀和充血<br>- 鼓膜穿孔伴有黄色渗出物
4. Acute sinusitis<br>• Antibiotics are usually not to be used and can be harmful, potentially causing side effects that can be severe, and increasing the risk of future serious antibiotic resistant infections in the patient, their family and the community<br>• See table 1 below for symptomatic treatment options<br>• Consider using antibiotics if one or more of these symptoms are present:<br>o Symptoms for more than 10 days<br>o Discoloured or purulent nasal discharge<br>o Severe localised unilateral pain (particularly pain over teeth and jaw)<br>o Fever<br>o Marked deterioration after an initial milder phase	4. 急性鼻窦炎<br>• 抗生素通常不被使用。其可能有害，并且可能导致严重的副作用并增加患者、其家人和社区未来严重抗生素耐药性感染的风险<br>• 有关症状治疗方案，请参见表一<br>• 如果存在以下一种或多种症状，请考虑使用抗生素：<br>- 症状超过 10 天<br>- 褪色或化脓性鼻涕<br>- 严重的局部单侧疼痛（尤其是牙齿和下颌疼痛）<br>- 发烧<br>- 初始缓和阶段后出现明显恶化
5. Acute tracheal/ bronchial inflammation / Tracheobronchitis / Bronchitis / Lower respiratory tract infection<br>• Symptomatic treatment should be considered priority.<br>• Antibiotics should not be routinely used and can be harmful, potentially causing side effects that can be severe, and increasing the risk of future serious antibiotic resistant infections in the patient, their family and the community.<br>• Consider anti-microbial treatment in the following cases:<br>o patients over 75 years of age with an elevated temperature;<br>o patients with heart failure;<br>o patients with insulin-dependent diabetes;<br>o patients with severe diseases of the nervous system.	5. 急性气管/支气管炎症/气管支气管炎/支气管炎/下呼吸道感染<br>• 首先考虑对症治疗<br>• 抗生素不应常规使用。其可能有害，并且可能导致严重的副作用并增加患者、其家人和社区未来严重抗生素耐药性感染的风险<br>• 在以下情况下考虑抗生素治疗：<br>- 患者 75 岁以上并伴体温升高<br>- 心力衰竭患者<br>- 胰岛素依赖型糖尿病患者<br>- 患有严重神经系统疾病的患者
6. Pneumonia<br>• The decision for outpatient or inpatient treatment should be based on the severity of illness, and anti-infection therapy should be administered as early as possible.<br>• Patients with mild symptoms and normal GI function should receive treatment with oral medication<br>• Patients with heavy symptoms should receive IV treatment, and change to oral medication once there is clear improvement in clinical appearance and ability to receive oral medication.<br>o Young adults, patients with no underlying conditions	6. 肺炎<br>• 门诊或住院治疗取决于疾病的严重程度，并应尽早进行抗感染治疗<br>• 症状轻微且胃肠功能正常的患者应接受口服药物治疗<br>• 症状较重的患者应接受静脉注射治疗，一旦临床表现和接受口服药物的能力明显改善应改为口服药物治疗<br>- 年轻人，没有潜在疾病的患者

▪ Penicillin; Amoxicillin; Doxycycline, minocycline; 1st or 2nd gen cephalosporins; Respiratory quinolones (moxifloxacin, levofloxacin, and Gemifloxacin)<br>○ The elderly or patients with underlying conditions<br>▪ Monotherapeutic usage of 2nd gen cephalosporins (cefuroxime axetil, cefprozil, cefaclor etc.) or combined usage with macrolides; Monotherapeutic usage of amoxicillin/clavulanic acid or ampicillin/sulbactam or combined usage with macrolides; Respiratory quinolones<br>Referral criteria (they are suggestions from Chinese National Guidelines; they may not all be useful or relevant to you):<br>• Combined with other underlying illnesses, such as chronic cardiac insufficiency (III-IV)<br>• Immunosuppression<br>• Initial treatment is failed but the vital signs are stable<br>• Local or systemic complications, such as pulmonary abscess, but vital signs are stable<br>• Older than 65 years of age with underlying illnesses, and assessed as having the infection risk of ESBL and other multi-resistant strains<br>• CAP identification is not clear and requires further diagnosis	◆ 青霉素；阿莫西林；强力霉素，米诺环素；第一代或第二代头孢菌素；呼吸喹诺酮类药物（莫西沙星，左氧氟沙星和吉米沙星）<br>- 老年人或有潜在疾病的患者<br>◆ 第二代头孢菌素（头孢呋辛酯，头孢丙烯，头孢克洛等）的单药治疗或与大环内酯类联合使用；阿莫西林/克拉维酸或氨苄青霉素/舒巴坦的单药治疗用途或与大环内酯联合使用；呼吸喹诺酮类<br><br>普通转诊（下列是国家指南的建议；以下建议不一定全部有用或者符合您当时的情况）：<br>• 合并基础疾病较多，如慢性心功能不全 (Ⅲ。Ⅳ级)、慢性肾脏疾病 3 ~ 5 期、肝硬化失代偿、 糖尿病急症<br>• 免疫抑制宿主发生 CAP<br>• 初始治疗失败，生命体征稳定<br>• 出现局部或全身并发症，如脓胸、肺脓肿， 生命体征稳定<br>• 年龄 / >65 岁有基础疾病患者，评估有超广 谱β-内酰胺酶 (ESBL)菌等耐多药感染风险<br>• CAP 诊断尚未明确，需要进一步鉴别诊断

Table 1 Symptomatic treatment options for RTI

表一 呼吸道感染对症治疗措施

**Symptoms**	**Symptomatic treatment options**	**症状**	**对症治疗措施**
temperature<br>headache<br>all-over muscle pain<br>pain in any area	Antipyretic analgesics including:<br>• Paracetamol<br>• Aspirin<br>• Ibuprofen*	发烧<br>头痛<br>全身肌肉疼痛<br>身体任何部位疼痛	解热镇痛药，包括：<br>• 对乙酰氨基酚/扑热息痛<br>• 阿司匹林<br>• 布洛芬*
blocked nose<br>hyperemia<br>edema in the nasal mucosa<br>sore throat	Compound decongestant and anti-allergenic medicine including:<br>• Pseudoephedrine<br>• 1% ephedrine nasal spray	鼻塞<br>充血<br>鼻粘膜水肿	复方抗充血剂和抗过敏药物，包括：<br>• 伪麻黄碱<br>• 1%麻黄素喷雾

		嗓子疼	
frequent sneezing<br>continual runny nose	Compound decongestant and anti-allergenic medicine including:<br>• chlorphenamine maleate<br>• loratadine<br>• diphenhydramine	频繁打喷嚏<br>持续流鼻涕	复方抗充血剂和抗过敏药物，包括：<br>• 马来酸氯苯那敏<br>• 氯雷他定<br>• 苯海拉明
Cough	Cough suppressants including:<br>• dextromethorphan hydrobromide<br>• codeine	咳嗽	止咳药，包括<br>• 右美沙芬<br>• 可待因
‘Inflammation’	TCM preparations may be effective<br>• yinqiao tablets<br>• shuanghuanglian<br>• antiviral granules<br>• yuxingcao (fish leaf)	‘炎症’	以下中成药或中药成分可能对你有效，包括：<br>• 银翘片<br>• 双黄连<br>• 抗病毒颗粒<br>• 鱼腥草

*Current Chinese national guidelines recommend using ibuprofen for symptoms of temperature, headache, all-over muscle pain and pain in any area; however, there is some research to show that the use of ibuprofen is unlikely to help the symptoms and is associated with their progression. Recent study has suggested that advising the use of ibuprofen may be unhelpful in managing RTIs.

*目前国家指南建议将布洛芬应用于发烧，头痛等呼吸道感染症状的对症治疗中。然而一些国外研究已经发现布洛芬对于其症状缓解没有帮助甚至会加重病情，已有研究提出布洛芬在呼吸系统感染治疗中是没有帮助的。

**Advice for all patients with diagnosis 1-5**	**对于所患疾病为 1-5 的病人的建议：**
The best way is to support your own body’s immune system to fight off the infection by, for example:<br>·Drink fluid (preferably water) at least 6 to 8 glasses of every day, particularly if you also have a fever;<br>·Keep warm and get plenty of rest;<br>·Eat a healthy, balanced diet containing plenty of fruit and vegetables.	最好的方法就是提高你自身的免疫力以预防感染，例如<br>• 每天至少喝 6 到 8 杯水（首选白开水），特别是发热的时候<br>• 保暖，多休息<br>• 健康均衡饮食，多吃水果蔬菜

**Things you can say to patients to help them understand that they do not need antibiotics**	**你可以给与病人以下建议，来帮助他们明白他们并不需要抗生素**
• Scientific research has found that antibiotics (e.g. Amoxicillin and Cephalosporin) do not help coughs, colds, sore throat or earache get better any faster;	• 科学研究发现抗生素（例如阿莫西林和头孢）并不能帮助咳嗽，感冒，嗓子疼，耳朵疼更快地好转
• Research has shown that in the past we took lots of antibiotics, such as Amoxicillin and Cephalosporin that we did not need	• 研究表明，在过去我们服用了很多我们不需要的抗生素，例如阿莫西林和头孢
• Taking antibiotics (e.g. Amoxicillin and Cephalosporin) may weaken your own body's methods for keeping you healthy, by killing the good bacteria in your gut.	• 服用抗生素（例如阿莫西林和头孢）会杀死肠道中的有益细菌，从而削弱身体保持健康的能力
• Taking antibiotics (e.g. Amoxicillin and Cephalosporin) for common infections now means that in the future antibiotics will work less well or not at all for you and your family	• 现在对常见的感染就使用抗生素（例如阿莫西林和头孢），意味着在未来，抗生素对你和你的家人的作用会更差或者根本不起作用
• Antibiotics can cause side effects (such as diarrhoea, rashes, feeling sick), which in rare cases can be very severe.	• 抗生素可以引起副作用(如腹泻、皮疹、恶心等)，在极少数情况下可能会非常严重

Page 5: Safety netting advice (when to come back to see the doctor)

第五页：复诊建议（何时再来看医生）

Finally, in the event of the following, please come back for treatment right away:	最后，如果出现以下现象请立即复诊:
. If your skin is very cold or has a strange colour, or you develop an unusual rash	• 如果你皮肤温度很低或者出现奇怪的颜色，或者出现异样皮疹
. If you feel confused have slurred speech, or become drowsy	• 出现头脑不清、言语不清、瞌睡
. If you have difficulty breathing including:<br>. hyperventilating<br>. your lips become purple<br>. skin between or above the ribs getting sucked or pulled in with every breath	• 出现呼吸困难，包括:<br>- 呼吸急促<br>- 嘴唇发紫<br>- 肋骨之间或之上的皮肤每次呼吸都会被吸入或拉入
. If you have a severe headache and are sick	• 出现严重头痛并感到恶心
. If you have a tight chest, chest pain,	• 出现胸闷、胸痛
. If you have difficulty swallowing or are drooling	• 出现吞咽困难或流口水
. If you cough up blood	• 出现咳血
. If you are feeling a lot worse	• 如果你感觉越来越差了
. If there is no improvement within (only one option will apply) – please note that most symptoms will be better before the listed length, but some can take this long and still get better without treatment<br>1. Acute upper respiratory tract infection – 14 days<br>2. Acute Tonsillitis / Pharyngitis – 8 days<br>3. Acute ear inflammation / Otitis Media – 8 days<br>4. Acute sinusitis – 21 days<br>5. Acute Tracheobronchitis – 21 days<br>6. Pneumonia – 1 week after treatment started	• 如果在正常恢复期内没有改善（每位患者只选一项）-请注意，大多数症状会提前好转，但是有些仍可能会持续到以下时间直至在没有治疗的情况下好转<br>1.急性上呼吸道感染 - 14 天<br>2.急性扁桃体炎/咽炎 - 8 天<br>3.急性耳朵炎症/中耳炎 - 8 天<br>4.急性鼻窦炎 - 21 天<br>5.急性气管支气管炎 - 21 天<br>6. 肺炎 - 已经接受治疗 1 周以后
