# Supplementary material for: Cluster randomised controlled trial to assess a tailored intervention to reduce antibiotic prescribing in rural China: study protocol
Source: BMJ Open. 2022 Jan 3;12(1):e048267. doi: 10.1136/bmjopen-2020-048267 (PMC8724711; doi:10.1136/bmjopen-2020-048267)
Supplement: Supplementary data [file bmjopen-2020-048267supp006.pdf]

Participant ID: ___________________

WP1 Clinical consultation doctor questionnaire

These questions must be answered by the **doctor** or by direct observation by the field worker

这部分问题要求**医生**回答或者通过调查员的观察完成 Consultation start time: __ h __ min (24-h clock)

开始接诊时间 _____________时_______分（24 小时制）

1. Whether practitioner has checked with patient’s symptoms? 医生是否询问症状

☐ Yes 是
☐ No 否

1.1 What are the patient’s symptoms? 病人当前有何症状?

☐ Blocked/ runny nose	☐ 鼻塞/流涕
☐ Blocked nose<br>☐ Runny nose (clear/watery discharge)<br>☐ Snotty nose (yellow/green discharge)	☐ 鼻子不通<br>☐ 流清水鼻涕<br>☐ 流脓鼻涕 (黄/绿色鼻涕)
☐ Coughing	☐ 咳嗽
☐ Dry cough<br>☐ Cough with white sputum<br>☐ Cough with yellow/green sputum	☐ 干咳<br>☐ 咳嗽带白色痰<br>☐ 咳嗽带黄/绿色脓痰
☐ Throat problems	☐ 嗓子不舒服
☐ Sore throat<br>☐ Hyperemia/ swelling in throat<br>☐ Hyperemia/ enlargement of tonsils<br>☐ Pus on the tonsils<br>☐ Swollen lymph glands	☐ 咽痛<br>☐ 咽喉部充血/水肿<br>☐ 扁桃体充血/肿大<br>☐ 扁桃体化脓<br>☐ 下颌淋巴结肿大
☐ Breathing	☐ 呼吸
☐ Short of breath<br>☐ Tight chest<br>☐ Difficulty breathing<br>☐ Must sit erect to breathe<br>☐ Wet crackling in lungs	☐ 气促<br>☐ 胸闷<br>☐ 呼吸不畅/困难<br>☐ 端坐呼吸<br>☐ 肺部湿罗音
☐ Ear symptoms	☐ 耳部症状
☐ Blocked ears<br>☐ Tinnitus<br>☐ Pus/ fluid secretion<br>☐ Earache<br>☐ Loss of hearing	☐ 耳闷<br>☐ 耳鸣<br>☐ 流脓/液<br>☐ 耳痛<br>☐ 听力下降
☐ Fever	☐ 发热

Participant ID: ________________

☐ Low 37.3-38 ºC<br>☐ Moderate 38.1-39 ºC<br>☐ High >39 ºC	☐ 低热 37.3-38 摄氏度<br>☐ 中等度热 38.1-39 摄氏度<br>☐ 高热 39 摄氏度以上
☐ Pain	☐ 疼痛
☐ Headache<br>☐ Aching all-over<br>☐ Chest pain	☐ 头痛<br>☐ 全身酸痛<br>☐ 胸痛

Any other symptoms? If yes please state ______________________

除了以上症状，你还有其他不舒服的症状么？ ____________________________

1.2 What kinds of tests were ordered by practitioners? (Please tick and/or write down) （医生给病人开具了哪些检查，请选择或具体填写）

☐Blood test （血液检查）

☐X-Ray （X 射线）

☐CT （CT）

☐B Ultrasound (超声波诊断，B 超)

☐ECG （心电图检查）

☐None （没有）

Other (Please Specify) （其他的，请描述） _______________________

1.3 What kinds of tests were carried out by patient? (Please tick and/or write down) （病人实际做了哪些检查，请选择或具体填写）

☐Blood test （血液检查）

☐X-Ray （X 射线）

☐CT （CT）

☐B Ultrasound (超声波诊断，B 超)

☐ECG （心电图检查）

☐None （没有）

Other (Please Specify) _（其他的，请描述） ______________________

1.4 Test results （检查结果）

Record practitioners’ statement about test results as told to the patient, in his/her own words. If no test result is stated, then write down 'not stated'. 如果医生有告知病人的检查结果，请原话记录医生的描述。如果检查结果没有告知，请填写”未告知”。

____

Participant ID: ___________________

Diagnosis & Treatment 诊断和治疗

2. Whether practitioner has informed patient of the diagnosis?医生是否告知患者诊断

☐ Yes 是
☐ No 否

Tick one 选一种	**2.1 What is the diagnosis? (ask doctor to pick closest category from list of 6 RTI)**	**2.1 诊断是什么？（请医生在六类呼吸系统感染中选择最接近诊断结果的一项）**
	1. Acute upper respiratory tract infection including:<br>. Infectious cold / **A**cute rhinitis / upper respiratory tract catarrh<br>. Acute inflammation of the throat<br>. Acute viral pharyngitis<br>. Acute viral laryngitis	急性上呼吸道感染:<br>· 传染性感冒/急性鼻炎/上呼吸道粘膜炎<br>· 喉咙急性炎症<br>· 急性病毒性咽炎<br>· 急性病毒性喉炎
	2. Acute tonsil inflammation / Tonsillitis / Pharyngitis	急性化脓性扁桃体炎/ 扁桃体炎/咽炎
	3. Acute ear inflammation / Otitis Media	急性耳朵炎症/中耳炎
	4. Acute sinusitis	急性鼻窦炎
	5. Acute tracheal/ bronchial inflammation / Acute Tracheobronchitis / Bronchitis / Lower respiratory tract infection	急性气管/支气管炎症/急性气管支气管炎/支气管炎/下呼吸道感染
	6. Pneumonia	肺炎
	. Other please state [free text box]	其他请说明[自由文本框]

**2.2 One a scale of 0 to 10, how sick do you think this patient is? (where 10 is very sick and 0 is not sick)**	**2.2 在 1 到 6 的程度中，你认为这位病人有多不舒服？ (6 表示非常不舒服， 1 表示一点点不舒服)**
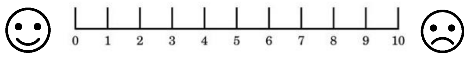	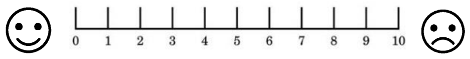

Whether practitioner has informed patient with medication? 医生是否告知患者用药情况

☐ Yes 是
☐ No 否

**2.3 Were any antibiotics prescribed?**	**2.3 抗生素是否开具？**
☐ Yes<br>☐ No	☐ 是<br>☐ 否

Participant ID: ___________________

**If Yes, first tick the method of drug administration; then specify** the name, total quantity and dosage in the table<br><br>☐ IV Antibiotics prescribed<br>☐ Oral antibiotics prescribed	**如果是，请先勾选抗生素的给药方式；再在下表中具体说明**药物通用名，总量和剂量<br><br>☐ 静脉注射抗生素<br>☐ 口服抗生素

2.4 Whether practitioner has informed patient with risks of antibiotics treatment 是否告知患者使用抗菌药物的危害

☐ Yes 告知

☐ No 未告知

IV Antibiotics prescribed:

Tick if prescribed	Medicine Name	Specification	Total quantity	Dosage
	Penicillin			
	Levofloxacin			
	Ampicillin			
	Clindamycin			
	Amikacin			
	Azithromycin			
	Metronidazole			
	Fosfomycin sodium			
	Amoxicillin			
	Erythromycin			
	Norfloxacin			
	Cefradine			

静脉注射抗生素：

如果有，请勾选	药物名称	规格	总量	剂量
	青霉素			
	左氧氟沙星			
	氨苄青霉素			
	克林霉素			
	阿米卡星(丁胺卡那霉素)			
	阿奇霉素			
	甲硝唑			
	磷霉素钠			
	阿莫西林			
	红霉素			

Participant ID: __________________

	诺氟沙星			
	头孢拉定			

Oral antibiotics prescribed:

Tick if prescribed	Antibiotics Name	Specification	Total quantity	Dosage
	Amoxicillin			
	Erythromycin			
	Norfloxacin			
	Cefradine			

口服抗生素：

如果有，请勾选	药物名称	规格	总量	剂量
	阿莫西林			
	红霉素			
	诺氟沙星			
	头孢拉定			

Other medicines prescribed:

Tick if prescribed	Medicine Name	Specification	Total quantity	Dosage	Mode of administration
	Dexamethasone				
	Tetracaine				
	chlorpheniramine				
	Aminophylline				
	Ribavirin				
	Compound paracetamol amine capsules				
	Ambroxol				
	Asthma-calming and cough-stopping patch (*pingchuan zhike pian*)				
	Qing kailing(qinghai ling)				
	Galculus Bovis and Metronidazole Capsules				

Participant ID: ___________________

	(*rengong niuhuang jia xiaocuo jiaonang*:				

其它开具的药物:

如果有，请勾选	药物名称	规格	总数量	剂量	服药方式（吊水/口服/贴等）
	地塞米松				
	丁卡因				
	扑尔敏				
	氨茶碱				
	利巴韦林				
	复方氨酚胶囊				
	盐酸氨溴索				
	平喘止咳贴片				
	清开灵				
	人工牛黄甲硝唑胶囊				

**2.4 Did the patient ask for any of these treatments?**	**2.4 患者是否主动要求过以下治疗?**
☐ IV	☐ 输液
☐ Anti-inflammation medicine/antibiotics	☐ 抗生素/消炎药
☐ Other medicines	☐ 其他药物

Any further observation note of the consultation process (Researcher’s observation):	其它有关诊疗咨询过程的观察记录?

Participant ID: ___________________

Consultation end time: ___ h ___min (24-h clock)

结束接诊时间 _____________时_______分_（24 小时制）

Whether practitioner has informed patient with standard precautions in health care ? 医生是否向患者做疾病注意事项相关宣教

- ☐ Yes 是
- ☐ No 否

Whether practitioner has informed patient with standard precautions in infection control? 医生是否想患者做预防感染相关宣教?

- ☐ Yes 是
- ☐ No 否
