# Supplementary material for: Cluster randomised controlled trial to assess a tailored intervention to reduce antibiotic prescribing in rural China: study protocol
Source: BMJ Open. 2022 Jan 3;12(1):e048267. doi: 10.1136/bmjopen-2020-048267 (PMC8724711; doi:10.1136/bmjopen-2020-048267)
Supplement: Supplementary data [file bmjopen-2020-048267supp007.pdf]

Participant ID: ___________________

WP1 Clinical consultation patient questionnaire

A field worker will administer this questionnaire at the clinical consultation at which the patient is recruited at both base line and follow up audit.

These questions must be answered by the **patient** or by direct observation by the field worker

这部分问题要求**病人**回答或者通过调查员的观察完成

1. Eligibility criteria 入选条件

Does patient have a suspected RTI?<br>. Yes<br>. No – exclude from study	病人是否疑似呼吸系统感染？<br>· 是<br>· 否—从本研究中排除
Is patient aged 18 years or older?<br>. Yes<br>. No – exclude from study	病人是否年满 18 岁？<br>· 是<br>· 否—从本研究中排除
Is today the first time the patient has sought treatment for this this illness or a repeat visit?<br>. First time consultation<br>. Repeat consultation – exclude from study	今天是病人首次还是再次为这个病来这里就诊？<br>· 首次就诊<br>· 再次就诊—从本研究中排除

Date completed: __________	调查日期：______年____月____日
Name of field worker completing proforma: __________	调查员姓名：_______________

Participant ID: ___________________

2. Participant Characteristics 研究对象基本情况

**2.1 Gender**<br>☐ Female<br>☐ Male	**2.1 性别:**<br>☐ 女性<br>☐ 男性
**2.2 Date of Birth (Year/Month/Day)**	**2.2 生日**<br>______年____月____日
**2.3 How many years of education have you completed?**<br>_________________years<br>*[Write down the exact number]*	**2.3 你上了几年学?**<br>______年<br>(写下确切的数字)

**2.4 Where do you live?**<br>*Name of town or village:*<br>____	**2.4 你住在哪里?**<br>乡镇/村的名字<br>_________________________________
**2.5 If you have lived outside your usual home residence for more than a month over the past 12 months, how many months have you been away?**<br>________months	**2.5 在过去一年，如果你离开家在外生活超过 1 个月，请给出你具体离家几个月?**<br>________个月
**2.6Is the place where you have been living urban or rural?**<br>☐ Urban<br>☐ Rural<br>☐ Not applicable	**2.7 你在外生活的地方是农村还是城市**<br>☐ 城市<br>☐ 农村<br>☐ 不适用
**2.7What is your Household registration status (*hukou*) for accessing health care benefits?**<br>☐ Urban<br>☐ Rural<br>☐ Other_____________________	**2.8 目前跟你医保挂钩的是什么户口?**<br>☐ 城市<br>☐ 农村<br>☐ 其他_____________________
**2.8What type of insurance do you have, if any?**<br>☐ New rural cooperative Medicalinsurance scheme<br>☐ Urban Employee's Medical Insurance<br>☐ Urban Residents' Medical Insurance<br>☐ Not Having Medical Insurance<br>☐ Other Type of Insurance *[please specify]*:<br>___________________________	**2.9 你参加了哪些医疗保险?**<br>☐ 新型农村合作医疗<br>☐ 城镇职工医疗保险<br>☐ 城镇居民医疗保险<br>☐ 没有医疗保险<br>☐ 其他医疗保险（请注明）:<br>__________________________

Participant ID: ___________________

3. Duration of illness and the severity 患病时长和严重程度

**Duration of your illness: _____ days**	**你的病已经持续了_______天**
**One a scale of 0 to 10, how sick do you feel? (where 10 is very sick and 0 is not sick)**	**在 0 到 10 的程度中，你认为你有多不舒服？（10 表示非常不舒服， 0 表示没有不舒服)**
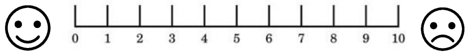	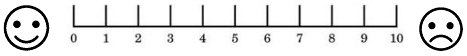

Only complete these questions if the symptoms have not been recorded on the clinician questionnaire.

请仅在医生问卷没有收集到下列症状的情况下，向病人询问下列问题。

What are yoursymptoms?您当前有何症状?

☐Blocked/ runny nose	☐鼻塞/流涕
☐ Blocked nose<br>☐ Runny nose (clear/watery discharge)<br>☐ Snotty nose (yellow/green discharge)	☐ 鼻子不通<br>☐ 流清水鼻涕<br>☐ 流脓鼻涕(黄/绿色鼻涕)
☐Coughing	☐咳嗽
☐ Dry cough<br>☐ Cough with white sputum<br>☐ Cough with yellow/green sputum	☐ 干咳<br>☐ 咳嗽带白色痰<br>☐ 咳嗽带黄/绿色脓痰
☐Throat problems	☐嗓子不舒服
☐Breathing	☐呼吸
☐ Short of breath<br>☐ Tight chest<br>☐ Difficulty breathing<br>☐ Must sit erect to breathe	☐ 气促<br>☐ 胸闷<br>☐ 呼吸不畅/困难<br>☐ 端坐呼吸<br>☐
☐Earsymptoms	☐耳部症状
☐ Blocked ears<br>☐ Tinnitus<br>☐ Pus/ fluid secretion<br>☐ Earache<br>☐ Loss of hearing	☐ 耳闷<br>☐ 耳鸣<br>☐ 流脓/液<br>☐ 耳痛<br>☐ 听力下降
☐Fever	☐发热
☐Pain	☐疼痛
☐ Headache<br>☐ Aching all-over<br>☐ Chest pain	☐ 头痛<br>☐ 全身酸痛<br>☐ 胸痛

Participant ID: ___________________

除了以上症状，你还有其他不舒服的症状么？ ____________________________

Alongside above symptoms, did you have any other symptoms that make you unwell? ______

4. Medication （用药情况）

**1**	**2**	**3**	**4**	**5**
**Have you taken any medication for this illness before coming here?**<br>**Yes/No** （在为这个病来就诊前，你有服用任何药物么？<br><br>**If yes, please answer column 2 and 3**<br>如果回答 是， 请回答问题 2 和 3<br><br>**If No, please answer column 4 and 5**<br>如果回答 没有，请回答问题 4 和 5	**Days have you taken it?**<br>（你服用药物几天？）	**Where did you got it?**<br>（你从那里获得服用的药物？）<br>1, pharmacies outside the hospitals （医院外的药房）<br>2, family or relatives or friends （家人和朋友给的）<br>3 Residual drugs at home （之前用药剩下的）	**Have you had the medication in the past six month before this illness?**<br>（在这个病的前 6 个月里，你有服用任何药物么？<br>**Yes/No** 是/否<br>**If yes, please answer column 5**<br>（回答是，请继续回答问题 5）	**How many course have you taken?**<br>（你服用几个疗程的药物）<br><br>1,1-3 times<br>1-3 个疗程<br>2, 4-6 times<br>4-6 个疗程<br>3, 6 times<br>6 个疗程<br>4, more than 6 times<br>超过 6 个疗程

5.Patient Satisfaction 患者满意度

**One a scale of 0 to 10, how satisfied were you with your visit to the doctor today? (where 10 is very satisfied and 0 is not satisfied at all)**	**在 0 到 10 的程度中，你今天在这里看病满意吗？ (10 表示非常满意， 0 表示完全不满意)**
☹ 0 1 2 3 4 5 6 7 8 9 10 ☺	☹ 0 1 2 3 4 5 6 7 8 9 10 ☺

Participant ID: ___________________

EQ-5D-5L

Health Questionnaire

English version for the UK

VERSION FOR INTERVIEWER ADMINISTRATION

Note to interviewer: although allowance should be made for the interviewer's particular style of speaking, the wording of the questionnaire instructions should be followed as closely as possible. In the case of the EQ-5D-5L descriptive system on page 2 of the questionnaire, the precise wording must be followed.

If the respondent has difficulty choosing a response, or asks for clarification, the interviewer should repeat the question word for word and ask the respondent to answer in a way that most closely resembles his or her thoughts about his or her health today.

INTRODUCTION

(Note to interviewer: please read the following to the respondent.)

We are trying to find out what you think about your health. I will explain what to do as I go along, but please interrupt me if you do not understand something or if things are not clear to you. There are no right or wrong answers. We are interested only in your personal view.

First, I am going to read out some questions. Each question has a choice of five answers. Please tell me which answer best describes your health TODAY.

Do not choose more than one answer in each group of questions.

Participant ID: ___________________

(Note to interviewer: first read all five options for each question. Then ask the respondent to choose which one applies to him/herself. Repeat the question and options if necessary. Mark the appropriate box under each heading. You may need to remind the respondent regularly that the timeframe is TODAY.)

EQ-5D DESCRIPTIVE SYSTEM

MOBILITY

First, I would like to ask you about mobility. Would you say that:

1. You have no problems in walking about? ❑
2. You have slight problems in walking about? ❑
3. You have moderate problems in walking about? ❑
4. You have severe problems in walking about? ❑
5. You are unable to walk about? ❑

SELF-CARE

Next, I would like to ask you about self-care. Would you say that:

1. You have no problems washing or dressing yourself? ❑
2. You have slight problems washing or dressing yourself? ❑
3. You have moderate problems washing or dressing yourself? ❑
4. You have severe problems washing or dressing yourself? ❑
5. You are unable to wash or dress yourself? ❑

USUAL ACTIVITIES

Next, I would like to ask you about usual activities, for example work, study, housework, family or leisure activities. Would you say that:

1. You have no problems doing your usual activities? ❑
2. You have slight problems doing your usual activities? ❑
3. You have moderate problems doing your usual activities? ❑
4. You have severe problems doing your usual activities? ❑
5. You are unable to do your usual activities? ❑

PAIN / DISCOMFORT

Next, I would like to ask you about pain or discomfort. Would you say that:

1. You have no pain or discomfort? ❑
2. You have slight pain or discomfort? ❑
3. You have moderate pain or discomfort? ❑

Participant ID: ___________________

4. You have severe pain or discomfort? ❑
5. You have extreme pain or discomfort? ❑

The best health you can imagine

ANXIETY / DEPRESSION

Finally, I would like to ask you about anxiety or depression. Would you say that:

1. You are not anxious or depressed? ❑
2. You are slightly anxious or depressed? ❑
3. You are moderately anxious or depressed? ❑
4. You are severely anxious or depressed? ❑
5. You are extremely anxious or depressed? ❑

EQ-5D VAS

- **Now, I would like to ask you to say how good or bad your health is TODAY.**
- **I would like you to try to picture in your mind a scale that looks like a thermometer.** *(Note to interviewer: if interviewing face-to-face, please show the person the VAS scale.)*
- **The best health you can imagine is marked 100 (one hundred) at the top of the scale and the worst health you can imagine is marked 0 (zero) at the bottom.**
- **I would now like you to tell me the point on this scale where you would put your health TODAY.** *(Note to interviewer: mark the scale at the point indicating the respondent's 'health today'. Now, please write the number you marked on the scale in the box below.)*

Participant ID: ____________________

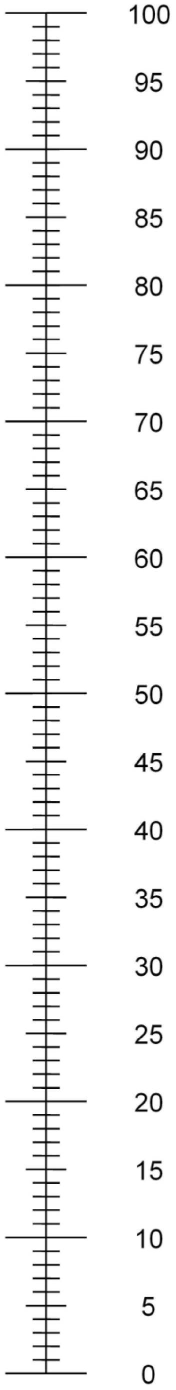

THE RESPONDENT'S HEALTH TODAY = [ ]

Thank you for taking the time to answer these questions.

The worst health you can imagine
