# Supplementary material for: Cluster randomised controlled trial to assess a tailored intervention to reduce antibiotic prescribing in rural China: study protocol
Source: BMJ Open. 2022 Jan 3;12(1):e048267. doi: 10.1136/bmjopen-2020-048267 (PMC8724711; doi:10.1136/bmjopen-2020-048267)

WP1 Patient telephone questionnaire – Day 7

A field worker will conduct phone interviews with the patients at 7, 14 and 21 days. Patients will also be asked the questions from the EQ5D.

调查员将在第 7， 14 和 21 天对病人进行电话访谈。除了以下问题，病人还会被问道一些 EQ5D 问卷中的问题。

1.Basic information 基本情况

**Patient ID**	**病人编号**
Days since baseline consultation<br>☐ 7 days<br>☐ 14 days<br>☐ 21 days	这是最初看病之后的第几天？<br>☐ 7 天<br>☐ 14 天<br>☐ 21 天
Date completed: __________	调查日期： ______年____月____日
Name of field worker completing proforma:<br>__________	调查员姓名： _______________

2. Continuing Illness Severity 疾病持续严重程度

**One a scale of 0 to 10, how sick do you feel? (where 10 is very sick and 0 is not sick)**	**在 0 到 10 的程度中，你认为你有多不舒服？（10 表示非常不舒服， 0 表示没有不舒服）**
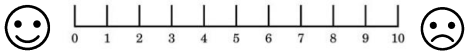	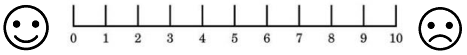

3. Patient view of leaflet 病人对宣传手册的看法

Did you have a look of the patient leaflet?<br>☐ Yes<br>☐ No<br>☐ Did not get leaflet	你是否看了病人宣传手册？<br>☐ 是<br>☐ 否<br>☐ 没有拿到
How easy was the patient leaflet to understand?<br>☐ Very easy, I understood all of it<br>☐ Easy, I understood most of it<br>☐ Not Easy, I only undersood a little<br>☐ Too hard, I did not understand any<br>☐ I am not able to read	你觉得病人宣传手册有多容易理解？<br>☐ 非常容易，我可以全部理解<br>☐ 容易，我可以理解大部分<br>☐ 不容易，我只能理解小部分<br>☐ 非常难，我完全不能理解<br>☐ 我读不了宣传手册

Cong W, *et al. BMJ Open* 2022; 12:e048267. doi: 10.1136/bmjopen-2020-048267

Did the leaflet change your ideas or beliefs about what illnesses need anti-inflammation medicine/ antibiotics?<br>☐ Yes<br>☐ No	病人宣传手册对您关于什么样的<br>疾病需要使用消炎药/抗生素的想法有改变吗?<br>☐ 有<br>☐ 没有

Cong W, *et al. BMJ Open* 2022; 12:e048267. doi: 10.1136/bmjopen-2020-048267

4. Antibiotic Consumption 抗生素服用情况

Were you prescribed oral anti-bacterial/anti-inflammation medicine in your baseline consultation?<br>☐ Yes (if yes, field worker will ask the two questions below in this table)<br>☐ No<br>☐ Not applicable	您在最初看病时是否有开口服抗菌药物/消炎药吗？<br>☐ 是（如果有，请继续问下面两个问题）<br>☐ 否<br>☐ 不清楚
How many days and times per day did you actually take a dose since you saw the Dr?<br>☐ [Number] ________of days<br>☐ [Number]________ times / day	从那之后你已经吃了多少天？每天实际吃多少次？<br>☐ （ ）天<br>☐ （ ）次/天
What was the dose did you take every time?	您每次吃的剂量是多少？
Have you stopped taking the anti-bacterial/anti-inflammation medicine that you were prescribed?<br>☐ Yes<br>☐ No<br>☐ Not applicable	你是否已经停止服用给你开的抗菌药物/消炎药了？<br>☐ 是<br>☐ 否<br>☐ 不清楚

Cong W, *et al. BMJ Open* 2022; 12:e048267. doi: 10.1136/bmjopen-2020-048267

EQ-5D-5L

Health Questionnaire

English version for the UK

VERSION FOR INTERVIEWER ADMINISTRATION

Note to interviewer: although allowance should be made for the interviewer's particular style of speaking, the wording of the questionnaire instructions should be followed as closely as possible. In the case of the EQ-5D-5L descriptive system on page 2 of the questionnaire, the precise wording must be followed.

If the respondent has difficulty choosing a response, or asks for clarification, the interviewer should repeat the question word for word and ask the respondent to answer in a way that most closely resembles his or her thoughts about his or her health today.

INTRODUCTION

(Note to interviewer: please read the following to the respondent.)

We are trying to find out what you think about your health. I will explain what to do as I go along, but please interrupt me if you do not understand something or if things are not clear to you. There are no right or wrong answers. We are interested only in your personal view.

First, I am going to read out some questions. Each question has a choice of five answers. Please tell me which answer best describes your health TODAY.

Do not choose more than one answer in each group of questions.

(Note to interviewer: first read all five options for each question. Then ask the respondent to choose which one applies to him/herself. Repeat the question and options if necessary. Mark the appropriate box under each heading. You may need to remind the respondent regularly that the timeframe is TODAY.)

Cong W, *et al. BMJ Open* 2022; 12:e048267. doi: 10.1136/bmjopen-2020-048267

EQ-5D DESCRIPTIVE SYSTEM

MOBILITY

First, I would like to ask you about mobility. Would you say that:

1. You have no problems in walking about? ❑
2. You have slight problems in walking about? ❑
3. You have moderate problems in walking about? ❑
4. You have severe problems in walking about? ❑
5. You are unable to walk about? ❑

SELF-CARE

Next, I would like to ask you about self-care. Would you say that:

1. You have no problems washing or dressing yourself? ❑
2. You have slight problems washing or dressing yourself? ❑
3. You have moderate problems washing or dressing yourself? ❑
4. You have severe problems washing or dressing yourself? ❑
5. You are unable to wash or dress yourself? ❑

USUAL ACTIVITIES

Next, I would like to ask you about usual activities, for example work, study, housework, family or leisure activities. Would you say that:

1. You have no problems doing your usual activities? ❑
2. You have slight problems doing your usual activities? ❑
3. You have moderate problems doing your usual activities? ❑
4. You have severe problems doing your usual activities? ❑
5. You are unable to do your usual activities? ❑

PAIN / DISCOMFORT

Next, I would like to ask you about pain or discomfort. Would you say that:

1. You have no pain or discomfort? ❑
2. You have slight pain or discomfort? ❑
3. You have moderate pain or discomfort? ❑
4. You have severe pain or discomfort? ❑
5. You have extreme pain or discomfort? ❑

ANXIETY / DEPRESSION

Finally, I would like to ask you about anxiety or depression. Would you say that:

1. You are not anxious or depressed? ❑
2. You are slightly anxious or depressed? ❑
3. You are moderately anxious or depressed? ❑
4. You are severely anxious or depressed? ❑
5. You are extremely anxious or depressed? ❑

Cong W, *et al. BMJ Open* 2022; 12:e048267. doi: 10.1136/bmjopen-2020-048267

EQ-5D VAS

- **Now, I would like to ask you to say how good or bad your health is TODAY.**
- **I would like you to try to picture in your mind a scale that looks like a thermometer.**
  (Note to interviewer: if interviewing face-to-face, please show the person the VAS scale.)
- **The best health you can imagine is marked 100 (one hundred) at the top of the scale and the worst health you can imagine is marked 0 (zero) at the bottom.**
- **I would now like you to tell me the point on this scale where you would put your health TODAY.**
  (Note to interviewer: mark the scale at the point indicating the respondent's 'health today'. Now, please write the number you marked on the scale in the box below.)

THE RESPONDENT'S HEALTH TODAY = [ ]

Thank you for taking the time to answer these questions.

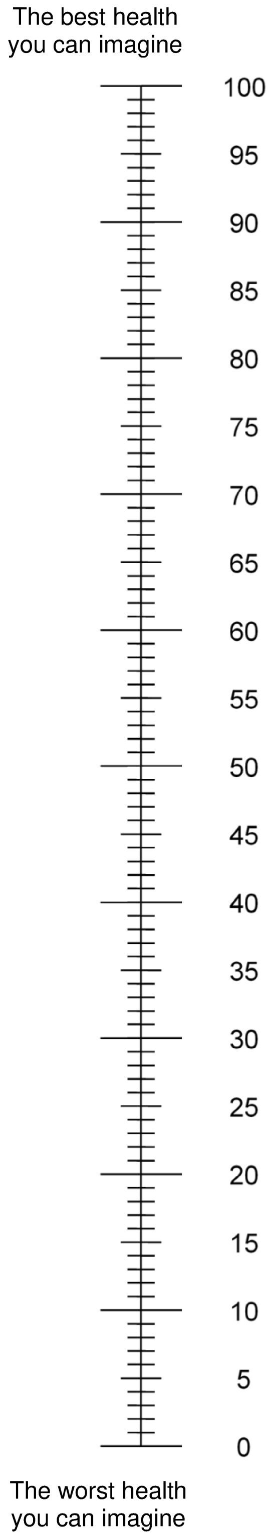

Supplement: Supplementary data [file bmjopen-2020-048267supp008.pdf]
