# Supplementary material for: Cluster randomised controlled trial to assess a tailored intervention to reduce antibiotic prescribing in rural China: study protocol
Source: BMJ Open. 2022 Jan 3;12(1):e048267. doi: 10.1136/bmjopen-2020-048267 (PMC8724711; doi:10.1136/bmjopen-2020-048267)
Supplement: Supplementary data [file bmjopen-2020-048267supp010.pdf]

WP1 Patient telephone questionnaire – Day 21

A field worker will conduct phone interviews with the patients at 7, 14 and 21 days. Patients will also be asked the questions from the EQ5D.

调查员将在第 7，14 和 21 天对病人进行电话访谈。除了以下问题，病人还会被问道一些 EQ5D 问卷中的问题。

1.Basic information 基本情况

**Patient ID**	**病人编号**
Days since baseline consultation<br>☐ 7 days<br>☐ 14 days<br>☐ 21 days	这是最初看病之后的第几天？<br>☐ 7 天<br>☐ 14 天<br>☐ 21 天
Date completed: __________	调查日期： ______年____月____日
Name of field worker completing proforma:<br>__________	调查员姓名： ________________

2. Continuing Illness Severity 疾病持续严重程度

**One a scale of 0 to 10, how sick do you feel? (where 10 is very sick and 0 is not sick)**	**在 0 到 10 的程度中，你认为你有多不舒服？（10 表示非常不舒服， 0 表示没有不舒服）**
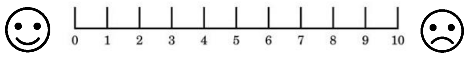	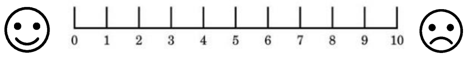

3. Antibiotic Consumption 抗生素服用情况

Were you prescribed oral anti-bacterial/anti-inflammation medicine in your baseline consultation?<br>☐ Yes (if yes, field worker will ask the two questions below in this table)<br>☐ No<br>☐ Not applicable	您在最初看病时是否有开口服抗菌药物/消炎药吗？<br>☐ 是（如果有，请继续问下面两个问题）<br>☐ 否<br>☐ 不清楚
How many days and times per day did you actually take a dose since you saw the Dr?<br>☐ [Number] _________of days<br>☐ [Number]_________ times / day	从那之后你已经吃了多少天？每天实际吃多少次？<br>☐ （ ）天<br>☐ （ ）次/天
Have you stopped taking the anti-	你是否已经停止服用给你开的抗菌药物/消炎

Cong W, *et al. BMJ Open* 2022; 12:e048267. doi: 10.1136/bmjopen-2020-048267

bacterial/anti-inflammation medicine that you were prescribed?<br><br>☐ Yes<br>☐ No<br>☐ Not applicable	药了？<br><br>☐ 是<br>☐ 否<br>☐ 不清楚

4. Resource Use for Economic Evaluation

Additional Health Care Utilisation 其他卫生服务的使用

**Did you seek treatment for your illness from any of these since we talked to you in your consultation?**	**How many times did you visit there?**	**How much did you pay in total?**	**How much did you pay from your own pocket?**
Pharmacy			
Other township health centre			
Village clinics			
Private clinics			

**自从上次看病之后，你是否还因为你的病去下列地方寻医问药?**	**你去了几次?**	**总共花了多少钱?**	**你自费付了多少钱?**
药店			
其他乡镇卫生院			
村卫生室			
个体诊所			

**Did you seek secondary care for your illness since we talked to you in your consultation?**	**How many times did you visit?**	**How much did you pay in total**	**How much did you pay from your own pocket?**
Outpatient			
Inpatient			
Accident and emergency			

**自从上次看病之后，你是否因为你的病去医院就诊?**	**你去了几次?**	**总共花了多少钱?**	**你自费付了多少钱?**
门诊			
住院			
急诊			

**After the consultation when**	**Prescribed**	**Bought**	**Obtained from**	**How much**	**How much**

Cong W, *et al. BMJ Open* 2022; 12:e048267. doi: 10.1136/bmjopen-2020-048267

we recruited you, have you had any treatment for this illness that wasn't prescribed in your consultation?	by other doctors	from pharmacy	other source (home, family, friends)	did you pay in total?	did you pay from your own pocket?
**Anti-bacterial/anti-inflammation medicine**					
☐ Amoxicillin	Y/N	Y/N	Y/N	¥	¥
☐ Erythromycin	Y/N	Y/N	Y/N	¥	¥
☐ Cefradine	Y/N	Y/N	Y/N	¥	¥
☐ Levofloxacin	Y/N	Y/N	Y/N	¥	¥
☐ Cefuroxime	Y/N	Y/N	Y/N	¥	¥
☐ Other anti-bacterial/anti-inflammation medicine [please specify]	Y/N	Y/N	Y/N	¥	¥
**Other medicines/treatment**					
☐ Paracetamol	Y/N	Y/N	Y/N	¥	¥
☐ Ganmaoling Granules（e.g. 999 cold remedy）	Y/N	Y/N	Y/N	¥	¥
☐ Qingkailing	Y/N	Y/N	Y/N	¥	¥
☐ Yinhuang Granules	Y/N	Y/N	Y/N	¥	¥
☐ Xiongju Shangqing	Y/N	Y/N	Y/N	¥	¥
☐ Lianhua (lotus) Qingwen capsules	Y/N	Y/N	Y/N	¥	¥
☐ Cough syrup	Y/N	Y/N	Y/N	¥	¥
☐ Ambroxol Hydrochloride oral suspension	Y/N	Y/N	Y/N	¥	¥
☐ Keteling	Y/N	Y/N	Y/N	¥	¥
☐ Compound liquorice tablets	Y/N	Y/N	Y/N	¥	¥
☐ Vitamin C	Y/N	Y/N	Y/N	¥	¥
☐ Crystal sugar snow pear	Y/N	Y/N	Y/N	¥	¥
☐ Ginger	Y/N	Y/N	Y/N	¥	¥
☐ Mugwort	Y/N	Y/N	Y/N	¥	¥
☐ Pipa (loquat)	Y/N	Y/N	Y/N	¥	¥
☐ Orange peel granules	Y/N	Y/N	Y/N	¥	¥
☐ Other [please specify]	Y/N	Y/N	Y/N	¥	¥

在最初看病之后，你是否自己用过了其他不是这次看病是所开具的药物?	其他医生开具	从药店购买	其他方法获得（家中储存，家人，朋友）	这些药总共多少钱?	你自费付了多少钱?
**抗菌药/消炎药**					
☐ 阿莫西林	是/否	是/否	是/否	¥	¥
☐ 红霉素	是/否	是/否	是/否	¥	¥
☐ 头孢拉定	是/否	是/否	是/否	¥	¥

☐ 左氧氟沙星	是/否	是/否	是/否	¥	¥
☐ 头孢呋辛	是/否	是/否	是/否	¥	¥
☐ 其他抗菌药/消炎药（请注明）	是/否	是/否	是/否	¥	¥
**其他药物**					
☐ 扑热息痛	是/否	是/否	是/否	¥	¥
☐ 感冒灵颗粒（如 999 感冒灵）	是/否	是/否	是/否	¥	¥
☐ 清开灵	是/否	是/否	是/否	¥	¥
☐ 银黄颗粒	是/否	是/否	是/否	¥	¥
☐ 芎菊上清	是/否	是/否	是/否	¥	¥
☐ 莲花清瘟胶囊	是/否	是/否	是/否	¥	¥
☐ 咳嗽糖浆	是/否	是/否	是/否	¥	¥
☐ 盐酸氨溴索口服液	是/否	是/否	是/否	¥	¥
☐ 咳特灵	是/否	是/否	是/否	¥	¥
☐ 复方甘草片	是/否	是/否	是/否	¥	¥
☐ 维生素 C	是/否	是/否	是/否	¥	¥
☐ 冰糖雪梨	是/否	是/否	是/否	¥	¥
☐ 姜	是/否	是/否	是/否	¥	¥
☐ 艾草	是/否	是/否	是/否	¥	¥
☐ 琵琶	是/否	是/否	是/否	¥	¥
☐ 陈皮颗粒	是/否	是/否	是/否	¥	¥
☐ 其他（请注明）	是/否	是/否	是/否	¥	¥

Productivity loss 生产力下降

**If you work, how many days in total have you taken off work due to this illness?**	如果你工作，那么因为这次生病你总共耽误了几天上班？
{number} days	（ ）天
**If you are in full time education, please state how many days in total have you missed your education?**	如果你是学生，那么因为这次生病你总共耽误了几天上学？
{number} days	（ ）天

Cong W, *et al. BMJ Open* 2022; 12:e048267. doi: 10.1136/bmjopen-2020-048267

EQ-5D-5L

Health Questionnaire

English version for the UK

VERSION FOR INTERVIEWER ADMINISTRATION

Note to interviewer: although allowance should be made for the interviewer's particular style of speaking, the wording of the questionnaire instructions should be followed as closely as possible. In the case of the EQ-5D-5L descriptive system on page 2 of the questionnaire, the precise wording must be followed.

If the respondent has difficulty choosing a response, or asks for clarification, the interviewer should repeat the question word for word and ask the respondent to answer in a way that most closely resembles his or her thoughts about his or her health today.

INTRODUCTION

(Note to interviewer: please read the following to the respondent.)

We are trying to find out what you think about your health. I will explain what to do as I go along, but please interrupt me if you do not understand something or if things are not clear to you. There are no right or wrong answers. We are interested only in your personal view.

First, I am going to read out some questions. Each question has a choice of five answers. Please tell me which answer best describes your health TODAY.

Do not choose more than one answer in each group of questions.

(Note to interviewer: first read all five options for each question. Then ask the respondent to choose which one applies to him/herself. Repeat the question and options if necessary. Mark the appropriate box under each heading. You may need to remind the respondent regularly that the timeframe is TODAY.)

EQ-5D DESCRIPTIVE SYSTEM

MOBILITY

First, I would like to ask you about mobility. Would you say that:

1. You have no problems in walking about? ❑
2. You have slight problems in walking about? ❑
3. You have moderate problems in walking about? ❑
4. You have severe problems in walking about? ❑
5. You are unable to walk about? ❑

SELF-CARE

Next, I would like to ask you about self-care. Would you say that:

1. You have no problems washing or dressing yourself? ❑
2. You have slight problems washing or dressing yourself? ❑
3. You have moderate problems washing or dressing yourself? ❑
4. You have severe problems washing or dressing yourself? ❑
5. You are unable to wash or dress yourself? ❑

USUAL ACTIVITIES

Next, I would like to ask you about usual activities, for example work, study, housework, family or leisure activities. Would you say that:

1. You have no problems doing your usual activities? ❑
2. You have slight problems doing your usual activities? ❑
3. You have moderate problems doing your usual activities? ❑
4. You have severe problems doing your usual activities? ❑
5. You are unable to do your usual activities? ❑

PAIN / DISCOMFORT

Next, I would like to ask you about pain or discomfort. Would you say that:

1. You have no pain or discomfort? ❑
2. You have slight pain or discomfort? ❑
3. You have moderate pain or discomfort? ❑
4. You have severe pain or discomfort? ❑
5. You have extreme pain or discomfort? ❑

ANXIETY / DEPRESSION

Finally, I would like to ask you about anxiety or depression. Would you say that:

1. You are not anxious or depressed? ❑
2. You are slightly anxious or depressed? ❑
3. You are moderately anxious or depressed? ❑
4. You are severely anxious or depressed? ❑
5. You are extremely anxious or depressed? ❑

Cong W, *et al. BMJ Open* 2022; 12:e048267. doi: 10.1136/bmjopen-2020-048267

EQ-5D VAS

- **Now, I would like to ask you to say how good or bad your health is TODAY.**
- **I would like you to try to picture in your mind a scale that looks like a thermometer.**
  (Note to interviewer: if interviewing face-to-face, please show the person the VAS scale.)
- **The best health you can imagine is marked 100 (one hundred) at the top of the scale and the worst health you can imagine is marked 0 (zero) at the bottom.**
- **I would now like you to tell me the point on this scale where you would put your health TODAY.**
  (Note to interviewer: mark the scale at the point indicating the respondent's 'health today'. Now, please write the number you marked on the scale in the box below.)

THE RESPONDENT'S HEALTH TODAY = ☐

Thank you for taking the time to answer these questions.

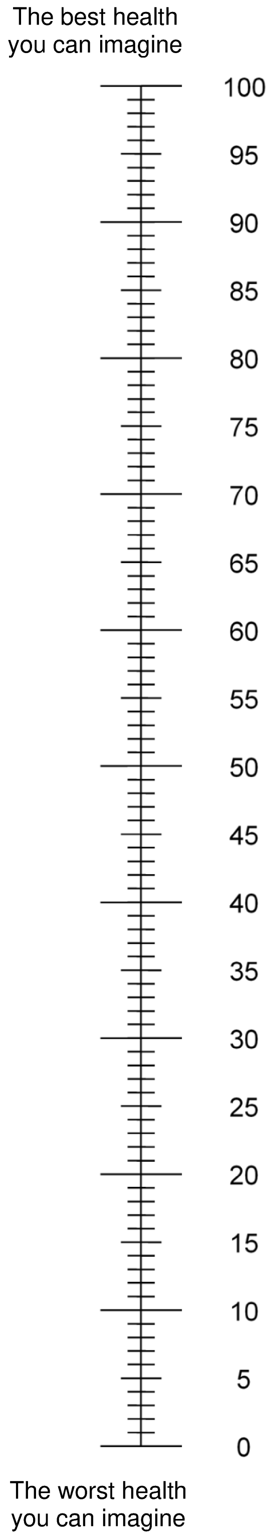

Cong W, *et al. BMJ Open* 2022; 12:e048267. doi: 10.1136/bmjopen-2020-048267
