# Supplementary material for: Cluster randomised controlled trial to assess a tailored intervention to reduce antibiotic prescribing in rural China: study protocol
Source: BMJ Open. 2022 Jan 3;12(1):e048267. doi: 10.1136/bmjopen-2020-048267 (PMC8724711; doi:10.1136/bmjopen-2020-048267)
Supplement: Author's manuscript [file bmjopen-2020-048267.draft_revisions.pdf]

BMJ Open

BMJ Open is committed to open peer review. As part of this commitment we make the peer review history of every article we publish publicly available.

When an article is published we post the peer reviewers' comments and the authors' responses online. We also post the versions of the paper that were used during peer review. These are the versions that the peer review comments apply to.

The versions of the paper that follow are the versions that were submitted during the peer review process. They are not the versions of record or the final published versions. They should not be cited or distributed as the published version of this manuscript.

BMJ Open is an open access journal and the full, final, typeset and author-corrected version of record of the manuscript is available on our site with no access controls, subscription charges or pay-per-view fees (http://bmjopen.bmj.com).

If you have any questions on BMJ Open's open peer review process please email info.bmjopen@bmj.com

BMJ Open

A cluster randomised controlled trial to assess a tailored intervention to reduce antibiotic prescribing in rural China – Study protocol

Journal:	*BMJ Open*
Manuscript ID	bmjopen-2020-048267
Article Type:	Protocol
Date Submitted by the Author:	23-Dec-2020
Complete List of Authors:	Cong, Wenjuan; University of Bristol, Population Health Sciences<br>Chai, Jing; Anhui Medical University, School of Health Service Management<br>Zhao, Linhai; Anhui Medical University, School of Health Services Management<br>Cabral, Christie; University of Bristol, Population Health Sciences<br>Yardley, Lucy; University of Bristol, School of Psychological Sciences<br>Yao, Guiqing; University of Leicester Department of Health Sciences<br>Zhang, Tingting; University of Bristol, Population Health Sciences<br>Cheng, Jing; Anhui Medical University, School of Health Services Management<br>Shen, XingRong; Anhui Medical University, School of Health Service Management<br>Liu, Rong; Anhui Medical University, School of Health Services Management<br>Little, Paul; University of Southampton, Primary Care and Population Science<br>Stuart, Beth; University of Southampton, Primary Care and Population Science<br>Hu, Xiaowen; Anhui Medical University, School of Health Service Management<br>Sun, Ye-Huan; Anhui Medical University, Department of Epidemiology and Biostatistics<br>Oliver, Isabel; Public Health England South Region, National Infection Service<br>Zheng, Bo; Peking University First Hospital, Institute of Clinical Pharmacology<br>Lambert, Helen; University of Bristol, Population Health Sciences<br>Wang, DeBin; Anhui Medical University, School of Health Services Management
Keywords:	PRIMARY CARE, Respiratory infections < THORACIC MEDICINE, GENERAL MEDICINE (see Internal Medicine)

For peer review only - http://bmjopen.bmj.com/site/about/guidelines.xhtml

A cluster randomised controlled trial to assess a tailored intervention to reduce antibiotic prescribing in rural China – Study protocol

Wenjuan Cong[1#], Jing Chai[2#], Linhai Zhao[2], Christie Cabral[1*], Lucy Yardley[3], Guiqing Yao[4], Tingting Zhang[1], Jing Cheng[2], Xinrong Sheng[2], Rong Liu[2], Paul Little[5], Beth Stuart[5], Xiaowen Hu[6], Yehuan Sun[6], Isabel Oliver[7], Bo Zheng[8], Helen Lambert[1], Debin Wang[2*]

Abstract

Introduction: Up to 80% of patients with respiratory-tract infections (RTI) attending village clinics in rural areas of China are prescribed antibiotics, many of which are unnecessary. Since 2009, China has implemented several policies to try to reduce inappropriate antibiotic use however antibiotic prescribing remains high in village clinics.

Methods and analysis: A cluster-randomised controlled trial will be carried out to estimate the effectiveness and cost effectiveness of a complex intervention in reducing antibiotic prescribing at village clinics in Anhui Province, China. 64 village clinics will be randomised at a 1:1 ratio to the intervention or usual care arms. In the intervention group, village practitioners will receive an intervention comprising: i) training to support appropriate antibiotic prescribing for RTI, ii) a computer-based decision support system, iii) virtual peer support, iv) a leaflet for patients and v) a letter of commitment to optimise antibiotic use to display in their clinic.

The primary outcome is the percentage of antibiotics (intravenous and oral) prescribed for RTI patients. Secondary outcomes include patient symptom severity and duration, recovery status, satisfaction, antibiotic consumption. A full economic evaluation will be conducted within the trial period. Costs and savings for both clinics and patients will be considered and quality of life will be measured by EuroQoL (EQ-5D-5L).

A qualitative process evaluation will explore practitioner and patient views and experiences of trial processes, intervention fidelity and acceptability, and barriers and facilitators to implementation.

Ethics and dissemination: Ethical approval was obtained from the Biomedical Research Ethics Committee of Anhui Medical University (Ref: 20180259); the study has undergone due diligence checks and is registered at the University of Bristol (Case No: 2020-3137). The research findings will also be disseminated to stakeholders through conferences and peer-reviewed journals in China, the UK and internationally.

Trial Registration: This trial has been registered at ISRCTN (ISRCTN30652037).

[#]WC and JC contributed equally

[*]DW and CC contributed equally

Corresponding Author: Prof Debin Wang; Contact Email: dbwang@vip.sina.com

For peer review only

UOB Confidential & Sensitive

Strengths and limitations of this study

- This is a large cluster-randomised trial which will be able to determine whether the intervention is effective and cost effective in reducing antibiotic prescribing for patients with RTI in village clinics compared with usual care.
- The study results will inform policy and clinical practice whether the complex intervention is good value for money and whether it should be adopted in reducing antibiotics prescribing in the rural China.
- The Covid-19 pandemic is likely to impact trial recruitment and we may have different characteristics of RTI patients receiving treatment in the village clinics.

Introduction

Antimicrobial resistance (AMR) specifically refers to the evolution of drug-resistant micro-organisms, including bacteria, which is driven by high rates of antibiotic use and is a pressing problem for global health and development.[1-3] If resistance continues to increase, common bacterial infections will eventually become untreatable.[4-5]

Since 2009, China has implemented the national essential medicines scheme and zero-mark-up policies to try to reduce inappropriate antibiotic use. [10-11] In 2012, the Chinese Ministry of Health issued a regulation to limit antibiotic prescribing to 20% of outpatient prescriptions in all patients.[12] However, the antibiotic prescribing rate in healthcare facilities in less developed or rural areas remains as high as 80%.[13-18]

In China patients can choose to go to the outpatient department of any health facility without a referral letter,[19] however, rural residents mainly seek health care from village clinics. If patients develop severe symptoms, they are more likely to go directly to the outpatient clinic in the township health centre or county hospital.[19-20] Many patients attend village clinics with respiratory tract infections (RTIs) such as common cold, sore throat, and acute bronchitis and are frequently prescribed antibiotics even though most cases are likely to be viral and antibiotics are therefore unnecessary [21-24] Our previous study found that the antibiotic prescribing rate for RTI patients was up to 85% in rural Anhui Province.[25] In addition to the implications for AMR, there is a cost to patients [26-27] for extra health resource utilization, most of which would not be reimbursed through national medical insurance schemes. Therefore, reducing antibiotic prescribing in village clinics is likely to be both safe and beneficial to patients.

Village clinics in China are staffed with health practitioners who are usually referred to as “village practitioners” but would not have a degree in medicine. Village practitioners are required to undergo basic training in health care provided by the local health care authority issued with a medical practice licence. Antibiotic prescribing in village clinics is influenced by uncertainty about which RTI need antibiotics and by widespread beliefs that common infections should be treated with antibiotics.[19] Practitioners are also concerned about losing patients and believe that prescribing antibiotics helps maintain good relationships with their patients. Treating patients with antibiotics through intravenous (IV) drips can generate additional income for village clinics, which are effectively private practices that also deliver some (poorly funded) government public health services.[28] Public campaigns on antimicrobial stewardship (AMS), [29-30] are very limited in rural areas and there is little technical support

For peer review only - http://bmjopen.bmj.com/site/about/guidelines.xhtml
UOB Confidential & Sensitive

for village practitioners from senior clinical consultants and healthcare authorities, which makes it difficult for village practitioners to change norms of high antibiotic use.

This study will aim to determine whether the intervention safely reduces antibiotic prescribing for patients with RTI in village clinics. The study will provide evidence of effectiveness, value for money and barriers and facilitators to implementation that can inform policy and clinical practice.

Methods and Analysis

Aims and Objectives

This study aims to evaluate the effectiveness of a complex intervention aimed at village healthcare practitioners and patients, to reduce antibiotic prescribing for respiratory-tract infections (RTIs) compared with usual care in village clinics.

The primary outcome will be the percentage of antibiotics (intravenous and oral) prescribed for RTI patients.

Secondary objectives are to:

- estimate the cost-effectiveness of the intervention and the cost implications for both village clinics and patients.
- evaluate the impact of reduced antibiotic use on patient satisfaction with care.
- evaluate the impact of reduced antibiotic use on patient symptom severity, duration and recovery rates.
- investigate patient antibiotic consumption rates (prescribed or obtained from another source.
- understand practitioner and patient views of the intervention and barriers and facilitators to implementation.

Trial design

This is a pragmatic, two-armed (intervention vs. usual care) trial with an embedded cost-effectiveness and qualitative evaluations, with randomisation at the village clinic level, using observed antibiotic prescribing to assess effectiveness.

Participants and recruitment procedures

Selection of village clinics

We will recruit 64 village clinics, each from a different non-contiguous township area and geographically dispersed across Anhui Province. Village clinics in the same township areas are often involved in joint training so by ensuring each recruited village clinic is from a different township area, we seek to minimise contamination between arms. Local county health authorities will provide a list of townships in their area and the research team will select two non-contiguous townships at random (by selecting the first randomly, excluding all that share a boundary and then selecting the 2nd at random from the remainder). Local county health authorities will screen the selected townships and may exclude selected townships, for which a reason will be recorded. With the support of the local county health authorities, we will request a list of the village clinics that have a medical practice licence from the township health authorities.

We will recruit the village clinic that serves the largest population in each township area. There has been a large decrease in RTI patients presenting to village clinics due to the Covid-19 pandemic. Currently patients with suspected Covid-19 symptoms (specifically patients with a temperature of more than 37.3 degree) must be referred to specialist hospitals for treatment. Preliminary fieldwork found that although there are still RTI patients seeking care at village clinics, the numbers are much

For peer review only - http://bmjopen.bmj.com/site/about/guidelines.xhtml
UOB Confidential & Sensitive

lower than were observed in our previous study. Therefore, we will mainly recruit the clinic serving the largest population in order to complete recruitment within the current project resources. To enable some understanding of the likely selection bias, we will also recruit a small proportion of smaller clinics from the quartile serving the smallest populations.

Recruitment of village practitioners

All the practitioners working in the recruited village clinics will be invited to participate in the study (in some this may be a single practitioner). Once village practitioners are identified and recruited, informed consent (Appendix 1, Supplementary material) will be obtained immediately.

Exclusion criteria
1, Village practitioners who have basic computer skills
2, Village practitioners who are not involved in clinical diagnosis and medicine prescribing

The background information of participating clinics and village practitioners will be collected. A senior practitioner in the recruited clinic will complete a clinic background information questionnaire (Appendix 2, Supplementary material); and all village practitioners working in the clinic will give their demographic data in a practitioner background information questionnaire (Appendix 3, Supplementary material).

A baseline audit of 2000 RTI patients across the 64 village clinics will be conducted. This baseline audit will collect all primary and secondary outcome data, including the antibiotic prescribing rates at each clinic. Then all the village clinics will be randomised at a 1:1 ratio to usual care group vs intervention group, using the baseline antibiotic prescribing rates to balance high and low prescribing clinics between the arms. Each group will have 32 village clinics. (details see Fig. 1)

Recruitment of patients

All patients with RTIs who seek health care from the recruited clinics will be screened for eligibility and invited to participate in this study. Researchers from Anhui Medical University will undertake patient recruitment on-site until 30 participants have been recruited at each village clinic. Informed consent (Appendix 4, Supplementary material) will be obtained from all the patients who agree to participate in this study.

Currently, patients with suspected Covid-19 symptoms must be referred to specialist hospitals and village clinics are not expected to see suspected Covid-19 patients to present for treatment. However, some of these patients test negative for Covid-19 and are then referred back to their local clinic for treatment for their non-Covid RTI. These patients will be eligible for inclusion in our study.

Eligibility Criteria
Inclusion criteria:
1. Adult (aged ≥18 years old)
2. Patient who is diagnosed with a RTIs in the recruitment consultation: blocked/runny nose, coughing with or without sputum, dry/sore throat, breathing problems, fever, ear inflammation (blocked ear, Tinnitus, ear discharges, earache),

Exclusion criteria
1. Patients who have previously sought treatment for this illness from a normal clinic (not a specialist Covid clinic).

For peer review only - http://bmjopen.bmj.com/site/about/guidelines.xhtml
UOB Confidential & Sensitive

2. Patients who are unable to provide fully informed consent (eg dementia, psychosis, or severe depression)
3. Patients who are pregnant
4. Patients with immunological deficiencies
5. Patient with non-infective conditions (eg, pulmonary embolus, heart failure, oesophageal reflux, or serious allergy)

Randomisation, allocation & blinding

All the recruited village clinics will be stratified by baseline antibiotic prescribing rate quartile and geographical location (north, central, south) and then randomly allocated at a 1:1 ratio to the intervention or usual care group. All participating clinics will be allocated to a trial arm at the same time, after completion of baseline data collection.

Due to the nature of the intervention, it is not possible to blind village practitioners to their allocation to either intervention or usual care group. However, the statisticians conducting the analysis will remain blind to allocation until quantitative analysis is complete. The trial randomisation and blinding will be conducted by an independent statistician.

Intervention

The complex intervention comprises training, peer support, a computerised decision aid, a public commitment letter and a patient leaflet. These were co-designed with practitioners and patients using the person-based approach method for intervention development. [31]

Intervention practitioners will receive training delivered face to face in one session by a senior clinician from a tertiary hospital. The training covers: i) evidence about AMR in China and the link to their own practice; ii) addresses key concerns and misapprehensions; iii) details the current Chinese clinical guidelines on treatment of RTIs; iv) explains how to use the computer-based decision support aid; and v) outlines communication strategies to improve patient acceptance of a non-antibiotic treatment decision.

Peer support groups will be established to connect the village practitioners to others in the intervention group in order to provide peer support to each other during implementation. Peer support groups will meet via a widely used social media app in China (WeChat). At the end of the training session intervention practitioners will be asked to organise themselves into 3-4 sub-groups and identify a lead for each group. The group leader will be provided with a meeting topic guide and be responsible for setting up regular virtual meetings of the peer support group, during which each member of the group will share their experience of applying the intervention, discuss any problems and support each other to find solutions. Practitioners will be encouraged to contact each other outside of the scheduled meetings to ask for and provide support to each other.

A computer-based decision support system will be installed on computers in consulting rooms in the intervention clinics. This will allow village practitioners to select the patient's symptoms and diagnosis and will then provide the appropriate treatment guidance from the Chinese national guidelines.

Village practitioners will be required to sign a public declaration letter stating that they are committed to reducing unnecessary antibiotic prescribing and display this in a public area of their clinic.

Clinics will be provided with a leaflet that can be given to RTI patients. This leaflet will address common patient beliefs and concerns about RTI, address common misapprehensions that can lead to an expectation of antibiotic treatment, provides advice on non-antibiotic symptomatic treatment options and safety-netting.

For peer review only - http://bmjopen.bmj.com/site/about/guidelines.xhtml

UOB Confidential & Sensitive

Data collection methods

Baseline data will be collected for 2000 patients with RTI at the participating village clinics. Follow-up data collection will be collected for another 2000 patients at the participating village clinics after implementation of the intervention.

In all recruited village clinics, direct observations will be carried out by a researcher from Anhui Medical University following the doctor questionnaire (Appendix 5, supplementary material) adapted from our previous study.[20] Observation will focus on the adult RTI clinical consultations and record patient symptoms, practitioner's diagnosis & treatment, sickness severity, any antibiotics or other medicines prescribed and mode of administration (IV or oral), name, dosage and quantity. Immediately after the consultation, the researcher will fill in a patient questionnaire (Appendix 6, supplementary material) by asking questions of patients directly. This questionnaire is also adapted from the previous study [20] and includes questions about patient demographic characteristics (gender, age, education level, residential area and medical insurance records), illness duration, symptom severity and patient satisfaction with the consultation; the latter two questions will be scored using a 10-point scale.

The researcher will conduct telephone interviews with recruited RTI patients at 3, 7, 14, 21 days after their consultation. Structured questionnaires will be completed for each patient by the researcher during the telephone interview, with different secondary outcome data collected at different time points (see Table 1). Illness severity (scored on a 10-point scale) and symptom duration will be recorded at each time point. Patient attitude, understanding of the patient leaflet and beliefs (Appendix 7, supplementary material) will be recorded once at 3 days after consultation. Patient recovery situation (when they feel better & when they feel they could get back to normal) will be checked at 7 and 14 days after consultation (Appendix 8, Supplementary material). Patient consumption of antibiotics (including name, days, dosage and quantity) will be recorded at 21 days after consultation (Appendix 9, Supplementary material). Other healthcare use (clinic visits or consumption of other treatment) will be recorded at 21 days after consultation Patient out-of-pocket spending due to this illness, including purchase of medication, fees for clinic attendance, days off-work , will be recorded at 21 days after consultation. Patient quality of life will be measured using the EQ5D-5L at 3, 7, 14 & 21 days after consultation. (Use of paper interview administration mode of EQ5D-5L in Chinese version has been authorized by EuroQol Group (registration ID: 32880).

Data analysis

The primary analysis will use a generalised linear mixed model with a logit link and a random effect for clinic, allowing for the clustering of participants within clinics. The model will control for the clinic-level baseline antibiotic prescribing rate as well as potential confounders, and the full list of model covariates will be prespecified and set out in the statistical analysis plan. Results will be reported as odds ratios with 95% confidence intervals.

Missing data will be treated as missing at random and imputed using a chained-equations multiple imputation model. A complete cases analysis will be presented as a secondary analysis.

The analysis of secondary outcomes will use a similar modelling framework with distributions appropriate to the outcome, e.g. linear modelling for continuous outcomes and Poisson/negative binomial for count outcomes. These models will control for clustering and the factors listed above.

For peer review only

For peer review only - http://bmjopen.bmj.com/site/about/guidelines.xhtml

UOB Confidential & Sensitive

All results will be reported in line with the Cluster Randomised Trials extension to the CONSORT guidelines. No interim analyses are planned. Full details of the analyses to be undertaken will be set out in the Statistical Analysis Plan and approved by the Trial Steering Committee (TSC)

Sample size calculation

Our previous study recorded an antibiotic prescribing rate of 85.6% for rural areas in Anhui province [25] and a previous pilot intervention showed a reduction in antibiotic prescribing of more than 20% [32] There is no internationally agreed minimally important difference in antibiotic prescribing, however we anticipate that a reduction of at least 10% in antibiotic prescribing would represent a meaningful change at the population level given the very high rates of antibiotic prescribing.

To detect a possible absolute difference of 10% in prescribing rates between intervention (75%) and control practices (85%) with 90% power and alpha 0.05, we will need 335 patient consultations for infection RTI in each arm. Based on our previous study results the estimated ICC value was 0.05 and a design effect value would be 2.45. We would therefore aim to recruit at least 2.45*335*2=1642 patients into the study. Allowing for a drop-out rate of 15% and a cluster size of 30 patients per village clinics, we will recruit 64 village clinics into the study and each arm will have 32 village clinics.

Economic evaluation

The economic evaluation will be taken from a societal perspective and consider cost implications for patients as well as health services. Resource usage information will be costed using corresponding Chinese tariff.

All itemised resource usage data will be costed using appropriate data, with unit costs of visiting health care facilities based on medical service price items. Medication based on clinical consultation patient questionnaire and phone interviews with the patients at 3, 7, 14 and 21 days; and the time spent costed at average wage in Anhui Province. Insurance coverage will be weighted to calculate private spending.

Resource use will be weighted by its unit cost over the study period. Accumulated costs per patient will be calculated for the study period. Quality of life data will be translated into a utility score using a Chinese tariff. Quality adjusted life years will be estimated using the area under the curve approach.

Missing data will be handled by multiple imputation methods. Difference in costs and QALYs will be compared using a generalised linear mixed model taking clinic as cluster level and by adjusting baseline clinical and quality of life variables. Bootstrapping methods will be used to estimate the incremental cost per QALYs gained and per percentage reduction of antibiotic prescribing. Major assumptions will be explored through sensitivity analyses. Completed cases analyses will be presented as one of such scenario analyses.

Qualitative Process Evaluation

A Mandarin-speaking qualitative researcher will conduct observations of the training process, peer support discussions and intervention implementation. The key content related to participants during the training process, including any additions by the trainer, questions and answers, comments or discussion among participants and non-verbal reactions displayed by participants will be recorded in field notes. The researcher will be added as an observer into the WeChat groups to record the content of peer support discussions including participants' queries, examples of challenges and solutions and

For peer review only

UOB Confidential & Sensitive

any other discussion. The researcher will visit intervention clinics after follow-up data collection is completed and observe how the intervention is being implemented.

A Mandarin-speaking qualitative researcher will conduct semi-structured interviews with health professionals. Short interviews will be conducted at the end of the training, to elicit intervention practitioners' views about the training. Following completion of follow up date collection, intervention practitioners will be invited to participate in a semi-structured interview to understand their experiences of the intervention. They will be asked to describe how they used the intervention in their practice, any barriers and facilitators encountered in following the intervention and whether it influenced their views or practice.

A purposive sample of up to 40 patients who are recruited into the study will be interviewed. Patient will be selected to capture a range of socio-demographic characteristics and illness presentations. Some will be interviewed immediately after completing their consultation with health practitioners and some after they have completed the telephone questionnaires. Interviews will be semi-structured and guided by a topic guide that will cover experience of the consultation and understandings of the patient leaflet they received.

All semi-structured interviews will be transcribed. The qualitative data will be analysed thematically.

Data handling and repository

This trial will be monitored by a Trial Steering Committee and collected data will be monitored by data monitoring committee. There are no anticipated risks to patients from taking part in the study and participants have the right to withdraw from the study at any time without giving a reason.

Hard copies of documents with identifiable details of patient, practitioner and clinic will be stored in lockable cabinets on premises approved by the PI or relevant Co-Is at Anhui Medical University and any data created will be encrypted and stored on University secure servers until they can be uploaded or scanned and stored in the University of Bristol Research Data Storage Facility (RDSF) at the first available opportunity. Only authorised users can access data stored within the RDSF and procedures will be put in place to allow authenticated, external collaborators to view, add and/or edit data.

When possible, data will be exported directly to databases and consistency maintained via peer review and cross-checks with previous measurements. Manually inputted data will be double entered. Quality of questionnaire and interview data will be assured by training, provision of an interview manual and re-survey of 5% of subjects.

Patients and public involvement

Patients (and practitioners) contributed to the co-design of the intervention. They also provided feedback on piloted data collection forms, which led to modifications. The results will be disseminated to study participants and other stakeholders. At the end of the research project we will hold a high-level meeting to disseminate our findings to key stakeholders and develop a strategy for impact with relevant stakeholders.

ETHICS and dissemination

This project is one of the three work packages in the UK-China AMR Centre Partnerships 2018 (Strategies to reduce the burden of antibiotic resistance in China) supported by UKRI Medical Research Council (Grant Ref: MR/S013717/1)) and National Natural Science Foundation of China (Grant Ref:

For peer review only - http://bmjopen.bmj.com/site/about/guidelines.xhtml

81661138001). The protocol is rooted in a multidisciplinary collaboration between institutions in the UK and China.

Throughout the research project we will involve key stakeholders with roles in managing antibiotic use and antimicrobial stewardship. Research findings will be translated into short policy briefings with recommendations informed by existing policies in China and to influence current practice in health care settings. The credibility of research findings will be indicated to stakeholders through peer-reviewed publications in internationally recognised journals and presentations at academic conferences in China, the UK and internationally.

Acknowledgments

The authors acknowledge MRC & Newton Fund through a UK-China AMR Partnership Hub award (MR/S013717/1), and National Natural Science Foundation of China award (81861138049).

Author Contributions

HL, DW, CC, LY were responsible for developing the research questions and HL, DW, CC, LY, PL, BS, LG, IO were responsible for the study design. TZ, JC, JC, XS, RL, XH, YS are responsible for data collection and WC, JC & LZ are responsible for study management and coordination. WC, JC, BS, TT, GY and CC drafted the paper. All authors read, commented on and approved the final manuscript.

Competing interests statement

None declared

References

1. Tenover FC. Mechanisms of Antimicrobial Resistance in Bacteria. *Am J Med*. 2006;119(6, Supp1):S3-S10.
2. Antimicrobial resistance: 2014 global report on surveillance. Geneva: World Health Organization; 2014.
3. Holmes AH, Moore LSP, Sundsfjord A, et al. Understanding the mechanisms and drivers of antimicrobial resistance. *Lancet*. 2016;387(10014):176-187.
4. Levy SB, Marshall B. Antibacterial resistance worldwide: causes, challenges, and responses. *Nat Med*. 2004;10(12):S122-S129.
5. Laxminarayan R, Matsoso P, Pant S, et al. Access to effective antimicrobials: a worldwide challenge. *Lancet*. 2016;387(10014):168-175.
6. Morris DE, Cleary DW, Clarke SC. Secondary Bacterial Infections Associated with Influenza Pandemics. *Front Microbiol*. 2017;8:1041-1041.
7. Morens DM, Taubenberger JK, Fauci AS. Predominant Role of Bacterial Pneumonia as a Cause of Death in Pandemic Influenza: Implications for Pandemic Influenza Preparedness. *J Infect Dis*. 2008;198(7):962-970.
8. MacIntyre CR, Chughtai AA, Barnes M, et al. The role of pneumonia and secondary bacterial infection in fatal and serious outcomes of pandemic influenza a(H1N1)pdm09. *BMC Infect Dis*. 2018;18(1):637.
9. Zhou F, Yu T, Du R, et al. Clinical course and risk factors for mortality of adult inpatients with COVID-19 in Wuhan, China: a retrospective cohort study. *Lancet*. 2020;395(10229):1054-1062.
10. Mao W, Chen W. CHINA: The Zero Mark-up Policy for essential medicines at primary level facilities. Health Systems, Governance & Financing, World Health Organization; 2015.
11. Xiao Y, Li L. China's national plan to combat antimicrobial resistance. *Lancet Infect Dis*. 2016;16(11):1216-1218.

For peer review only

UOB Confidential & Sensitive

12. Xiao Y, Li L. Legislation of clinical antibiotic use in China. *Lancet Infect Dis*. 2013;13(3):189-191.
13. Yang L, Liu C, Ferrier JA, et al. The impact of the National Essential Medicines Policy on prescribing behaviours in primary care facilities in Hubei province of China. *Health Policy Plan*. 2013;28(7):750-760.
14. Wang J, Wang P, Wang X, et al. Use and Prescription of Antibiotics in Primary Health Care Settings in China. *JAMA Intern Med*. 2014;174(12):1914-1920.
15. Wei X, Zhang Z, Walley JD, et al. Effect of a training and educational intervention for physicians and caregivers on antibiotic prescribing for upper respiratory tract infections in children at primary care facilities in rural China: a cluster-randomised controlled trial. *Lancet Glob Health*. 2017;5(12):e1258-e1267.
16. Guan X, Tian Y, Song J, et al. Effect of physicians' knowledge on antibiotics rational use in China's county hospitals. *Soc Sci Med*. 2019;224:149-155.
17. Zhan Q, Wang YL, Chen X. Evaluation of antibacterial use in outpatients of township and community primary medical institutions in a district of Sichuan Province, China. *J Glob Antimicrob Resist*. 2019;19:201-206.
18. Wang CN, Huttner BD, Magrini Net al. Pediatric Antibiotic Prescribing in China According to the 2019 World Health Organization Access, Watch, and Reserve (AWaRe) Antibiotic Categories. *J Pediatr*. 2020;220:125-131.e5.
19. Lambert H, Chen M, Cabral C. Antimicrobial resistance, inflammatory responses: a comparative analysis of pathogenicities, knowledge hybrids and the semantics of antibiotic use. *Palgrave Commun*. 2019;5(1):85.
20. Zhao L, Kwiatkowska RM, Chai J, et al. Pathways to optimising antibiotic use in rural China: identifying key determinants in community and clinical settings, a mixed methods study protocol. *BMJ Open*. 2019;9(8):e027819.
21. Gulliford MC, Dregan A, Moore MV, et al. Continued high rates of antibiotic prescribing to adults with respiratory tract infection: survey of 568 UK general practices. *BMJ Open*. 2014;4(10):e006245.
22. Zumla A, Al-Tawfiq JA, Enne VI, et al. Rapid point of care diagnostic tests for viral and bacterial respiratory tract infections—needs, advances, and future prospects. *Lancet Infect Dis*. 2014;14(11):1123-1135.
23. Godman B, Haque M, McKimm J, et al. Ongoing strategies to improve the management of upper respiratory tract infections and reduce inappropriate antibiotic use particularly among lower and middle-income countries: findings and implications for the future. *Curr Med Res Opin*. 2020;36(2):301-327.
24. Wang CN, Huttner BD, Magrini N, et al. Pediatric Antibiotic Prescribing in China According to the 2019 World Health Organization Access, Watch, and Reserve (AWaRe) Antibiotic Categories. *J Pediatr*. 2020;220:125-31.e5.
25. Chai J, Coope C, Cheng J, et al. Cross-sectional study of the use of antimicrobials following common infections by rural residents in Anhui, China. *BMJ Open* 2019; 9:e024856.
26. Song J-H, Thamlikitkul V, Hsueh P-R. Clinical and economic burden of community-acquired pneumonia amongst adults in the Asia-Pacific region. *Int J Antimicrob Agents*. 2011;38(2):108-117.
27. Wang X, Xuan Z, Storella TH, et al. Determinants of non-prescription antibiotic dispensing in Chinese community pharmacies from socio-ecological and health system perspectives. *Soc Sci Med*. 2020:113035.
28. Chen C, Dong W, Shen JJ, et al. Is the prescribing behavior of Chinese physicians driven by financial incentives? *Soc Sci Med*. 2014;120:40-48.
29. Xiao Y, Shen P, Zheng B, et al. Change in Antibiotic Use in Secondary and Tertiary Hospitals Nationwide After a National Antimicrobial Stewardship Campaign Was Launched in China, 2011-2016: An Observational Study. *J Infect Dis*. 2020;221(2):S148-S155.
30. Xiao Y. Antimicrobial Stewardship in China: Systems, Actions and Future Strategies. *Clin Infect Dis*. 2018;67:S135-S141.
31. Yardley L, Morrison L, Bradbury K, et al. The person-based approach to intervention development: application to digital health-related behavior change interventions. *J Med Internet Res.* 2015;17(1):e30.
32. Shen X, Lu M, Feng R, et al. Web-Based Just-in-Time Information and Feedback on Antibiotic Use for Village Doctors in Rural Anhui, China: Randomized Controlled Trial. *J Med Internet Res*. 2018;20(2):e53.

For peer review only

UOB Confidential & Sensitive

For peer review only

Table 1 List of the primary and secondary outcome measures and when they will be collected both at baseline audit and follow-up audit.

Item	Outcome measure	Initial consultation	Patient Telephone interview after consultation			
			3 days	7 days	14 days	21 days
Primary outcome	Prescribing rate of antibiotics	√				
Secondary outcomes	Patient satisfaction	√				
	Patient symptom severity and duration		√	√	√	√
	Patient recovery			√	√	
	Patient consumption of antibiotics					√
	Patient attitude, beliefs and understanding of the patient leaflet		√			
	Patient costs					√
	Patient quality of life (EQ5D-5L)		√	√	√	√

UOB Confidential & Sensitive

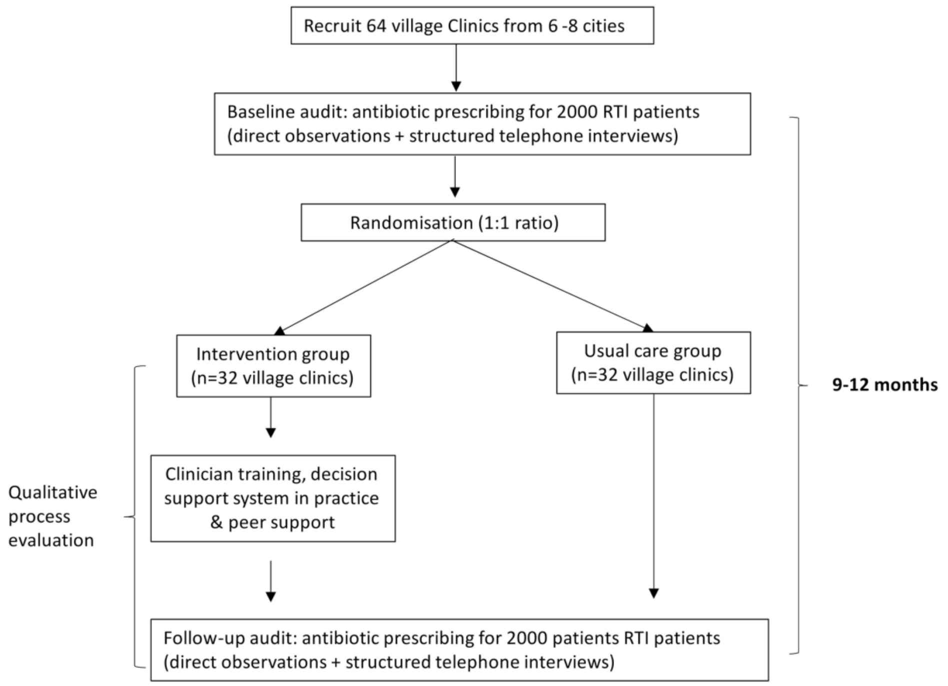

Trial flow chart

84x60mm (600 x 600 DPI)

Study Number:

Participated Clinic Identification Number for this trial:

CONSENT FORM

Title of Project: Design and evaluate a tailored intervention to modify antibiotic prescribing behaviour among health professionals and reduce antibiotic consumption: a cluster randomised controlled trial

Name of Researcher:

Please initial all boxes

1. I confirm that I have read and understand the information sheet dated [**DATE**] (version 1) for the above study. I have had the opportunity to consider the information, ask questions and have had these answered satisfactorily. ☐
2. I understand that my participation is voluntary and that I am free to withdraw at any time without giving any reason, without my medical care or legal rights being affected. ☐
3. I understand that any information I provide will be anonymised by removing all identifying details and will be kept strictly confidential and used only for research purposes. After the study, the anonymised information will be made publicly available for potential further research, but it will not be possible to identify me from the data. ☐
4. I understand that with my agreement, the interview may be audio-recorded but no one except the research team will hear this recording. I am aware that anonymised quotes from the interview may be included in reports of the study findings. ☐
5. I agree to take part in the above study. ☐

**Name of Participant**	**Date**	**Signature**
**Name of Person taking consent (if applicable)**	**Date**	**Signature**
**Name of Researcher**	**Date**	**Signature**

Consent form date of issue: **[DATE]**
Consent form version number: **[VERSION]**

For peer review only - http://bmjopen.bmj.com/site/about/guidelines.xhtml

Clinic background information questionnaire

村卫生室基本情况调查表

This questionnaire should be filled by the senior clinician in the village clinic.
这张问卷请由此村卫生室的负责医生填写。

名称：_______（市）_______县（区）________乡（镇）__________村
_______Village,_______township (town),_______county, _______city

一、村卫生室的基本情况

Basic information of village clinic

1、按照设置规划，本卫生室服务<br>1．1 覆盖地域面积为_______平方公里；<br>1．2 服务覆盖总人口_________人；<br>1．3 其中，常住人口________人；	1.The information about services of the village clinic<br>1.1 What is the service coverage area? ________square kilometers;<br>1.2 What is the total population covered by the service? ______;<br>1.3 What is the number of permanent residents covered by the service? ______;
2、目前，本村卫生室所服务的行政村，实际共有其他村级医疗机构______个，并请说明医疗机构名称_______	2. At present, the administrative villages served by the village clinic actually have ______other village level medical institutions, *[please specify their names]* __________
3、是否提供中医服务？（1）是 （2）否<br>3.1 包括哪些中医服务？<br>□草药；□针灸；□推拿；□其他______	3. Is TCM service provided here?<br>(1) Yes; (2) No<br>3.1 What TCM services are included?<br>□ herbal; □acupuncture; □ massage; □ other *[please specify]* _________
4、本村卫生室有医生数量_________个.	4. The village clinic has _____doctors.

二、村卫生室的医疗服务情况

Medical services in village clinics

2.过去一年接诊多少门诊病人？_________<br>2．1 其中，呼吸道感染的病人多少人？___	2. How many outpatients are there in the past year?<br>2.1 How many patients with respiratory infection?

For peer review only

Village practitioner background information questionnaire

村医基本情况调查表

This questionnaire should be filled by all clinicians working in this village clinic.
全部在此村卫生室工作的医生都需要填写此表。

Gender?<br>□Male<br>□Female	性别?<br>□男<br>□女
Date of birth? ___________<br>Age: _____years.	出生日期：_____年_____月_____日；<br>或年龄？______岁
Education?<br>□ middle school and below;<br>□ high school /polytechnic school / technical school;<br>□ junior college;<br>□ undergraduate;<br>□ postgraduate and above;<br>□ others, please specify_____	学历？<br>□初中及以下；<br>□高中/中专/技校；<br>□专科；<br>□大学本科；<br>□研究生及以上；<br>□其他，请说明_____
How many years of medical training have you received? _______years;	你受到多少年的医学专业训练？<br>_______年；
What kind of medical training/learning do you have? What is your specialty?<br>□ Clinical medicine;<br>□ TCM;<br>□ Nursing;<br>□ Others, please specify_______	所学专业？<br>□临床医学；<br>□中医学；<br>□护理学；<br>□其他，请说明_______
How long have you worked as a physician? _______years;	您从事医生的工作年限？ _______年；
How long have you worked here? _______years;	你在这里工作多久了？ _______年；
Type of certificate you obtained:<br>□ licensed physician;<br>□ Certified assistant physician;<br>□ Registered nurse;<br>□ Village doctor;<br>□ No certificate	你获得证书类别：<br>□执业医师；<br>□执业助理医师；<br>□注册护士；<br>□乡村医生；<br>□无证书
Category of doctor practice:<br>□ Clinical;<br>□ Chinese medicine;<br>□ Public health<br>□ Others, please specify_______	医师执业类别：<br>□临床；<br>□中医；<br>□公共卫生<br>□其他，请说明_______
Do you have any further study experience? How many months in total?;<br>□ Yes (if yes, total months? _______months) ;<br>□ No;	你有没有进修的经历？<br>□有（如果有，共_____个月？）；<br>□无；
Have you ever participated in the training on the use of antibiotics? □Yes;□No;<br>If yes, how many times? _______times;<br>How many months in total? _______months.	有没有参加过关于抗菌药物使用的培训？<br>如果有，几次？<br>累计多少个月？

For peer review only

For peer review only

Study Number:

Patient Identification Number for this trial:

CONSENT FORM

Title of Study: Appropriate use of antibiotics for RTI in primary care: a cluster randomised controlled trial

Name of Researcher: XXX (contact details)

	**Please initial all boxes**
I confirm that I have read information sheet /went through information sheet with the researcher. I understand it and has had the opportunity to ask questions.	
I understand that my participation is voluntary and that I am free to withdraw at any time without giving any reason, without my medical care being affected.	
I understand that all the information is confidential. No name or personal information will be used when the results of this study is published.	
I agree to take part in the above study.	

Name of Participant **Date** **Signature**

Name of Person taking consent

(if applicable) **Date** **Signature**

Name of Researcher **Date** **Signature**

**Participant contact details (used for telephone questionnaire)**	
Phone number	
WeChat ID	

Consent form date of issue: **[DATE]**

Consent form version number: **[VERSION]**

For peer review only

For peer review only - http://bmjopen.bmj.com/site/about/guidelines.xhtml

Participant ID: ___________________

Clinical consultation doctor questionnaire

These questions must be answered by the **village practitioner** or by direct observation by the field worker

这部分问题要求**医生**回答或者通过调查员的观察完成

1: What are the patient's symptoms? 病人当前有何症状?

☐ Blocked/ runny nose	☐ 鼻塞/流涕
☐ Blocked nose<br>☐ Runny nose (clear/watery discharge)<br>☐ Snotty nose (yellow/green discharge)	☐ 鼻子不通<br>☐ 流清水鼻涕<br>☐ 流脓鼻涕 (黄/绿色鼻涕)
☐ Coughing	☐ 咳嗽
☐ Dry cough<br>☐ Cough with white sputum<br>☐ Cough with yellow/green sputum	☐ 干咳<br>☐ 咳嗽带白色痰<br>☐ 咳嗽带黄/绿色脓痰
☐ Throat problems	☐ 嗓子不舒服
☐ Sore throat<br>☐ Hyperemia/ swelling in throat<br>☐ Hyperemia/ enlargement of tonsils<br>☐ Pus on the tonsils<br>☐ Swollen lymph glands	☐ 咽痛<br>☐ 咽喉部充血/水肿<br>☐ 扁桃体充血/肿大<br>☐ 扁桃体化脓<br>☐ 下颌淋巴结肿大
☐ Breathing	☐ 呼吸
☐ Short of breath<br>☐ Tight chest<br>☐ Difficulty breathing<br>☐ Must sit erect to breathe<br>☐ Wet crackling in lungs	☐ 气促<br>☐ 胸闷<br>☐ 呼吸不畅/困难<br>☐ 端坐呼吸<br>☐ 肺部湿罗音
☐ Ear symptoms	☐ 耳部症状
☐ Blocked ears<br>☐ Tinnitus<br>☐ Pus/ fluid secretion<br>☐ Earache<br>☐ Loss of hearing	☐ 耳闷<br>☐ 耳鸣<br>☐ 流脓/液<br>☐ 耳痛<br>☐ 听力下降
☐ Fever	☐ 发热
☐ Low 37.3-38 ºC<br>☐ Moderate 38.1-39 ºC<br>☐ High >39 ºC	☐ 低热 37.3-38 摄氏度<br>☐ 中等度热 38.1-39 摄氏度<br>☐ 高热 39 摄氏度以上
☐ Pain	☐ 疼痛

For peer review only

Participant ID: ____________________

☐ Headache<br>☐ Aching all-over<br>☐ Chest pain	☐ 头痛<br>☐ 全身酸痛<br>☐ 胸痛

2. Diagnosis & Treatment 诊断和治疗

Tick one 选一种	**2.1 What is the diagnosis? (ask doctor to pick closest category from list of 6 RTI)**	**2.1 诊断是什么？（请医生在六类呼吸系统感染中选择最接近诊断结果的一项）**
	1. Acute upper respiratory tract infection including:<br>. Infectious cold / Acute rhinitis / upper respiratory tract catarrh<br>. Acute inflammation of the throat<br>. Acute viral pharyngitis<br>. Acute viral laryngitis	急性上呼吸道感染:<br>· 传染性感冒/急性鼻炎/上呼吸道粘膜炎<br>· 喉咙急性炎症<br>· 急性病毒性咽炎<br>· 急性病毒性喉炎
	2. Acute tonsil inflammation / Tonsillitis / Pharyngitis	急性化脓性扁桃体炎/ 扁桃体炎/咽炎
	3. Acute ear inflammation / Otitis Media	急性耳朵炎症/中耳炎
	4. Acute sinusitis	急性鼻窦炎
	5. Acute tracheal/ bronchial inflammation / Acute Tracheobronchitis / Bronchitis / Lower respiratory tract infection	急性气管/支气管炎症/急性气管支气管炎/支气管炎/下呼吸道感染
	6. Pneumonia	肺炎
	. Other please state [free text box]	其他请说明[自由文本框]

**2.2 One a scale of 0 to 10, how sick do you think this patient is? (where 10 is very sick and 0 is not sick)**	**2.2 在 0 到 10 的程度中，你认为这位病人有多不舒服？(10 表示非常不舒服， 0 表示没有不舒服)**
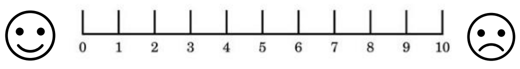	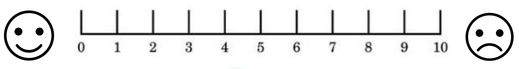

**2.3 Were any antibiotics prescribed?**	**2.3 抗生素是否开具？**
☐ Yes<br>☐ No	☐ 是<br>☐ 否
**If Yes, first tick the method of drug administration; then specify** the name, total quantity and dosage in the table<br><br>☐ IV Antibiotics prescribed<br>☐ Oral antibiotics prescribed	**如果是，请先勾选抗生素的给药方式；再在下表中具体说明**药物通用名，总量和剂量<br><br>☐ 静脉注射抗生素<br>☐ 口服抗生素

Participant ID: ________________

IV Antibiotics prescribed:

Tick if prescribed	Medicine Name	Specification	Total quantity	Dosage
	Penicillin			
	Levofloxacin			
	Ampicillin			
	Clindamycin			
	Amikacin			
	Azithromycin			
	Metronidazole			
	Fosfomycin sodium			
	Amoxicillin			
	Erythromycin			
	Norfloxacin			
	Cefradine			

静脉注射抗生素：

如果有，请勾选	药物名称	规格	总量	剂量
	青霉素			
	左氧氟沙星			
	氨苄青霉素			
	克林霉素			
	阿米卡星(丁胺卡那霉素)			
	阿奇霉素			
	甲硝唑			
	磷霉素钠			
	阿莫西林			
	红霉素			
	诺氟沙星			
	头孢拉定			

Oral antibiotics prescribed:

Tick if prescribed	Antibiotics Name	Specification	Total quantity	Dosage

For peer review only

Participant ID: ___________________

	Amoxicillin			
	Erythromycin			
	Norfloxacin			
	Cefradine			

口服抗生素：

如果有，请勾选	药物名称	规格	总量	剂量
	阿莫西林			
	红霉素			
	诺氟沙星			
	头孢拉定			

Other medicines prescribed:

**Tick if prescribed**	**Medicine Name**	**Specification**	**Total quantity**	**Dosage**	**Mode of administration**
	Dexamethasone				
	Tetracaine				
	chlorpheniramine				
	Aminophylline				
	Ribavirin				
	Compound paracetamol amine capsules				
	Ambroxol				
	Asthma-calming and cough-stopping patch (*pingchuan zhike pian*)				
	Qing kailing(qinghai ling)				
	Galculus Bovis and Metronidazole Capsules (*rengong niuhuang jia xiaocuo jiaonang*:				

其它开具的药物：

如果有，请勾选	药物名称	规格	总数量	剂量	服药方式（吊水/口服/贴等）
	地塞米松				
	丁卡因				

For peer review only

Participant ID: ___________________

	扑尔敏				
	氨茶碱				
	利巴韦林				
	复方氨酚胶囊				
	盐酸氨溴索				
	平喘止咳贴片				
	清开灵				
	人工牛黄甲硝唑胶囊				

**2.4 Did the patient ask for any of these treatments?**	**2.4 患者是否主动要求过以下治疗?**
☐ IV	☐ 输液
☐ Anti-inflammation medicine/antibiotics	☐ 抗生素/消炎药
☐ Other medicines	☐ 其他药物

Any further observation note of the consultation process (Researcher's observation):	其它有关诊疗咨询过程的观察记录?

For peer review only

Participant ID: _______________

WP1 Clinical consultation patient questionnaire

A field worker will administer this questionnaire at the clinical consultation at which the patient is recruited at both base line and follow up audit.

These questions must be answered by the **patient** or by direct observation by the field worker

这部分问题要求**病人**回答或者通过调查员的观察完成

1. Eligibility criteria 入选条件

Does patient have a suspected RTI?<br>. Yes<br>. No – exclude from study	病人是否疑似呼吸系统感染?<br>· 是<br>· 否—从本研究中排除
Is patient aged 18 years or older?<br>. Yes<br>. No – exclude from study	病人是否年满 18 岁?<br>· 是<br>· 否—从本研究中排除
Is today the first time the patient has sought treatment for this this illness or a repeat visit?<br>. First time consultation<br>. Repeat consultation – exclude from study	今天是病人首次还是再次为这个病来这里就诊?<br>· 首次就诊<br>· 再次就诊—从本研究中排除

Date completed: _________	调查日期: _____年___月___日
Name of field worker completing proforma: _________	调查员姓名: ________________

Participant ID: ________________

2. Participant Characteristics 研究对象基本情况

**2.1 Gender**<br>☐ Female<br>☐ Male	**2.1 性别:**<br>☐ 女性<br>☐ 男性
**2.2 Date of Birth (Year/Month/Day)**	**2.2 生日**<br>_____年___月___日
**2.3 How many years of education have you completed?**<br>_______________years<br>*[Write down the exact number]*	**2.3 你上了几年学?**<br>_____年<br>(写下确切的数字)

**2.4 Where do you live?**<br>*Name of town or village:*<br>____________________________________	**2.4 你住在哪里?**<br>乡镇/村的名字<br>___________________________
**2.5 If you have lived outside your usual home residence for more than a month over the past 12 months, how many months have you been away?**<br>________months	**2.5 在过去一年，如果你离开家在外生活超过 1 个月，请给出你具体离家几个月?**<br>_______个月
**2.6 Is the place where you have been living urban or rural?**<br>☐ Urban<br>☐ Rural<br>☐ Not applicable	**2.7 你在外生活的地方是农村还是城市**<br>☐ 城市<br>☐ 农村<br>☐ 不适用
**2.7 What is your Household registration status** (*hukou*) **for accessing health care benefits?**<br>☐ Urban<br>☐ Rural<br>☐ Other___________________	**2.8 目前跟你医保挂钩的是什么户口?**<br>☐ 城市<br>☐ 农村<br>☐ 其他___________________
**2.8 What type of insurance do you have, if any?**<br>☐ New rural cooperative Medical insurance scheme<br>☐ Urban Employee's Medical Insurance<br>☐ Urban Residents' Medical Insurance<br>☐ Not Having Medical Insurance<br>☐ Other Type of Insurance *[please specify]*:<br>_________________________	**2.9 你参加了哪些医疗保险?**<br>☐ 新型农村合作医疗<br>☐ 城镇职工医疗保险<br>☐ 城镇居民医疗保险<br>☐ 没有医疗保险<br>☐ 其他医疗保险（请注明）：<br>______________________

Commented [CC1]: Ask Jing Chai to check and amend

For peer review only

Participant ID: ________________

3. Duration of illness and the severity 患病时长和严重程度

Duration of your illness: _____ days	你的病已经持续了_______天
One a scale of 0 to 10, how sick do you feel? (where 10 is very sick and 0 is not sick)	在 0 到 10 的程度中，你认为你有多不舒服？（10 表示非常不舒服， 0 表示没有不舒服）
☺ 0 1 2 3 4 5 6 7 8 9 10 ☹	☺ 0 1 2 3 4 5 6 7 8 9 10 ☹

4. Resource usage

Have you had any treatment for this illness before coming here?	Bought from pharmacy	Obtained from other source (home, family, friends)	How much did you pay in total?	How much did you pay from your own pocket?
**Anti-bacterial/anti-inflammation medicine**				
☐ Amoxicillin	Y/N	Y/N	¥	¥
☐ Erythromycin	Y/N	Y/N	¥	¥
☐ Cefradine	Y/N	Y/N	¥	¥
☐ Levofloxacin	Y/N	Y/N	¥	¥
☐ Cefuroxime	Y/N	Y/N	¥	¥
☐ Other anti-bacterial/anti-inflammation medicine [please specify]	Y/N	Y/N	¥	¥
**Other medicines/treatments**				
☐ Paracetamol	Y/N	Y/N	¥	¥
☐ Ganmaoling Granules （e.g. 999 cold remedy）	Y/N	Y/N	¥	¥
☐ Qingkailing	Y/N	Y/N	¥	¥
☐ Yinhuang Granules	Y/N	Y/N	¥	¥
☐ Xiongju Shangqing	Y/N	Y/N	¥	¥
☐ Lianhua (lotus) Qingwen capsules	Y/N	Y/N	¥	¥
☐ Cough syrup	Y/N	Y/N	¥	¥
☐ Ambroxol Hydrochloride oral suspension	Y/N	Y/N	¥	¥
☐ Keteling	Y/N	Y/N	¥	¥
☐ Compound liquorice tablets	Y/N	Y/N	¥	¥
☐ Vitamin C	Y/N	Y/N	¥	¥

Commented [TZ2]: Nicola suggested to change the wording to ask how many medicines they have used and if they can recall which medicine they were and how much did they pay?
E.g. Q4. Have you had any treatment for this illness before coming here?
How many medicines have you used?
Please list the medicines (see table below) you used and How much you paid if known (in total/from your own pocket)?

This would at least allow us to estimate costs based on the number of medicines taken making an assumption on the average cost per medicine.
Lily agreed with Nicola and suggested we should give list name of medication (Most used). Based on past experiences, open questions in working never worked.

For peer review only

Participant ID: ________________

☐ Crystal sugar snow pear	Y/N	Y/N	¥	¥
☐ Ginger	Y/N	Y/N	¥	¥
☐ Mugwort	Y/N	Y/N	¥	¥
☐ Pipa (loquat)	Y/N	Y/N	¥	¥
☐ Orange peel granules	Y/N	Y/N	¥	¥
☐ Other [please specify]	Y/N	Y/N	¥	¥

在来这看病前，你是否已经自己用过药了？	从药店购买	其他方法获得（家中储存，家人，朋友）	这些药总共多少钱？	你自费付了多少钱？
**抗菌药/消炎药**				
☐ 阿莫西林	是/否	是/否	¥	¥
☐ 红霉素	是/否	是/否	¥	¥
☐ 头孢拉定	是/否	是/否	¥	¥
☐ 左氧氟沙星	是/否	是/否	¥	¥
☐ 头孢呋辛	是/否	是/否	¥	¥
☐ 其他抗菌药/消炎药（请注明）	是/否	是/否	¥	¥
**其他药物**				
☐ 扑热息痛	是/否	是/否	¥	¥
☐ 感冒灵颗粒（如 999 感冒灵）	是/否	是/否	¥	¥
☐ 清开灵	是/否	是/否	¥	¥
☐ 银黄颗粒	是/否	是/否	¥	¥
☐ 芎菊上清	是/否	是/否	¥	¥
☐ 莲花清瘟胶囊	是/否	是/否	¥	¥
☐ 咳嗽糖浆	是/否	是/否	¥	¥
☐ 盐酸氨溴索口服液	是/否	是/否	¥	¥
☐ 咳特灵	是/否	是/否	¥	¥
☐ 复方甘草片	是/否	是/否	¥	¥
☐ 维生素 C	是/否	是/否	¥	¥
☐ 冰糖雪梨	是/否	是/否	¥	¥
☐ 姜	是/否	是/否	¥	¥
☐ 艾草	是/否	是/否	¥	¥
☐ 琵琶	是/否	是/否	¥	¥
☐ 陈皮颗粒	是/否	是/否	¥	¥
☐ 其他（请注明）	是/否	是/否	¥	¥

**If you have used "anti-inflammation medicine/antibiotics", how many courses have you used in the past 6 months?**	**如果你已经使用了"消炎药/抗生素"，在过去 6 个月中你使用了几次？**

Commented [TZ3]: 'If you have used anti-inflammation medicine' means 'if you have used anti-inflammation for this illness' or 'if you have used anti-inflammation in the past 6 months'?

Does the number of courses include the use of anti-inflammation for this illness before visiting a doctor?

Commented [C4R3]: Health Economics team – please clarify what you need to know

For peer review only

Participant ID: ________________

☐ 0 times<br>☐ 1-3 times<br>☐ 4-6 times<br>☐ > 6 times	☐ 0 次<br>☐ 1-3 次<br>☐ 4-6 次<br>☐ > 6 次

**If you work, how many days have you taken off work due to this illness?**	如果你工作，那么你因为这次生病已经几天不去上班了？
{number} days	（ ）天
**If you are in full time education, please state how many days have you missed your education**	如果你是学生，那么你因为这次生病已经几天不去上学？
{number} days	（ ）天

5. Patient Satisfaction 患者满意度

**One a scale of 0 to 10, how satisfied were you with your visit to the doctor today? (where 10 is very satisfied and 0 is not satisfied at all)**	**在 0 到 10 的程度中，你今天在这里看病满意吗？(10 表示非常满意， 0 表示完全不满意)**
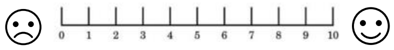	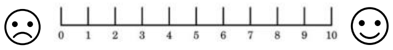

For peer review only

Patient telephone questionnaire – Day 3

A field worker will conduct phone interviews with the patients at 3, 7, 14 and 21 days. Patients will also be asked the questions from the EQ5D.

调查员将在第 3， 7， 14 和 21 天对病人进行电话访谈。除了以下问题，病人还会被问道一些 EQ5D 问卷中的问题。

1.Basic information 基本情况

**Patient ID**	**病人编号**
Days since baseline consultation<br>☐ 3 days<br>☐ 7 days<br>☐ 14 days<br>☐ 21 days	这是最初看病之后的第几天?<br>☐ 3 天<br>☐ 7 天<br>☐ 14 天<br>☐ 21 天
Date completed: _________	调查日期: _____年___月___日
Name of field worker completing proforma:<br>_________	调查员姓名: ____________________

2. Continuing Illness Severity 疾病持续严重程度

**One a scale of 0 to 10, how sick do you feel? (where 10 is very sick and 0 is not sick)**	**在 0 到 10 的程度中，你认为你有多不舒服？（10 表示非常不舒服， 0 表示没有不舒服）**
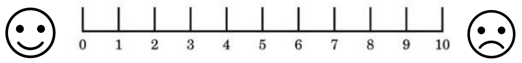	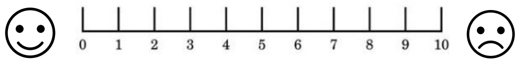

3. Patient view of leaflet 病人对宣传手册的看法

Did you have a look of the patient leaflet?<br>☐ Yes<br>☐ No<br>☐ Did not get leaflet	你是否看了病人宣传手册?<br>☐ 是<br>☐ 否<br>☐ 没有拿到
How easy was the patient leaflet to understand?<br>☐ Very easy, I understood all of it<br>☐ Easy, I understood most of it<br>☐ Not Easy, I only undersood a little<br>☐ Too hard, I did not understand any<br>☐ I am not able to read	你觉得病人宣传手册有多容易理解?<br>☐ 非常容易，我可以全部理解<br>☐ 容易，我可以理解大部分<br>☐ 不容易，我只能理解小部分<br>☐ 非常难，我完全不能理解<br>☐ 我读不了宣传手册

[Type here]

For peer review only

Did the leaflet change your ideas or beliefs about what illnesses need anti-inflammation medicine/ antibiotics?<br>☐ Yes<br>☐ No	病人宣传手册对您关于什么样的<br>疾病需要使用消炎药/抗生素的想法有改变吗?<br>☐ 有<br>☐ 没有

For peer review only

For peer review only - http://bmjopen.bmj.com/site/about/guidelines.xhtml

EQ-5D-5L

Health Questionnaire

English version for the UK

VERSION FOR INTERVIEWER ADMINISTRATION

Note to interviewer: although allowance should be made for the interviewer's particular style of speaking, the wording of the questionnaire instructions should be followed as closely as possible. In the case of the EQ-5D-5L descriptive system on page 2 of the questionnaire, the precise wording must be followed.

If the respondent has difficulty choosing a response, or asks for clarification, the interviewer should repeat the question word for word and ask the respondent to answer in a way that most closely resembles his or her thoughts about his or her health today.

INTRODUCTION

(Note to interviewer: please read the following to the respondent.)

We are trying to find out what you think about your health. I will explain what to do as I go along, but please interrupt me if you do not understand something or if things are not clear to you. There are no right or wrong answers. We are interested only in your personal view.

First, I am going to read out some questions. Each question has a choice of five answers. Please tell me which answer best describes your health TODAY.

Do not choose more than one answer in each group of questions.

(Note to interviewer: first read all five options for each question. Then ask the respondent to choose which one applies to him/herself. Repeat the question and options if necessary. Mark the appropriate box under each heading. You may need to remind the respondent regularly that the timeframe is TODAY.)

For peer review only - http://bmjopen.bmj.com/site/about/guidelines.xhtml

EQ-5D DESCRIPTIVE SYSTEM

MOBILITY

First, I would like to ask you about mobility. Would you say that:

1. You have no problems in walking about? ❑
2. You have slight problems in walking about? ❑
3. You have moderate problems in walking about? ❑
4. You have severe problems in walking about? ❑
5. You are unable to walk about? ❑

SELF-CARE

Next, I would like to ask you about self-care. Would you say that:

1. You have no problems washing or dressing yourself? ❑
2. You have slight problems washing or dressing yourself? ❑
3. You have moderate problems washing or dressing yourself? ❑
4. You have severe problems washing or dressing yourself? ❑
5. You are unable to wash or dress yourself? ❑

USUAL ACTIVITIES

Next, I would like to ask you about usual activities, for example work, study, housework, family or leisure activities. Would you say that:

1. You have no problems doing your usual activities? ❑
2. You have slight problems doing your usual activities? ❑
3. You have moderate problems doing your usual activities? ❑
4. You have severe problems doing your usual activities? ❑
5. You are unable to do your usual activities? ❑

PAIN / DISCOMFORT

Next, I would like to ask you about pain or discomfort. Would you say that:

1. You have no pain or discomfort? ❑
2. You have slight pain or discomfort? ❑
3. You have moderate pain or discomfort? ❑
4. You have severe pain or discomfort? ❑
5. You have extreme pain or discomfort? ❑

ANXIETY / DEPRESSION

Finally, I would like to ask you about anxiety or depression. Would you say that:

1. You are not anxious or depressed? ❑
2. You are slightly anxious or depressed? ❑
3. You are moderately anxious or depressed? ❑
4. You are severely anxious or depressed? ❑
5. You are extremely anxious or depressed? ❑

For peer review only

For peer review only - http://bmjopen.bmj.com/site/about/guidelines.xhtml

EQ-5D VAS

- **Now, I would like to ask you to say how good or bad your health is TODAY.**
- **I would like you to try to picture in your mind a scale that looks like a thermometer.**
  (Note to interviewer: if interviewing face-to-face, please show the person the VAS scale.)
- **The best health you can imagine is marked 100 (one hundred) at the top of the scale and the worst health you can imagine is marked 0 (zero) at the bottom.**
- **I would now like you to tell me the point on this scale where you would put your health TODAY.**
  (Note to interviewer: mark the scale at the point indicating the respondent's 'health today'. Now, please write the number you marked on the scale in the box below.)

THE RESPONDENT'S HEALTH TODAY = ☐

Thank you for taking the time to answer these questions.

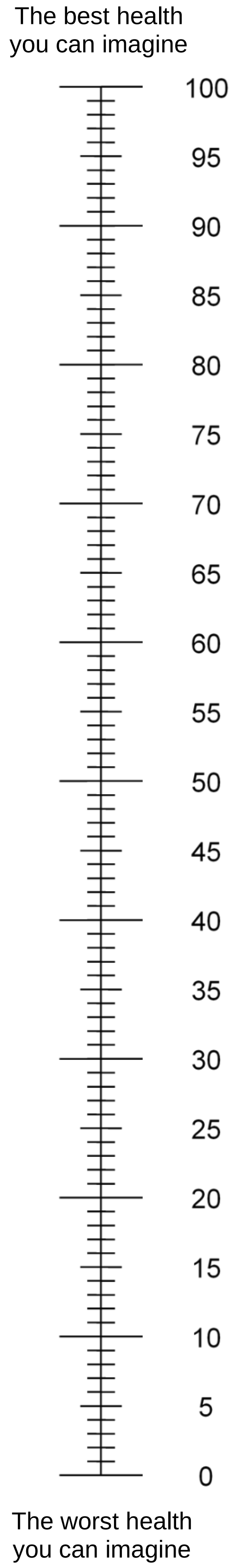

For peer review only

For peer review only - http://bmjopen.bmj.com/site/about/guidelines.xhtml

Patient telephone questionnaire – Day 7 & Day 14

A field worker will conduct phone interviews with the patients at 3, 7, 14 and 21 days. Patients will also be asked the questions from the EQ5D.

调查员将在第 3， 7， 14 和 21 天对病人进行电话访谈。除了以下问题，病人还会被问道一些 EQ5D 问卷中的问题。

1.Basic information 基本情况

**Patient ID**	**病人编号**
Days since baseline consultation<br>☐ 3 days<br>☐ 7 days<br>☐ 14 days<br>☐ 21 days	这是最初看病之后的第几天?<br>☐ 3 天<br>☐ 7 天<br>☐ 14 天<br>☐ 21 天
Date completed: __________	调查日期: ______年____月____日
Name of field worker completing proforma:<br>__________	调查员姓名: ____________________

2. Continuing Illness Severity 疾病持续严重程度

**One a scale of 0 to 10, how sick do you feel? (where 10 is very sick and 0 is not sick)**	**在 0 到 10 的程度中，你认为你有多不舒服？(10 表示非常不舒服， 0 表示没有不舒服)**
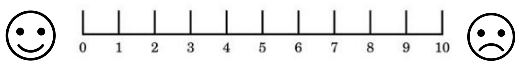	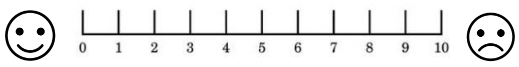

3. Recovery situation 康复情况

Do you recover from the illness?<br>☐ Yes<br>☐ No<br>☐ Not applicable	您是否已经康复了?<br>☐ 是<br>☐ 否<br>☐ 不确定
If yes, when did you recover?<br>Date:	如果您已经康复了，请问您是什么时候康复的?<br>日期:

For peer review only - http://bmjopen.bmj.com/site/about/guidelines.xhtml

EQ-5D-5L

Health Questionnaire

English version for the UK

VERSION FOR INTERVIEWER ADMINISTRATION

Note to interviewer: although allowance should be made for the interviewer's particular style of speaking, the wording of the questionnaire instructions should be followed as closely as possible. In the case of the EQ-5D-5L descriptive system on page 2 of the questionnaire, the precise wording must be followed.

If the respondent has difficulty choosing a response, or asks for clarification, the interviewer should repeat the question word for word and ask the respondent to answer in a way that most closely resembles his or her thoughts about his or her health today.

INTRODUCTION

(Note to interviewer: please read the following to the respondent.)

We are trying to find out what you think about your health. I will explain what to do as I go along, but please interrupt me if you do not understand something or if things are not clear to you. There are no right or wrong answers. We are interested only in your personal view.

First, I am going to read out some questions. Each question has a choice of five answers. Please tell me which answer best describes your health TODAY.

Do not choose more than one answer in each group of questions.

(Note to interviewer: first read all five options for each question. Then ask the respondent to choose which one applies to him/herself. Repeat the question and options if necessary. Mark the appropriate box under each heading. You may need to remind the respondent regularly that the timeframe is TODAY.)

For peer review only - http://bmjopen.bmj.com/site/about/guidelines.xhtml

EQ-5D DESCRIPTIVE SYSTEM

MOBILITY

First, I would like to ask you about mobility. Would you say that:

1. You have no problems in walking about? ❑
2. You have slight problems in walking about? ❑
3. You have moderate problems in walking about? ❑
4. You have severe problems in walking about? ❑
5. You are unable to walk about? ❑

SELF-CARE

Next, I would like to ask you about self-care. Would you say that:

1. You have no problems washing or dressing yourself? ❑
2. You have slight problems washing or dressing yourself? ❑
3. You have moderate problems washing or dressing yourself? ❑
4. You have severe problems washing or dressing yourself? ❑
5. You are unable to wash or dress yourself? ❑

USUAL ACTIVITIES

Next, I would like to ask you about usual activities, for example work, study, housework, family or leisure activities. Would you say that:

1. You have no problems doing your usual activities? ❑
2. You have slight problems doing your usual activities? ❑
3. You have moderate problems doing your usual activities? ❑
4. You have severe problems doing your usual activities? ❑
5. You are unable to do your usual activities? ❑

PAIN / DISCOMFORT

Next, I would like to ask you about pain or discomfort. Would you say that:

1. You have no pain or discomfort? ❑
2. You have slight pain or discomfort? ❑
3. You have moderate pain or discomfort? ❑
4. You have severe pain or discomfort? ❑
5. You have extreme pain or discomfort? ❑

ANXIETY / DEPRESSION

Finally, I would like to ask you about anxiety or depression. Would you say that:

1. You are not anxious or depressed? ❑
2. You are slightly anxious or depressed? ❑
3. You are moderately anxious or depressed? ❑
4. You are severely anxious or depressed? ❑
5. You are extremely anxious or depressed? ❑

For peer review only

For peer review only - http://bmjopen.bmj.com/site/about/guidelines.xhtml

EQ-5D VAS

- **Now, I would like to ask you to say how good or bad your health is TODAY.**
- **I would like you to try to picture in your mind a scale that looks like a thermometer.**
  (Note to interviewer: if interviewing face-to-face, please show the person the VAS scale.)
- **The best health you can imagine is marked 100 (one hundred) at the top of the scale and the worst health you can imagine is marked 0 (zero) at the bottom.**
- **I would now like you to tell me the point on this scale where you would put your health TODAY.**
  (Note to interviewer: mark the scale at the point indicating the respondent's 'health today'. Now, please write the number you marked on the scale in the box below.)

THE RESPONDENT'S HEALTH TODAY = [ ]

Thank you for taking the time to answer these questions.

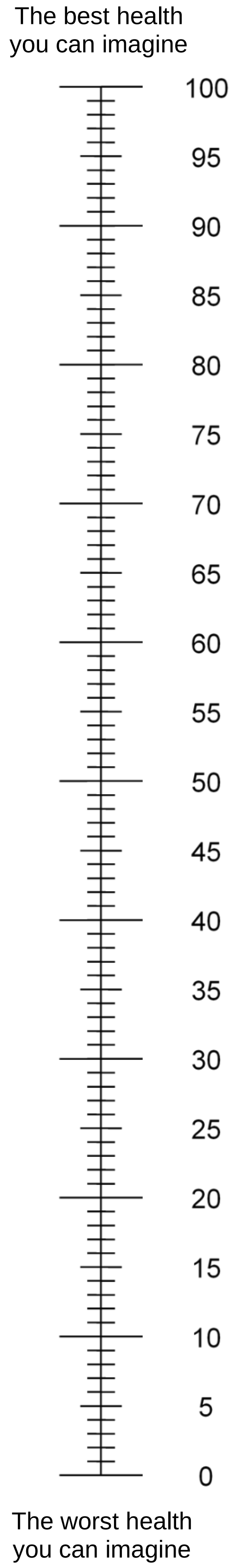

For peer review only

For peer review only - http://bmjopen.bmj.com/site/about/guidelines.xhtml

Patient telephone questionnaire – Day 21

A field worker will conduct phone interviews with the patients at 3, 7, 14 and 21 days. Patients will also be asked the questions from the EQ5D.

调查员将在第 3， 7， 14 和 21 天对病人进行电话访谈。除了以下问题，病人还会被问道一些 EQ5D 问卷中的问题。

1.Basic information 基本情况

**Patient ID**	**病人编号**
Days since baseline consultation<br>☐ 3 days<br>☐ 7 days<br>☐ 14 days<br>☐ 21 days	这是最初看病之后的第几天？<br>☐ 3 天<br>☐ 7 天<br>☐ 14 天<br>☐ 21 天
Date completed: __________	调查日期： ______年____月____日
Name of field worker completing proforma:<br>__________	调查员姓名： ______________________

2. Continuing Illness Severity 疾病持续严重程度

**One a scale of 0 to 10, how sick do you feel? (where 10 is very sick and 0 is not sick)**	**在 0 到 10 的程度中，你认为你有多不舒服？（10 表示非常不舒服， 0 表示没有不舒服）**
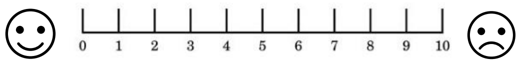	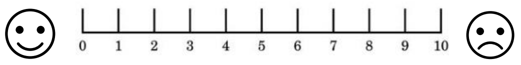

3. Antibiotic Consumption 抗生素服用情况

Were you prescribed oral anti-bacterial/anti-inflammation medicine in your baseline consultation?<br>☐ Yes (if yes, field worker will ask the two questions below in this table)<br>☐ No<br>☐ Not applicable	您在最初看病时是否有开口服抗菌药物/消炎药吗？<br>☐ 是（如果有，请继续问下面两个问题）<br>☐ 否<br>☐ 不清楚
How many days and times per day did you actually take a dose since you saw the Dr?<br>☐ [Number] _________of days<br>☐ [Number]_________ times / day	从那之后你已经吃了多少天？每天实际吃多少次？<br>☐ （ ） 天<br>☐ （ ） 次/天

For peer review only - http://bmjopen.bmj.com/site/about/guidelines.xhtml

What was the dose did you take every time?	您每次吃的剂量是多少?
Have you stopped taking the anti-bacterial/anti-inflammation medicine that you were prescribed?<br>☐ Yes<br>☐ No<br>☐ Not applicable	你是否已经停止服用给你开的抗菌药物/消炎药了?<br>☐ 是<br>☐ 否<br>☐ 不清楚

4. Resource Use for Economic Evaluation

Additional Health Care Utilisation 其他卫生服务的使用

Did you seek treatment for your illness from any of these since we talked to you in your consultation?	How many times did you visit there?	How much did you pay in total?	How much did you pay from your own pocket?
Pharmacy			
Township health centre			
Village clinics			
Private clinics			

自从上次看病之后，你是否还因为你的病去下列地方寻医问药?	你去了几次?	总共花了多少钱?	你自费付了多少钱?
药店			
乡镇卫生院			
村卫生室			
个体诊所			

Did you seek secondary care for your illness since we talked to you in your consultation?	How many times did you visit?	How much did you pay in total	How much did you pay from your own pocket?
Outpatient			
Inpatient			
Accident and emergency			

自从上次看病之后，你是否因为你的病去医院就诊?	你去了几次?	总共花了多少钱?	你自费付了多少钱?
门诊			
住院			
急诊			

For peer review only - http://bmjopen.bmj.com/site/about/guidelines.xhtml

After the consultation when we recruited you, have you had any treatment for this illness that wasn't prescribed in your consultation?	Prescribed by other doctors	Bought from pharmacy	Obtained from other source (home, family, friends)	How much did you pay in total?	How much did you pay from your own pocket?
**Anti-bacterial/anti-inflammation medicine**					
☐ Amoxicillin	Y/N	Y/N	Y/N	¥	¥
☐ Erythromycin	Y/N	Y/N	Y/N	¥	¥
☐ Cefradine	Y/N	Y/N	Y/N	¥	¥
☐ Levofloxacin	Y/N	Y/N	Y/N	¥	¥
☐ Cefuroxime	Y/N	Y/N	Y/N	¥	¥
☐ Other anti-bacterial/anti-inflammation medicine [please specify]	Y/N	Y/N	Y/N	¥	¥
**Other medicines/treatment**					
☐ Paracetamol	Y/N	Y/N	Y/N	¥	¥
☐ Ganmaoling Granules （e.g. 999 cold remedy）	Y/N	Y/N	Y/N	¥	¥
☐ Qingkailing	Y/N	Y/N	Y/N	¥	¥
☐ Yinhuang Granules	Y/N	Y/N	Y/N	¥	¥
☐ Xiongju Shangqing	Y/N	Y/N	Y/N	¥	¥
☐ Lianhua (lotus) Qingwen capsules	Y/N	Y/N	Y/N	¥	¥
☐ Cough syrup	Y/N	Y/N	Y/N	¥	¥
☐ Ambroxol Hydrochloride oral suspension	Y/N	Y/N	Y/N	¥	¥
☐ Keteling	Y/N	Y/N	Y/N	¥	¥
☐ Compound liquorice tablets	Y/N	Y/N	Y/N	¥	¥
☐ Vitamin C	Y/N	Y/N	Y/N	¥	¥
☐ Crystal sugar snow pear	Y/N	Y/N	Y/N	¥	¥
☐ Ginger	Y/N	Y/N	Y/N	¥	¥
☐ Mugwort	Y/N	Y/N	Y/N	¥	¥
☐ Pipa (loquat)	Y/N	Y/N	Y/N	¥	¥
☐ Orange peel granules	Y/N	Y/N	Y/N	¥	¥
☐ Other [please specify]	Y/N	Y/N	Y/N	¥	¥

在最初看病之后，你是否自己用过了其他不是这次看病是所开具的药物？	其他医生开具	从药店购买	其他方法获得（家中储存，家人，朋友）	这些药总共多少钱？	你自费付了多少钱？
**抗菌药/消炎药**					
☐ 阿莫西林	是/否	是/否	是/否	¥	¥
☐ 红霉素	是/否	是/否	是/否	¥	¥
☐ 头孢拉定	是/否	是/否	是/否	¥	¥

For peer review only - http://bmjopen.bmj.com/site/about/guidelines.xhtml

☐ 左氧氟沙星	是/否	是/否	是/否	¥	¥
☐ 头孢呋辛	是/否	是/否	是/否	¥	¥
☐ 其他抗菌药/消炎药（请注明）	是/否	是/否	是/否	¥	¥
**其他药物**					
☐ 扑热息痛	是/否	是/否	是/否	¥	¥
☐ 感冒灵颗粒（如 999 感冒灵）	是/否	是/否	是/否	¥	¥
☐ 清开灵	是/否	是/否	是/否	¥	¥
☐ 银黄颗粒	是/否	是/否	是/否	¥	¥
☐ 芎菊上清	是/否	是/否	是/否	¥	¥
☐ 莲花清瘟胶囊	是/否	是/否	是/否	¥	¥
☐ 咳嗽糖浆	是/否	是/否	是/否	¥	¥
☐ 盐酸氨溴索口服液	是/否	是/否	是/否	¥	¥
☐ 咳特灵	是/否	是/否	是/否	¥	¥
☐ 复方甘草片	是/否	是/否	是/否	¥	¥
☐ 维生素 C	是/否	是/否	是/否	¥	¥
☐ 冰糖雪梨	是/否	是/否	是/否	¥	¥
☐ 姜	是/否	是/否	是/否	¥	¥
☐ 艾草	是/否	是/否	是/否	¥	¥
☐ 琵琶	是/否	是/否	是/否	¥	¥
☐ 陈皮颗粒	是/否	是/否	是/否	¥	¥
☐ 其他（请注明）	是/否	是/否	是/否	¥	¥

Productivity loss 生产力下降

**If you work, how many days in total have you taken off work due to this illness?**	如果你工作，那么因为这次生病你总共耽误了几天上班？
{number} days	（ ）天
**If you are in full time education, please state how many days in total have you missed your education?**	如果你是学生，那么因为这次生病你总共耽误了几天上学？
{number} days	（ ）天

For peer review only - http://bmjopen.bmj.com/site/about/guidelines.xhtml

EQ-5D-5L

Health Questionnaire

English version for the UK

VERSION FOR INTERVIEWER ADMINISTRATION

Note to interviewer: although allowance should be made for the interviewer's particular style of speaking, the wording of the questionnaire instructions should be followed as closely as possible. In the case of the EQ-5D-5L descriptive system on page 2 of the questionnaire, the precise wording must be followed.

If the respondent has difficulty choosing a response, or asks for clarification, the interviewer should repeat the question word for word and ask the respondent to answer in a way that most closely resembles his or her thoughts about his or her health today.

INTRODUCTION

(Note to interviewer: please read the following to the respondent.)

We are trying to find out what you think about your health. I will explain what to do as I go along, but please interrupt me if you do not understand something or if things are not clear to you. There are no right or wrong answers. We are interested only in your personal view.

First, I am going to read out some questions. Each question has a choice of five answers. Please tell me which answer best describes your health TODAY.

Do not choose more than one answer in each group of questions.

(Note to interviewer: first read all five options for each question. Then ask the respondent to choose which one applies to him/herself. Repeat the question and options if necessary. Mark the appropriate box under each heading. You may need to remind the respondent regularly that the timeframe is TODAY.)

For peer review only - http://bmjopen.bmj.com/site/about/guidelines.xhtml

EQ-5D DESCRIPTIVE SYSTEM

MOBILITY

First, I would like to ask you about mobility. Would you say that:

1. You have no problems in walking about? ❑
2. You have slight problems in walking about? ❑
3. You have moderate problems in walking about? ❑
4. You have severe problems in walking about? ❑
5. You are unable to walk about? ❑

SELF-CARE

Next, I would like to ask you about self-care. Would you say that:

1. You have no problems washing or dressing yourself? ❑
2. You have slight problems washing or dressing yourself? ❑
3. You have moderate problems washing or dressing yourself? ❑
4. You have severe problems washing or dressing yourself? ❑
5. You are unable to wash or dress yourself? ❑

USUAL ACTIVITIES

Next, I would like to ask you about usual activities, for example work, study, housework, family or leisure activities. Would you say that:

1. You have no problems doing your usual activities? ❑
2. You have slight problems doing your usual activities? ❑
3. You have moderate problems doing your usual activities? ❑
4. You have severe problems doing your usual activities? ❑
5. You are unable to do your usual activities? ❑

PAIN / DISCOMFORT

Next, I would like to ask you about pain or discomfort. Would you say that:

1. You have no pain or discomfort? ❑
2. You have slight pain or discomfort? ❑
3. You have moderate pain or discomfort? ❑
4. You have severe pain or discomfort? ❑
5. You have extreme pain or discomfort? ❑

ANXIETY / DEPRESSION

Finally, I would like to ask you about anxiety or depression. Would you say that:

1. You are not anxious or depressed? ❑
2. You are slightly anxious or depressed? ❑
3. You are moderately anxious or depressed? ❑
4. You are severely anxious or depressed? ❑
5. You are extremely anxious or depressed? ❑

For peer review only

For peer review only - http://bmjopen.bmj.com/site/about/guidelines.xhtml

EQ-5D VAS

- **Now, I would like to ask you to say how good or bad your health is TODAY.**
- **I would like you to try to picture in your mind a scale that looks like a thermometer.**
  (Note to interviewer: if interviewing face-to-face, please show the person the VAS scale.)
- **The best health you can imagine is marked 100 (one hundred) at the top of the scale and the worst health you can imagine is marked 0 (zero) at the bottom.**
- **I would now like you to tell me the point on this scale where you would put your health TODAY.**
  (Note to interviewer: mark the scale at the point indicating the respondent's 'health today'. Now, please write the number you marked on the scale in the box below.)

THE RESPONDENT'S HEALTH TODAY = [ ]

Thank you for taking the time to answer these questions.

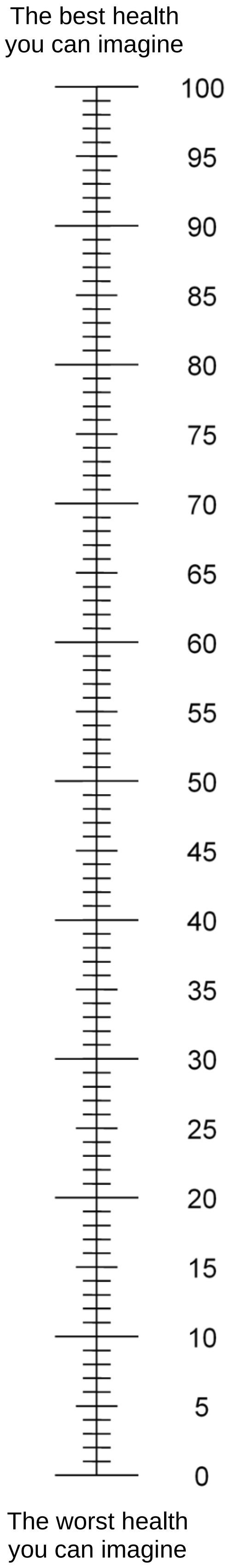

For peer review only

CONSORT 2010 checklist of information to include when reporting a randomised trial*

Section/Topic	Item No	Checklist item	Reported on page No
**Title and abstract**			
	1a	Identification as a randomised trial in the title	Page 1
	1b	Structured summary of trial design, methods, results, and conclusions (for specific guidance see CONSORT for abstracts)	Page 1
**Introduction**			
Background and objectives	2a	Scientific background and explanation of rationale	Page 2
	2b	Specific objectives or hypotheses	Page 3
**Methods**			
Trial design	3a	Description of trial design (such as parallel, factorial) including allocation ratio	Page 3
	3b	Important changes to methods after trial commencement (such as eligibility criteria), with reasons	
Participants	4a	Eligibility criteria for participants	Page 4
	4b	Settings and locations where the data were collected	Page 4
Interventions	5	The interventions for each group with sufficient details to allow replication, including how and when they were actually administered	Page 5
Outcomes	6a	Completely defined pre-specified primary and secondary outcome measures, including how and when they were assessed	Page 5
	6b	Any changes to trial outcomes after the trial commenced, with reasons	
Sample size	7a	How sample size was determined	Page 6
	7b	When applicable, explanation of any interim analyses and stopping guidelines	
Randomisation:			
Sequence generation	8a	Method used to generate the random allocation sequence	
	8b	Type of randomisation; details of any restriction (such as blocking and block size)	Page 5
Allocation concealment mechanism	9	Mechanism used to implement the random allocation sequence (such as sequentially numbered containers), describing any steps taken to conceal the sequence until interventions were assigned	Page 5
Implementation	10	Who generated the random allocation sequence, who enrolled participants, and who assigned participants to interventions	Page 5
Blinding	11a	If done, who was blinded after assignment to interventions (for example, participants, care providers, those	Page 5

For peer review only - http://bmjopen.bmj.com/site/about/guidelines.xhtml

		assessing outcomes) and how	
	11b	If relevant, description of the similarity of interventions	
Statistical methods	12a	Statistical methods used to compare groups for primary and secondary outcomes	Page 6
	12b	Methods for additional analyses, such as subgroup analyses and adjusted analyses	Page 7
**Results**			
Participant flow (a diagram is strongly recommended)	13a	For each group, the numbers of participants who were randomly assigned, received intended treatment, and were analysed for the primary outcome	See figure doc
	13b	For each group, losses and exclusions after randomisation, together with reasons	Page 7
Recruitment	14a	Dates defining the periods of recruitment and follow-up	
	14b	Why the trial ended or was stopped	
Baseline data	15	A table showing baseline demographic and clinical characteristics for each group	See table 1 in page 11
Numbers analysed	16	For each group, number of participants (denominator) included in each analysis and whether the analysis was by original assigned groups	
Outcomes and estimation	17a	For each primary and secondary outcome, results for each group, and the estimated effect size and its precision (such as 95% confidence interval)	See table 1 in page 11
	17b	For binary outcomes, presentation of both absolute and relative effect sizes is recommended	
Ancillary analyses	18	Results of any other analyses performed, including subgroup analyses and adjusted analyses, distinguishing pre-specified from exploratory	
Harms	19	All important harms or unintended effects in each group (for specific guidance see CONSORT for harms)	
**Discussion**			
Limitations	20	Trial limitations, addressing sources of potential bias, imprecision, and, if relevant, multiplicity of analyses	Page 2
Generalisability	21	Generalisability (external validity, applicability) of the trial findings	
Interpretation	22	Interpretation consistent with results, balancing benefits and harms, and considering other relevant evidence	
**Other information**			
Registration	23	Registration number and name of trial registry	Page 1
Protocol	24	Where the full trial protocol can be accessed, if available	NA
Funding	25	Sources of funding and other support (such as supply of drugs), role of funders	Page 8

*We strongly recommend reading this statement in conjunction with the CONSORT 2010 Explanation and Elaboration for important clarifications on all the items. If relevant, we also recommend reading CONSORT extensions for cluster randomised trials, non-inferiority and equivalence trials, non-pharmacological treatments, herbal interventions, and pragmatic trials. Additional extensions are forthcoming: for those and for up to date references relevant to this checklist, see www.consort-statement.org.

For peer review only - http://bmjopen.bmj.com/site/about/guidelines.xhtml
For peer review only

BMJ Open

A cluster randomised controlled trial to assess a tailored intervention to reduce antibiotic prescribing in rural China – Study protocol

Journal:	*BMJ Open*
Manuscript ID	bmjopen-2020-048267.R1
Article Type:	Protocol
Date Submitted by the Author:	22-Jul-2021
Complete List of Authors:	Cong, Wenjuan; University of Bristol, Population Health Sciences<br>Chai, Jing; Anhui Medical University, School of Health Service Management<br>Zhao, Linhai; Anhui Medical University, School of Health Services Management<br>Cabral, Christie; University of Bristol, Population Health Sciences<br>Yardley, Lucy; University of Bristol, School of Psychological Sciences<br>Yao, Guiqing; University of Leicester Department of Health Sciences<br>Zhang, Tingting; University of Bristol, Population Health Sciences<br>Cheng, Jing; Anhui Medical University, School of Health Services Management<br>Shen, XingRong; Anhui Medical University, School of Health Service Management<br>Liu, Rong; Anhui Medical University, School of Health Services Management<br>Little, Paul; University of Southampton, Primary Care and Population Science<br>Stuart, Beth; University of Southampton, Primary Care and Population Science<br>Hu, Xiaowen; Anhui Medical University, School of Health Service Management<br>Sun, Ye-Huan; Anhui Medical University, Department of Epidemiology and Biostatistics<br>Oliver, Isabel; Public Health England South Region, National Infection Service<br>Zheng, Bo; Peking University First Hospital, Institute of Clinical Pharmacology<br>Lambert, Helen; University of Bristol, Population Health Sciences<br>Wang, DeBin; Anhui Medical University, School of Health Services Management
**Primary Subject Heading**:	Public health
Secondary Subject Heading:	General practice / Family practice, Public health
Keywords:	PRIMARY CARE, Respiratory infections < THORACIC MEDICINE, GENERAL MEDICINE (see Internal Medicine)

SCHOLARONE™
Manuscripts

A cluster randomised controlled trial to assess a tailored intervention to reduce antibiotic prescribing in rural China – Study protocol

Wenjuan Cong[1#], Jing Chai[2#], Linhai Zhao[2], Christie Cabral[1*], Lucy Yardley[3], Guiqing Yao[4], Tingting Zhang[1], Jing Cheng[2], XingRong Shen[2], Rong Liu[2], Paul Little[5], Beth Stuart[5], Xiaowen Hu[2], Ye-Huan Sun[6], Isabel Oliver[7], Bo Zheng[8], Helen Lambert[1], Debin Wang[2*]

[1] Population Health Sciences, University of Bristol, Bristol, UK
[2] School of Health Service Management, Anhui Medical University, Hefei, China
[3] School of Psychological Sciences, University of Bristol, Bristol, UK
[4] Department of Health Sciences, University of Leicester, Leicester, UK
[5] Primary Care and Population Science, University of Southampton, Southampton, UK
[6] Department of Epidemiology and Biostatistics, Anhui Medical University, Hefei, China
[7] Public Health England South Region, National Infection Service, Bristol, UK
[8] Institute of Clinical Pharmacology, Peking University First Hospital, Beijing, China

For peer review only

UOB Confidential & Sensitive

Abstract

Introduction: Up to 80% of patients with respiratory-tract infections (RTI) attending healthcare facilities in rural areas of China are prescribed antibiotics, many of which are unnecessary. Since 2009, China has implemented several policies to try to reduce inappropriate antibiotic use; however antibiotic prescribing remains high in rural health facilities.

Methods and analysis: A cluster-randomised controlled trial will be carried out to estimate the effectiveness and cost effectiveness of a complex intervention in reducing antibiotic prescribing at township health centres in Anhui Province, China. 40 Township health centres will be randomised at a 1:1 ratio to the intervention or usual care arms. In the intervention group, practitioners will receive an intervention comprising: i) training to support appropriate antibiotic prescribing for RTI, ii) a computer-based decision support system, iii) virtual peer support, iv) a leaflet for patients and v) a letter of commitment to optimise antibiotic use to display in their clinic.

The primary outcome is the percentage of antibiotics (intravenous and oral) prescribed for RTI patients. Secondary outcomes include patient symptom severity and duration, recovery status, satisfaction, antibiotic consumption. A full economic evaluation will be conducted within the trial period. Costs and savings for both clinics and patients will be considered and quality of life will be measured by EuroQoL (EQ-5D-5L).

A qualitative process evaluation will explore practitioner and patient views and experiences of trial processes, intervention fidelity and acceptability, and barriers and facilitators to implementation.

Ethics and dissemination: Ethical approval was obtained from the Biomedical Research Ethics Committee of Anhui Medical University (Ref: 20180259); the study has undergone due diligence checks and is registered at the University of Bristol (Case No: 2020-3137). Research findings will be disseminated to stakeholders through conferences and peer-reviewed journals in China, the UK and internationally.

Trial Registration: This trial has been registered at ISRCTN (ISRCTN30652037).

[#]WC and JC contributed equally

[*]DW and CC contributed equally

Corresponding Author: Prof Debin Wang; Contact Email: dbwang@vip.sina.com

For peer review only

UOB Confidential & Sensitive

Strengths and limitations of this study

- This is a large cluster-randomised trial to determine whether the intervention is effective in reducing antibiotic prescribing for patients with RTI in township health centres compared with usual care.
- This study will also evaluate the cost-effectiveness of an intervention to reduce antibiotic prescribing in rural areas.
- The quantitative and qualitative research in the trial could also provide recommendations on how to reduce antibiotics prescribing at village clinic level.
- The use of direct observation will ensure accurate assessment of antibiotic prescribing in both baseline and intervention phases.
- The study, though selected to be representative of rural populations in China, includes 10 counties from 4 regions across the Anhui Province, which may have limited generalisability.

Introduction

Antimicrobial resistance (AMR) specifically refers to the evolution of drug-resistant micro-organisms, including bacteria, which is driven by high rates of antibiotic use and is a pressing problem for global health and development.[1-3] If resistance continues to increase, common bacterial infections will eventually become untreatable.[4-5]

Since 2009, China has implemented the national essential medicines scheme and zero-mark-up policies to try to reduce inappropriate antibiotic use. [6-7] In 2012, the Chinese Ministry of Health issued a regulation to limit antibiotic prescribing to 20% of outpatient prescriptions in all patients.[8] However, the antibiotic prescribing rate in healthcare facilities in less developed or rural areas remains as high as 80%.[9-14]

In China patients can choose to go to the outpatient department of any health facility without a referral letter,[15] however, rural residents mainly seek health care from township health centres or village clinics, the two lowest-level health facilities in China's rural health care system. If patients develop severe symptoms, they are more likely to go directly to the outpatient clinic in the county hospital.[15-16] Many patients attend village clinics and township health centres with respiratory tract infections (RTIs) such as common cold, sore throat, and acute bronchitis and are frequently prescribed antibiotics even though most cases are likely to be viral and antibiotics are therefore unnecessary [17-20] Our previous study found that the antibiotic prescribing rate for RTI patients was up to 85% in rural Anhui Province.[21] In addition to the implications for AMR, there is a cost to patients [22-23] for extra health resource utilization (consultation fees, medication costs, loss of productivity costs), most of which would not be reimbursed through national medical insurance schemes. Therefore, reducing antibiotic prescribing in village clinics and township health centres is likely to be both safe and economically beneficial to patients. Since the start of the COVID-19 pandemic, village clinics have periodically been prohibited from providing treatment to RTI patients; therefore this trial focuses on provision of care in township health centres.

Township health centres in China are staffed with both qualified doctors and some health practitioners who are usually referred to as "village practitioners" but would not have a degree in medicine. Village practitioners are required to undergo basic training in health care provided by the local health care authority issued with a medical practice licence. Antibiotic prescribing in township health centres is influenced by uncertainty about which RTI need antibiotics and by widespread beliefs that common

For peer review only - http://bmjopen.bmj.com/site/about/guidelines.xhtml

UOB Confidential & Sensitive

infections should be treated with antibiotics.[15] Practitioners are also concerned about losing patients and believe that prescribing antibiotics helps maintain good relationships with their patients. Public campaigns on antimicrobial stewardship (AMS), [23-25] are very limited in rural areas and there is little technical support for village and township practitioners from senior clinical consultants and healthcare authorities, which makes it difficult for them to change norms of high antibiotic use.

This study will aim to determine whether the intervention safely reduces antibiotic prescribing for patients with RTI in township health centres. The study will provide evidence of effectiveness, value for money and barriers and facilitators to implementation that can inform policy and clinical practice. If pandemic conditions permit, the study will also pilot implementation of the same intervention in a limited number of village clinics to demonstrate proof of concept in lower level health facilities.

Methods and Analysis

Aims and objectives

This study aims to evaluate the effectiveness of a complex intervention aimed at healthcare practitioners and patients at township health centres, to reduce antibiotic prescribing for respiratory-tract infections (RTIs) compared with usual care in township health centres.

The primary outcome will be the percentage of antibiotics (intravenous and oral) prescribed for RTI patients.

Secondary objectives are to:

- estimate the cost-effectiveness of the intervention and the cost implications for both township health centres and patients.
- evaluate the impact of reduced antibiotic use on patient satisfaction with care.
- evaluate the impact of reduced antibiotic use on patient symptom severity, duration and recovery rates.
- investigate patient antibiotic consumption rates (prescribed or obtained from another source).
- understand practitioner and patient views of the intervention and barriers and facilitators to implementation.

Trial design

This is a pragmatic, two-armed (intervention *vs*. usual care) trial with an embedded cost-effectiveness and qualitative evaluations, with randomisation at township health centre level, using observed antibiotic prescribing to assess effectiveness.

Participants and recruitment procedures

Selection of township health centres

We originally started the trial recruitment at village clinic level. However, in May 2021 there was a small local COVID outbreak in Anhui Province, since when village clinics have not been allowed to treat RTI patients in Anhui and our trial recruitment had to be postponed. Currently patients with suspected Covid-19 symptoms (specifically patients with a temperature of more than 37.3 degree) must be referred to specialist COVID clinics or specialist COVID hospitals for treatment. We did some preliminary fieldwork in June 2021 and found it is still very difficult to recruit RTI patients at village clinic level and the research team assessed that this situation may not change within the next 12 months. However, we found that township health centres are still able to treat RTI patients regularly.

For peer review only

UOB Confidential & Sensitive

In order to complete recruitment and maintain trial feasibility, we therefore decide to change the recruitment sites from village clinics to township health centres.

We will recruit 40 township health centres from 10 counties (4 township health centres per county), those counties will be randomly selected from 4 regions geographically dispersed across Anhui Province. We will randomly recruit 6 counties from 2 regions in densely populated North Anhui and 4 counties from another 2 regions from sparsely populated South Anhui. We will seek support from local county health authorities during the recruitment of township health centres. They will provide a list of townships in their area and the research team will randomly select 4 township health centres. Local county health authorities will screen the selected townships and may exclude selected townships, for which a reason will be recorded.

Recruitment of practitioners

The recruitment of health practitioners will be fair. Once a township health centre is recruited and has given permission for its staff to participate, all the practitioners working in the recruited township health centres will be invited to participate in the study. Once practitioners are identified and recruited, informed consent (Appendix 1, Supplementary material) will be obtained immediately.

Exclusion criteria
1, Practitioners who do not have basic computer skills
2, Practitioners who are not involved in clinical diagnosis and medicine prescribing

The background information of participating township health centres and practitioners will be collected. A senior practitioner in the recruited township health centre will complete a clinic background information questionnaire (Appendix 2, Supplementary material); and all practitioners working in the clinic will give their demographic data in a practitioner background information questionnaire (Appendix 3, Supplementary material).

A baseline audit of 400 RTI patients across the 40 township health centres will be conducted (10 RTI patients for each township health centre). This baseline audit will only collect primary outcome data, including the antibiotic prescribing rates at each clinic. Then all the township health centres will be randomised at a 1:1 ratio to usual care group vs intervention group, using the baseline antibiotic prescribing rates to balance high and low prescribing clinics between the arms. Each group will have 20 township health centres. (details see Fig. 1)

Recruitment of patients

All patients with RTIs who seek health care from the recruited health centres will be screened for eligibility and invited to participate in this study. Researchers from Anhui Medical University will undertake patient recruitment on-site until 30 participants have been recruited at each township health centre. Informed consent (Appendix 4, Supplementary material) will be obtained from all the patients who agree to participate in this study.

Currently, patients with suspected Covid-19 symptoms must be referred to specialist hospitals and township health centres are not expected to see suspected Covid-19 patients who present for treatment. However, some of these patients test negative for Covid-19 and are then referred back to their local clinic for treatment for their non-Covid RTI. These patients will be eligible for inclusion in our study.

Eligibility Criteria

For peer review only - http://bmjopen.bmj.com/site/about/guidelines.xhtml

UOB Confidential & Sensitive

Inclusion criteria

1. Adult (aged ≥18 years old)
2. Patient who is diagnosed with a RTIs in the recruitment consultation: blocked/runny nose, coughing with or without sputum, dry/sore throat, breathing problems, fever, ear inflammation (blocked ear, tinnitus, ear discharges, earache).

Exclusion criteria

1. Patients who have previously sought treatment for this illness from a normal clinic (not a specialist Covid clinic).
2. Patients who are unable to provide fully informed consent (eg dementia, psychosis, or severe depression)
3. Patients who are pregnant

Patients without bacterial infections, immunocompromised patients and patients with non-infective conditions are not excluded from this study if they present with any of the symptoms listed in the inclusion criteria. Patients who previously sought treatment for the same episode of illness in other health services (outpatient clinic in the county hospitals etc) will be excluded, simply because the treatment sought elsewhere may bring interference or contamination to our study findings by having previously obtained antibiotics elsewhere.

Randomisation, allocation & blinding

All the recruited township health centres will be stratified by baseline antibiotic prescribing rate quartile and geographical location (north, central, south) and then randomly allocated at a 1:1 ratio to the intervention or usual care group. Antibiotic prescribing rate (%) of each township health centre will be measured by dividing the total number of RTI patients who are prescribed antibiotics (IV and oral) against the total number of RTI patients who are present to each township health centre.

All participating health centres will be allocated to a trial arm at the same time, after completion of baseline data collection. Due to the nature of the intervention, it is not possible to blind practitioners to their allocation to either intervention or usual care group. However, the statisticians conducting the analysis will remain blind to allocation until quantitative analysis is complete. The trial randomisation and blinding will be conducted by an independent statistician.

Intervention

The complex intervention comprises training, peer support, a computerised decision aid, a public commitment letter and a patient leaflet. These were co-designed with practitioners and patients using the person-based approach method for intervention development. [26]

We optimised the intervention using qualitative research to identify barriers to acceptability, feasibility and engagement that need to be addressed. We conducted four rounds of 'think-aloud' interviews with 37 village practitioners and 17 Patients in rural Anhui. Think-aloud interviews obtain detailed user views by asking practitioners and patients to view contents of our intervention tools (training materials for village practitioners, peer support groups, computer-based decision support system, patient leaflet). Findings were immediately tabulated and analysed to identify changes required, modified materials used for the next round of think-aloud interviews and the process repeated until participant feedback indicated no further important, feasible and appropriate changes were needed. Through this process, we created a feasible and generalisable intervention to reduce antibiotic prescribing tailored for the rural Chinese context.

For peer review only - http://bmjopen.bmj.com/site/about/guidelines.xhtml

UOB Confidential & Sensitive

For peer review only

Intervention practitioners will receive training delivered face to face in one session by a senior clinician from a tertiary hospital. The training covers: i) evidence about AMR in China and the link to their own practice; ii) addresses key concerns and misapprehensions; iii) details the current Chinese clinical guidelines on treatment of RTIs; iv) explains how to use the computer-based decision support aid; and v) outlines communication strategies to improve patient acceptance of a non-antibiotic treatment decision.

Peer support groups will be established to connect the village practitioners to others in the intervention group in order to provide peer support to each other during implementation. Peer support groups will meet via a widely used social media app in China (WeChat). At the end of the training session intervention practitioners will be asked to organise themselves into 3-4 sub-groups and identify a lead for each group. The group leader will be provided with a meeting topic guide and be responsible for setting up regular virtual meetings of the peer support group, during which each member of the group will share their experience of applying the intervention, discuss any problems and support each other to find solutions. Practitioners will be encouraged to contact each other outside of the scheduled meetings to ask for and provide support to each other. To ensure their group discussions follow the requirements of the intervention, a senior clinician will be the senior clinical champion for the intervention and a member of all peer support groups and the technical support group. He will oversee the group discussion and make sure it complies with the intervention format for audit and feedback. Additionally, field researchers will also join the Wechat subgroups as observers. They will record the content of all group discussions and anonymize all transcripts for qualitative analysis.

A computer-based decision support system will be installed on computers in consulting rooms in the intervention clinics. This will allow village practitioners to select the patient's symptoms and diagnosis and will then provide the appropriate treatment guidance from the Chinese national guidelines. The decision support system has been adapted from our previous project.[27] It contains the following sections: 1) patient eligibility criteria; 2) patient symptoms: help practitioners identify what symptoms patients are suffering from; 3) diagnosis, patient reassurance & prognosis: assist practitioners to make a diagnosis, and provide appropriate patient reassurance and prognosis for each type of RTI patient; 4) treatment recommendations for each type of RTI patient; 5) Table of symptomatic treatment options for RTI patients (from Chinese National Guidelines); 6) Safety netting advice (when to come back to see the doctor) (Appendix 5, supplementary material)

Village practitioners will be required to sign a public declaration letter stating that they are committed to reducing unnecessary antibiotic prescribing and display this in a public area of their clinic.

Township health centres will be provided with a leaflet that can be given to RTI patients. This leaflet will address common patient beliefs and concerns about RTI, address common misapprehensions that can lead to an expectation of antibiotic treatment, provides advice on non-antibiotic symptomatic treatment options and safety-netting.

Data collection methods

Baseline data will be collected for 400 patients with RTI at the participating township health centres. Follow-up data collection will be collected for 1200 patients at the participating township health centres after implementation of the intervention.

In all recruited township health centre, direct observations will be carried out by a researcher from Anhui Medical University following the doctor questionnaire (Appendix 6, supplementary material) adapted from our previous study.[16] Observation will focus on the adult RTI clinical consultations and

For peer review only - http://bmjopen.bmj.com/site/about/guidelines.xhtml

UOB Confidential & Sensitive

record patient symptoms, practitioner's diagnosis & treatment, sickness severity, any antibiotics or other medicines prescribed and mode of administration (IV or oral), name, dosage and quantity. Immediately after the consultation, the researcher will fill in a patient questionnaire (Appendix 7, supplementary material) by asking questions of patients directly. This questionnaire is also adapted from the previous study [16] and includes questions about patient demographic characteristics (gender, age, education level, residential area and medical insurance records), illness duration, symptom severity and patient satisfaction with the consultation; the latter two questions will be scored using a 10-point scale.

The researcher will conduct telephone interviews with recruited RTI patients at 7, 14, 21 days after their consultation. Structured questionnaires will be completed for each patient by the researcher during the telephone interview, with different secondary outcome data collected at different time points (see Table 1). Illness severity (scored on a 10-point scale) and symptom duration will be recorded at each time point. Patient attitude, understanding of the patient leaflet and beliefs will be recorded once at 7 days after consultation (Appendix 8, supplementary material). Patient continuing illness severity and recovery situation (when they feel better & when they feel they could get back to normal) will be checked at 7 days (Appendix 8, and 14 days after consultation (Appendix 9, Supplementary material). Patient consumption of antibiotics (including name, days, dosage and quantity) will be recorded at 7, 14 and 21 days after consultation (Appendix 10, Supplementary material). Through checking with patient's actual consumption of antibiotics (how many days they have taken the antibiotics, dosage etc) at different periods against the prescribed quantity of antibiotics (if applicable), patient adherence to follow practitioner's advice (%) could be estimated. Other healthcare use (clinic visits or consumption of other treatment) will be recorded at 21 days after consultation. Patient out-of-pocket spending due to this illness, including purchase of medication, fees for clinic attendance, days off-work , will be recorded at 21 days after consultation. Patient quality of life will be measured using the EQ5D-5L during the initial consultation, at 7, 14 & 21 days after consultation. (Use of paper interview administration mode of EQ5D-5L in Chinese version has been authorized by EuroQol Group (registration ID: 32880).

Data analysis

The primary analysis will use a generalised linear mixed model with a logit link and a random effect for clinic, allowing for the clustering of participants within clinics. The model will control for the clinic-level baseline antibiotic prescribing rate as well as potential confounders, and the full list of model covariates will be prespecified and set out in the statistical analysis plan. Results will be reported as odds ratios with 95% confidence intervals.

Missing data will be treated as missing at random and imputed using a chained-equations multiple imputation model. A complete cases analysis will be presented as a secondary analysis.

The analysis of secondary outcomes will use a similar modelling framework with distributions appropriate to the outcome, e.g. linear modelling for continuous outcomes and Poisson/negative binomial for count outcomes. These models will control for clustering and the factors listed above.

All results will be reported in line with the Cluster Randomised Trials extension to the CONSORT guidelines. No interim analyses are planned. Full details of the analyses to be undertaken will be set out in the Statistical Analysis Plan and approved by the Trial Steering Committee (TSC)

Sample size calculation

For peer review only

UOB Confidential & Sensitive

Our previous study recorded an antibiotic prescribing rate of 75% for rural township health centres in Anhui province [21, 28] and a previous pilot intervention showed a reduction in antibiotic prescribing of more than 20%. [27] There is no internationally agreed minimally important difference in antibiotic prescribing, however we anticipate that a reduction of at least 15% in antibiotic prescribing would represent a meaningful change at the population level given the very high rates of antibiotic prescribing.

To detect a possible absolute difference of 15% in prescribing rates between intervention (60%) and control practices (75%) with 90% power and alpha 0.05, we will need 203 patient consultations for infection RTI in each arm. Based on our previous study results the estimated ICC value was 0.05 and a design effect value would be 2.45. We would therefore aim to recruit at least 2.45*203*2=995 patients into the study. Allowing for a loss to follow-up rate of 10% and a cluster size of 30 patients per township health centre, we will recruit 40 township health centres into the study and each arm will have 20 township health centres.

Economic evaluation

The economic evaluation will be taken from a societal perspective and consider cost implications for patients as well as health services. Resource usage information will be costed using corresponding Chinese tariff.

All itemised resource usage data will be costed using appropriate data, with unit costs of visiting health care facilities based on medical service price items. Medication based on clinical consultation patient questionnaire and phone interviews with the patients at 7, 14 and 21 days; and the time spent costed at average wage in Anhui Province. Insurance coverage will be weighted to calculate private spending.

Resource use will be weighted by its unit cost over the study period. Accumulated costs per patient will be calculated for the study period. Quality of life data will be translated into a utility score using a Chinese tariff. Quality adjusted life years will be estimated using the area under the curve approach.

Missing data will be handled by multiple imputation methods. Difference in costs and QALYs will be compared using a generalised linear mixed model taking clinic as cluster level and by adjusting baseline clinical and quality of life variables. Bootstrapping methods will be used to estimate the incremental cost per QALYs gained and per percentage reduction of antibiotic prescribing. Major assumptions will be explored through sensitivity analyses. Completed cases analyses will be presented as one of such scenario analyses.

Qualitative Process Evaluation

A Mandarin-speaking qualitative researcher will conduct observations of the training process, peer support discussions and intervention implementation. The key content related to participants during the training process, including any additions by the trainer, questions and answers, comments or discussion among participants and non-verbal reactions displayed by participants will be recorded in field notes. The researcher will be added as an observer into the WeChat groups to record the content of peer support discussions including participants' queries, examples of challenges and solutions and any other discussion. The researcher will visit intervention clinics after follow-up data collection is completed and observe how the intervention is being implemented.

A Mandarin-speaking qualitative researcher will conduct semi-structured interviews with health professionals. Short interviews will be conducted at the end of the training, to elicit intervention practitioners' views about the training. Following completion of follow up date collection, intervention practitioners will be invited to participate in a semi-structured interview to understand their

For peer review only

UOB Confidential & Sensitive

experiences of the intervention. They will be asked to describe how they used the intervention in their practice, any barriers and facilitators encountered in following the intervention and whether it influenced their views or practice.

A purposive sample of up to 40 patients who are recruited into the study will be interviewed. Patient will be selected to capture a range of socio-demographic characteristics and illness presentations. Some will be interviewed immediately after completing their consultation with health practitioners and some after they have completed the telephone questionnaires. Interviews will be semi-structured and guided by a topic guide that will cover experience of the consultation and understandings of the patient leaflet they received.

All semi-structured interviews will be transcribed. The qualitative data will be analysed thematically.

Data handling and repository

This trial will be monitored by a Trial Steering Committee and collected data will be monitored by data monitoring committee. There are no anticipated risks to patients from taking part in the study and participants have the right to withdraw from the study at any time without giving a reason.

Hard copies of documents with identifiable details of patient, practitioner and clinic will be stored in lockable cabinets on premises approved by the PI or relevant Co-Is at Anhui Medical University and any data created will be encrypted and stored on University secure servers until they can be uploaded or scanned and stored in the University of Bristol Research Data Storage Facility (RDSF) at the first available opportunity. Only authorised users can access data stored within the RDSF and procedures will be put in place to allow authenticated, external collaborators to view, add and/or edit data.

When possible, data will be exported directly to databases and consistency maintained via peer review and cross-checks with previous measurements. Manually inputted data will be double entered. Quality of questionnaire and interview data will be assured by training, provision of an interview manual and re-survey of 5% of subjects.

Patients and public involvement

Patients (and practitioners) contributed to the co-design of the intervention. They also provided feedback on piloted data collection forms, which led to modifications. The results will be disseminated to study participants and other stakeholders. At the end of the research project we will hold a high-level meeting to disseminate our findings to key stakeholders and develop a strategy for impact with relevant stakeholders.

ETHICS and dissemination

Ethical approval was obtained from the Biomedical Research Ethics Committee of Anhui Medical University (Ref: 20180259); the study has undergone due diligence checks and is registered at the University of Bristol (Case No: 2020-3137). This project is one of the three work packages in the UK-China AMR Centre Partnerships 2018 (Strategies to reduce the burden of antibiotic resistance in China) supported by UKRI Medical Research Council (Grant Ref: MR/S013717/1)) and National Natural Science Foundation of China (Grant Ref: 81661138001). The protocol is rooted in a multidisciplinary collaboration between institutions in the UK and China.

Throughout the research project we will involve key stakeholders with roles in managing antibiotic use and antimicrobial stewardship. Research findings will be translated into short policy briefings with recommendations informed by existing policies in China and to influence current practice in health

For peer review only

UOB Confidential & Sensitive

care settings. The credibility of research findings will be indicated to stakeholders through peer-reviewed publications in internationally recognised journals and presentations at academic conferences in China, the UK and internationally.

Author Contributions

HL, DW, CC, LY were responsible for developing the research questions and HL, DW, CC, LY, PL, BS, GY, IO, BZ were responsible for the study design. TZ, JC, JC, XS, RL, XH, YS are responsible for data collection and WC, JC & LZ are responsible for study management and coordination. WC, JC, BS, TZ, GY and CC drafted the paper. All authors read, commented on and approved the final manuscript.

Competing interests statement

None declared

Funding

The authors acknowledge MRC & Newton Fund through a UK-China AMR Partnership Hub award (MR/S013717/1), and National Natural Science Foundation of China award (81861138049).

References

1. Tenover FC. Mechanisms of Antimicrobial Resistance in Bacteria. *Am J Med*. 2006;119(6, Supp1):S3-S10.
2. Antimicrobial resistance: 2014 global report on surveillance. Geneva: World Health Organization; 2014.
3. Holmes AH, Moore LSP, Sundsfjord A, et al. Understanding the mechanisms and drivers of antimicrobial resistance. *Lancet*. 2016;387(10014):176-187.
4. Levy SB, Marshall B. Antibacterial resistance worldwide: causes, challenges, and responses. *Nat Med*. 2004;10(12):S122-S129.
5. Laxminarayan R, Matsoso P, Pant S, et al. Access to effective antimicrobials: a worldwide challenge. *Lancet*. 2016;387(10014):168-175.
6. Mao W, Chen W. CHINA: The Zero Mark-up Policy for essential medicines at primary level facilities. Health Systems, Governance & Financing, World Health Organization; 2015.
7. Xiao Y, Li L. China's national plan to combat antimicrobial resistance. *Lancet Infect Dis*. 2016;16(11):1216-1218.
8. Xiao Y, Li L. Legislation of clinical antibiotic use in China. *Lancet Infect Dis*. 2013;13(3):189-191.
9. Yang L, Liu C, Ferrier JA, et al. The impact of the National Essential Medicines Policy on prescribing behaviours in primary care facilities in Hubei province of China. *Health Policy Plan*. 2013;28(7):750-760.
10. Wang J, Wang P, Wang X, et al. Use and Prescription of Antibiotics in Primary Health Care Settings in China. *JAMA Intern Med*. 2014;174(12):1914-1920.
11. Wei X, Zhang Z, Walley JD, et al. Effect of a training and educational intervention for physicians and caregivers on antibiotic prescribing for upper respiratory tract infections in children at primary care facilities in rural China: a cluster-randomised controlled trial. *Lancet Glob Health*. 2017;5(12):e1258-e1267.
12. Guan X, Tian Y, Song J, et al. Effect of physicians' knowledge on antibiotics rational use in China's county hospitals. *Soc Sci Med*. 2019;224:149-155.
13. Zhan Q, Wang YL, Chen X. Evaluation of antibacterial use in outpatients of township and community primary medical institutions in a district of Sichuan Province, China. *J Glob Antimicrob Resist*. 2019;19:201-206.
14. Wang CN, Huttner BD, Magrini Net al. Pediatric Antibiotic Prescribing in China According to the 2019 World Health Organization Access, Watch, and Reserve (AWaRe) Antibiotic Categories. *J Pediatr*. 2020;220:125-131.e5.
15. Lambert H, Chen M, Cabral C. Antimicrobial resistance, inflammatory responses: a comparative analysis of pathogenicities, knowledge hybrids and the semantics of antibiotic use. *Palgrave Commun*. 2019;5(1):85.

For peer review only

UOB Confidential & Sensitive

16. Zhao L, Kwiatkowska RM, Chai J, et al. Pathways to optimising antibiotic use in rural China: identifying key determinants in community and clinical settings, a mixed methods study protocol. *BMJ Open*. 2019;9(8):e027819.
17. Gulliford MC, Dregan A, Moore MV, et al. Continued high rates of antibiotic prescribing to adults with respiratory tract infection: survey of 568 UK general practices. *BMJ Open*. 2014;4(10):e006245.
18. Zumla A, Al-Tawfiq JA, Enne VI, et al. Rapid point of care diagnostic tests for viral and bacterial respiratory tract infections—needs, advances, and future prospects. *Lancet Infect Dis*. 2014;14(11):1123-1135.
19. Godman B, Haque M, McKimm J, et al. Ongoing strategies to improve the management of upper respiratory tract infections and reduce inappropriate antibiotic use particularly among lower and middle-income countries: findings and implications for the future. *Curr Med Res Opin*. 2020;36(2):301-327.
20. Wang CN, Huttner BD, Magrini N, et al. Pediatric Antibiotic Prescribing in China According to the 2019 World Health Organization Access, Watch, and Reserve (AWaRe) Antibiotic Categories. *J Pediatr*. 2020;220:125-31.e5.
21. Chai J, Coope C, Cheng J, et al. Cross-sectional study of the use of antimicrobials following common infections by rural residents in Anhui, China. *BMJ Open* 2019; 9:e024856.
22. Song J-H, Thamlikitkul V, Hsueh P-R. Clinical and economic burden of community-acquired pneumonia amongst adults in the Asia-Pacific region. *Int J Antimicrob Agents*. 2011;38(2):108-117.
23. Wang X, Xuan Z, Storella TH, et al. Determinants of non-prescription antibiotic dispensing in Chinese community pharmacies from socio-ecological and health system perspectives. *Soc Sci Med*. 2020:113035.
24. Xiao Y, Shen P, Zheng B, et al. Change in Antibiotic Use in Secondary and Tertiary Hospitals Nationwide After a National Antimicrobial Stewardship Campaign Was Launched in China, 2011-2016: An Observational Study. *J Infect Dis*. 2020;221(2):S148-S155.
25. Xiao Y. Antimicrobial Stewardship in China: Systems, Actions and Future Strategies. *Clin Infect Dis*. 2018;67:S135-S141.
26. Yardley L, Morrison L, Bradbury K, et al. The person-based approach to intervention development: application to digital health-related behavior change interventions. *J Med Internet Res.* 2015;17(1):e30.
27. Shen X, Lu M, Feng R, et al. Web-Based Just-in-Time Information and Feedback on Antibiotic Use for Village Doctors in Rural Anhui, China: Randomized Controlled Trial. *J Med Internet Res*. 2018;20(2):e53.
28. Shen X, Shen J, Pan Y, et al. Clinical diagnosis and treatment of common respiratory tract infections in relation to microbiological profiles in rural health facilities in China: implications for antibiotic stewardship. *BMC Fam Pract* 2021; 22:87-95.

For peer review only

UOB Confidential & Sensitive

Table 1 List of the primary and secondary outcome measures and when they will be collected at follow-up audit.

Item	Outcome measure	Initial consultation	Patient Telephone interview after consultation		
			7 days	14 days	21 days
Primary outcome	Prescribing rate of antibiotics (%)	√			
Secondary outcomes	Patient satisfaction	√			
	Patient symptom severity and duration		√	√	√
	Patient recovery		√	√	
	Patient consumption of antibiotics		√	√	√
	Patient attitude, beliefs and understanding of the patient leaflet		√		
	Patient costs				√
	Patient quality of life (EQ5D-5L)	√	√	√	√

Figure legend

Fig 1 Trial flow chart

Supplementary material

Appendix 1 Health practitioner consent form
Appendix 2 Patient consent form
Appendix 3 Health practitioner consent form
Appendix 4 Practitioner background information questionnaire
Appendix 5 Computer-based decision support system content
Appendix 6 Clinical consultation doctor questionnaire
Appendix 7 Clinical consultation patient questionnaire
Appendix 8 Telephone questionnaire – Day 7
Appendix 9 Telephone questionnaire – Day 14
Appendix 10 Telephone questionnaire – Day 21

For peer review only

UOB Confidential & Sensitive

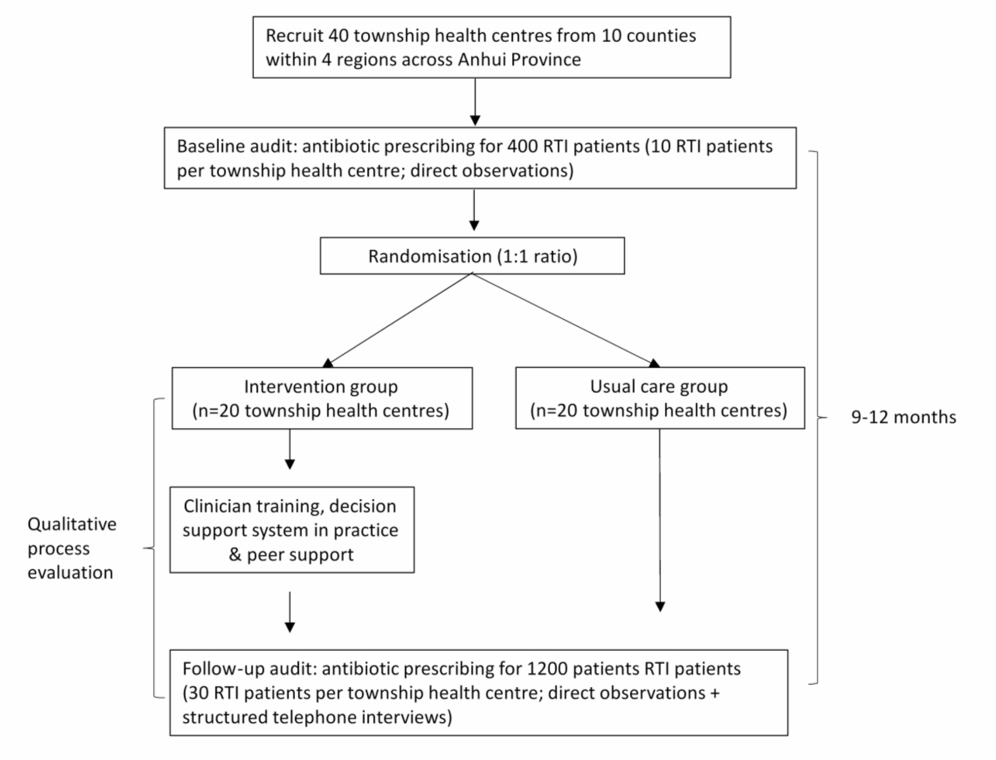

Trial flow chart

196x149mm (300 x 300 DPI)

Study Number:

Participated Clinic Identification Number for this trial:

CONSENT FORM

Title of Project: Design and evaluate a tailored intervention to modify antibiotic prescribing behaviour among health professionals and reduce antibiotic consumption: a cluster randomised controlled trial

Name of Researcher:

Please initial all boxes

1. I confirm that I have read and understand the information sheet dated [**DATE**] (version 1) for the above study. I have had the opportunity to consider the information, ask questions and have had these answered satisfactorily.

2. I understand that my participation is voluntary and that I am free to withdraw at any time without giving any reason, without my medical care or legal rights being affected.

3. I understand that any information I provide will be anonymised by removing all identifying details and will be kept strictly confidential and used only for research purposes. After the study, the anonymised information will be made publicly available for potential further research, but it will not be possible to identify me from the data.

4. I understand that with my agreement, the interview may be audio-recorded but no one except the research team will hear this recording. I am aware that anonymised quotes from the interview may be included in reports of the study findings.

5. I agree to take part in the above study.

**Name of Participant**	**Date**	**Signature**

**Name of Person taking consent (if applicable)**	**Date**	**Signature**

**Name of Researcher**	**Date**	**Signature**

Consent form date of issue: **[DATE]**
Consent form version number: **[VERSION]**

For peer review only - http://bmjopen.bmj.com/site/about/guidelines.xhtml

Township health centre (THC) background information questionnaire

乡镇卫生院基本情况调查表

This questionnaire should be filled by the head of the THC
这张问卷请由此乡镇卫生院的负责医生填写。

名称：________（市）________县（区）_________乡（镇）___________村
_______township (town),_______county, _______city

一、乡镇卫生院的基本情况

Basic information of THC

1、按照设置规划，本乡镇卫生院服务<br>1．1 覆盖地域面积为_______平方公里；<br>1．2 服务覆盖总人口__________人；<br>1．3 其中，常住人口________人；	1.The information about services of the township health centre<br>1.1 What is the service coverage area? _________square kilometers;<br>1.2 What is the total population covered by the service? ______;<br>1.3 What is the number of permanent residents covered by the service? _______;
2、目前，本乡镇卫生院所服务的行政村，实际共有其他村级医疗机构______个，并请说明医疗机构名称_______	2. At present, the administrative townships served by the THC actually have ______other township level medical institutions, *[please specify their names]* ___________
3、是否提供中医服务？（1）是 （2）否<br>3.1 包括哪些中医服务？<br>□草药；□针灸；□推拿；□其他______	3. Is TCM service provided here?<br>(1) Yes; (2) No<br>3.1 What TCM services are included?<br>□ herbal; □acupuncture; □ massage; □ other *[please specify]* _________
4、本乡镇卫生院能提供的医学检查有哪些？<br>□ 血液检查<br>□ X 射线<br>□ CT<br>□ B 超<br>□ 心电图检查<br>□ 没有<br>□ 其他检查手段，请描述______	4. What kinds of RTI related tests can this THC provide?<br>□Blood test<br>□X-Ray<br>□CT<br>□B Ultrasound<br>□ECG<br>□None<br>Other (Please Specify) _______
5、本乡镇卫生院有医生数量_________个.	5. The THC has_____ outpatient clinics; these clinics have _____doctors in total.

For peer review only

二、乡镇卫生院的医疗服务情况

Medical services in village clinics

2.过去一年接诊多少门诊病人？__________<br>2．1 其中，呼吸道感染的病人多少人？___	2. How many outpatients are there in the past year?<br>2.1 How many patients with respiratory infection?

For peer review only

Health practitioner background information questionnaire

乡镇卫生院医生基本情况调查表

This questionnaire should be filled by all clinicians working in this township health centre.

全部在此乡镇卫生院工作的医生都需要填写此表。

Gender?<br>□Male<br>□Female	性别?<br>□男<br>□女
Date of birth? ___________<br>Age: _____years.	出生日期：_____年_____月_____日；<br>或年龄？______岁
Education?<br>□ middle school and below;<br>□ high school /polytechnic school / technical school;<br>□ junior college;<br>□ undergraduate;<br>□ postgraduate and above;<br>□ others, please specify_____	学历？<br>□初中及以下；<br>□高中/中专/技校；<br>□专科；<br>□大学本科；<br>□研究生及以上；<br>□其他，请说明_____
How many years of medical training have you received? _______years;	你受到多少年的医学专业训练？<br>_______年；
What kind of medical training/learning do you have? What is your specialty?<br>□ Clinical medicine;<br>□ TCM;<br>□ Nursing;<br>□ Others, please specify_______	所学专业？<br>□临床医学；<br>□中医学；<br>□护理学；<br>□其他，请说明_______
How long have you worked as a physician? _______years;	您从事医生的工作年限？ _______年；
How long have you worked here? _______years;	你在这里工作多久了？ _______年；
Type of certificate you obtained:<br>□ licensed physician;<br>□ Certified assistant physician;<br>□ Registered nurse;<br>□ Village doctor;<br>□ No certificate	你获得证书类别：<br>□执业医师；<br>□执业助理医师；<br>□注册护士；<br>□乡村医生；<br>□无证书
Category of doctor practice:<br>□ Clinical;<br>□ Chinese medicine;<br>□ Public health<br>□ Others, please specify_______	医师执业类别：<br>□临床；<br>□中医；<br>□公共卫生<br>□其他，请说明_______
Do you have any further study experience? How many months in total?;<br>□ Yes (if yes, total months? _______months) ;<br>□ No;	你有没有进修的经历？<br>□有（如果有，共_____个月？）；<br>□无；
Have you ever participated in the training on the use of antibiotics? □Yes;□No;<br>If yes, how many times? _______times;<br>How many months in total? _______months.	有没有参加过关于抗菌药物使用的培训？<br>如果有，几次？<br>累计多少个月？

For peer review only

Study Number:

Patient Identification Number for this trial:

CONSENT FORM

Title of Study: Appropriate use of antibiotics for RTI in primary care: a cluster randomised controlled trial

Name of Researcher: XXX (contact details)

	**Please initial all boxes**
I confirm that I have read information sheet /went through information sheet with the researcher. I understand it and has had the opportunity to ask questions.	
I understand that my participation is voluntary and that I am free to withdraw at any time without giving any reason, without my medical care being affected.	
I understand that all the information is confidential. No name or personal information will be used when the results of this study is published.	
I agree to take part in the above study.	

Name of Participant **Date** **Signature**

Name of Person taking consent

(if applicable) **Date** **Signature**

Name of Researcher **Date** **Signature**

**Participant contact details (used for telephone questionnaire)**	
Phone number	
WeChat ID	

Consent form date of issue: **[DATE]**
Consent form version number: **[VERSION]**

For peer review only - http://bmjopen.bmj.com/site/about/guidelines.xhtml

Decision Support System & in clinic data collection items – for HP to use within consultation 诊断支持系统

Page 1: Eligibility criteria

第一页：入选条件

Does patient have a suspected RTI?	病人是否疑似呼吸系统疾病?
☐ Yes<br>☐ No – exclude from study	☐ 是<br>☐ 否—从本研究中排除
**Is today the first time the patient has sought treatment for this illness or a repeat visit?**	**今天是病人为此病的首次还是再次就诊?**
☐ First time consultation<br>☐ Repeat consultation – exclude from study	☐ 首次就诊<br>☐ 再次就诊--从本研究中排除
**Does patient need to be transferred due to COVID-19 suspected symptoms?**	**病人是否因疑似新冠需要被转入发热门诊?**
☐ Yes – exclude from study<br>☐ No	☐ 是 – 从本研究中排除<br>☐ 否

Page 2: Patient Symptoms

第二页：病人症状

Consultation – Respiratory illness – Initial consultation – Illness history	
**What symptoms are patient suffering from?** (tick all that apply)	**病人具体都有哪些症状?** (勾选所有符合的症状)
☐ Nasal congestion and running nose<br>☐ Cough<br>☐ Expectoration<br>☐ Sore throat<br>☐ Congestion/swelling of tonsils<br>☐ Fever<br>☐ Headache<br>☐ Earache	☐ 鼻塞，流鼻涕<br>☐ 咳嗽<br>☐ 咳痰<br>☐ 嗓子疼<br>☐ 扁桃体肿大<br>☐ 发烧<br>☐ 头疼<br>☐ 耳朵疼

For peer review only

☐ Muscular stiffness<br>☐ Chest congestion or shortness of breath<br>☐ Others	☐ 肌肉僵硬<br>☐ 胸闷或呼吸急促<br>☐ 其他______________

Page 3 : Diagnosis, prognosis & patient reassurance

第三页：诊断，预后及病人安慰

Tick one	**What is the diagnosis?**	选一项打勾	**诊断是什么?**
	1. Acute upper respiratory tract infection including:<br>• Infectious cold / Acute rhinitis / upper respiratory tract catarrh<br>• Acute inflammation of the throat<br>• Acute viral pharyngitis<br>• Acute viral laryngitis		1.急性上呼吸道感染:<br>• 传染性感冒/急性鼻炎/上呼吸道粘膜炎<br>• 喉咙急性炎症<br>• 急性病毒性咽炎<br>• 急性病毒性喉炎
	2. Acute tonsil inflammation / Tonsillitis / Pharyngitis		2.急性化脓性扁桃体炎/ 扁桃体炎/咽炎
	3. Acute ear inflammation / Otitis Media		3.急性耳朵炎症/中耳炎
	4. Acute sinusitis		4.急性鼻窦炎
	5. Acute tracheal/ bronchial inflammation / Acute Tracheobronchitis / Bronchitis / Lower respiratory tract infection		5.急性气管/支气管炎症/急性气管支气管炎/支气管炎/下呼吸道感染
	6. Pneumonia		6.肺炎
	Other please state [free text box]		其他请说明

**Patient Reassurance**	**病人安慰**
• If you have diagnosed condition 1-5 reassure the patient that there is no sign of serious infection. Here are some things you can say to reassure patients:<br>○ There is no evidence of a serious infection<br>○ In your case the body can fight infections well on its own<br>○ You can take remedies to ease the symptoms<br>• If you have diagnosed Pneumonia, move straight on to Prognosis (next step) and Treatment (page 4).	• 如果您确诊病人所患疾病为 1-5 中之一，请确保该病人没有严重感染的迹象。以下是一些可以让患者放心的语句:<br>○ 没有严重感染的证据<br>○ 就你的情况而言，你的身体可以自己抵抗感染<br>○ 您可以使用一些疗法来缓解症状<br>• 如果您确诊病人所患疾病为肺炎，请直接进行预后（下一步）和治疗（第四页）

For peer review only

**Prognosis (Note only one option will apply)**			**病人预后 （只选一项）**		
Tick one	Most infections of this type are better by:		选一项打勾	此类感染一般情况下的好转时间是:	
	1. Acute upper respiratory tract infection	14 days		1.急性上呼吸道感染	14 天
	2. Acute tonsil inflammation / Tonsillitis / Pharyngitis	7-8 days		2. 急性化脓性扁桃体炎/ 扁桃体炎/咽炎	7-8 天
	3. Acute ear inflammation / Otitis Media	8 days		3. 急性耳朵炎症/中耳炎	8 天
	4. Acute sinusitis	14-21 days		4. 急性鼻窦炎	14-21 天
	5. Acute tracheal/ bronchial inflammation / Tracheobronchitis / Bronchitis / Lower respiratory tract infection	21 days		5. 急性气管/支气管炎症/急性气管支气管炎/支气管炎/下呼吸道感染	21 天
	6. Pneumonia	● 1 week: fever should have resolved<br>● 4 weeks: chest pain and sputum production should have substantially reduced<br>● 6 weeks: cough and breathlessness should have substantially reduced<br>● 3 months: most symptoms should have resolved but fatigue may still be present<br>● 6 months: most people will feel back to normal		6. 肺炎	• 1 周: 发烧应该已经解决了<br>• 4 周: 胸痛和痰的产生应该大幅减轻<br>• 6 周: 咳嗽和呼吸困难应该大幅减轻<br>• 3 个月: 大多数症状应该已经解决, 但仍可能存在疲劳<br>• 6 个月: 大多数人都会感觉恢复正常

Page 4: treatment recommendations (Note only one option will apply – select correct option for your patient)

第四页：治疗建议（只选一项， 请为您的病人选择正确的治疗建议）

**Treatment recommendation for each type of RTI (only one type of RTI below will appear based on the diagnosis choice in page 3)**	**每类呼吸道疾病的治疗建议（根据您第三页的诊断结果，以下六种疾病的治疗建议只有一种会出现）**
1. Acute upper respiratory tract infection<br>• Treatment should prioritize targeting symptoms and supporting the body to recover<br>• Antibiotics are usually not to be used and can be harmful, potentially causing side effects that can be severe, and increasing the risk of future serious antibiotic resistant infections in the patient, their family and the community<br>• See table below for symptomatic treatment options (there are just suggestions that come from Chinese National Guidelines)	1. 急性上呼吸道感染<br>• 治疗应优先针对症状并支持身体恢复<br>• 抗生素通常不被使用。其可能有害，并且可能导致严重的副作用并增加患者，其家人和社区未来严重抗生素耐药性感染的风险<br>• 有关症状治疗方案，请参见下表一（表中为国家指南的建议，供参考）
2. Acute tonsil inflammation / Tonsillitis / Pharyngitis<br>• Treatment should prioritize targeting symptoms and supporting the body to recover<br>• Antibiotics are usually not to be used and can be harmful, potentially causing side effects that can be severe, and increasing the risk of future serious antibiotic resistant infections in the patient, their family and the community<br>• See table 1 below for symptomatic treatment options<br>• Consider using antibiotics when:<br>○ There is suppuration of tonsils<br>○ Temperature of >38.5°C	2. 急性扁桃体炎/扁桃体炎/咽炎<br>• 治疗应优先针对症状并支持身体恢复<br>• 抗生素通常不被使用。其可能有害，并且可能导致严重的副作用并增加患者、其家人和社区未来严重抗生素耐药性感染的风险<br>• 有关症状治疗方案，请参见表一<br>• 在下列情况下考虑使用抗生素<br>- 有扁桃体的化脓<br>- 体温> 38.5 °C
3. Acute ear inflammation / Otitis Media<br>• Antibiotics are usually not to be used and can be harmful, potentially causing side effects that can be severe, and increasing the risk of future serious antibiotic resistant infections in the patient, their family and the community<br>• See table 1 below for symptomatic treatment options<br>• Consider using antibiotics if one or more of these symptoms are present (although note that the first three symptoms are common in viral ear infections):<br>○ loss of hearing<br>○ elevated temperature<br>○ progressive swelling and hyperemia in the tympanic membrane<br>○ perforation of the tympanic membrane accompanied by yellow exudate	3. 急性耳朵炎症/中耳炎<br>• 抗生素通常不被使用。其可能有害，并且可能导致严重的副作用并增加患者、其家人和社区未来严重抗生素耐药性感染的风险<br>• 有关症状治疗方案，请参见表一<br>• 如果存在以下一种或多种症状，请考虑使用抗生素（尽管研究已表明前三种症状在病毒性耳朵炎症中也是常见的）：<br>- 丧失听力<br>- 体温升高

For peer review only - http://bmjopen.bmj.com/site/about/guidelines.xhtml

	- 鼓膜中的进行性肿胀和充血<br>- 鼓膜穿孔伴有黄色渗出物
4. Acute sinusitis<br>• Antibiotics are usually not to be used and can be harmful, potentially causing side effects that can be severe, and increasing the risk of future serious antibiotic resistant infections in the patient, their family and the community<br>• See table 1 below for symptomatic treatment options<br>• Consider using antibiotics if one or more of these symptoms are present:<br>o Symptoms for more than 10 days<br>o Discoloured or purulent nasal discharge<br>o Severe localised unilateral pain (particularly pain over teeth and jaw)<br>o Fever<br>o Marked deterioration after an initial milder phase	4. 急性鼻窦炎<br>• 抗生素通常不被使用。其可能有害，并且可能导致严重的副作用并增加患者、其家人和社区未来严重抗生素耐药性感染的风险<br>• 有关症状治疗方案，请参见表一<br>• 如果存在以下一种或多种症状，请考虑使用抗生素：<br>- 症状超过 10 天<br>- 褪色或化脓性鼻涕<br>- 严重的局部单侧疼痛（尤其是牙齿和下颌疼痛）<br>- 发烧<br>- 初始缓和阶段后出现明显恶化
5. Acute tracheal/ bronchial inflammation / Tracheobronchitis / Bronchitis / Lower respiratory tract infection<br>• Symptomatic treatment should be considered priority.<br>• Antibiotics should not be routinely used and can be harmful, potentially causing side effects that can be severe, and increasing the risk of future serious antibiotic resistant infections in the patient, their family and the community.<br>• Consider anti-microbial treatment in the following cases:<br>o patients over 75 years of age with an elevated temperature;<br>o patients with heart failure;<br>o patients with insulin-dependent diabetes;<br>o patients with severe diseases of the nervous system.	5. 急性气管/支气管炎症/气管支气管炎/支气管炎/下呼吸道感染<br>• 首先考虑对症治疗<br>• 抗生素不应常规使用。其可能有害，并且可能导致严重的副作用并增加患者、其家人和社区未来严重抗生素耐药性感染的风险<br>• 在以下情况下考虑抗生素治疗：<br>- 患者 75 岁以上并伴体温升高<br>- 心力衰竭患者<br>- 胰岛素依赖型糖尿病患者<br>- 患有严重神经系统疾病的患者
6. Pneumonia<br>• The decision for outpatient or inpatient treatment should be based on the severity of illness, and anti-infection therapy should be administered as early as possible.<br>• Patients with mild symptoms and normal GI function should receive treatment with oral medication<br>• Patients with heavy symptoms should receive IV treatment, and change to oral medication once there is clear improvement in clinical appearance and ability to receive oral medication.<br>o Young adults, patients with no underlying conditions	6. 肺炎<br>• 门诊或住院治疗取决于疾病的严重程度，并应尽早进行抗感染治疗<br>• 症状轻微且胃肠功能正常的患者应接受口服药物治疗<br>• 症状较重的患者应接受静脉注射治疗，一旦临床表现和接受口服药物的能力明显改善应改为口服药物治疗<br>- 年轻人，没有潜在疾病的患者

For peer review only

- Penicillin; Amoxicillin; Doxycycline, minocycline; 1st or 2nd gen cephalosporins; Respiratory quinolones (moxifloxacin, levofloxacin, and Gemifloxacin)
- The elderly or patients with underlying conditions
  - Monotherapeutic usage of 2nd gen cephalosporins (cefuroxime axetil, cefprozil, cefaclor etc.) or combined usage with macrolides; Monotherapeutic usage of amoxicillin/clavulanic acid or ampicillin/sulbactam or combined usage with macrolides; Respiratory quinolones

Referral criteria (they are suggestions from Chinese National Guidelines; they may not all be useful or relevant to you):

- Combined with other underlying illnesses, such as chronic cardiac insufficiency (III-IV)
- Immunosuppression
- Initial treatment is failed but the vital signs are stable
- Local or systemic complications, such as pulmonary abscess, but vital signs are stable
- Older than 65 years of age with underlying illnesses, and assessed as having the infection risk of ESBL and other multi-resistant strains
- CAP identification is not clear and requires further diagnosis

- 青霉素；阿莫西林；强力霉素，米诺环素；第一代或第二代头孢菌素；呼吸喹诺酮类药物（莫西沙星，左氧氟沙星和吉米沙星）
- 老年人或有潜在疾病的患者
  - 第二代头孢菌素（头孢呋辛酯，头孢丙烯，头孢克洛等）的单药治疗或与大环内酯类联合使用；阿莫西林/克拉维酸或氨苄青霉素/舒巴坦的单药治疗用途或与大环内酯联合使用；呼吸喹诺酮类

普通转诊（下列是国家指南的建议；以下建议不一定全部有用或者符合您当时的情况）：

- 合并基础疾病较多，如慢性心功能不全 (Ⅲ。Ⅳ级)、慢性肾脏疾病 3 ~ 5 期、肝硬化失代偿、 糖尿病急症
- 免疫抑制宿主发生 CAP
- 初始治疗失败，生命体征稳定
- 出现局部或全身并发症，如脓胸、肺脓肿， 生命体征稳定
- 年龄 / >65 岁有基础疾病患者，评估有超广 谱β-内酰胺酶 (ESBL)菌等耐多药感染风险
- CAP 诊断尚未明确，需要进一步鉴别诊断

Table 1 Symptomatic treatment options for RTI

表一 呼吸道感染对症治疗措施

**Symptoms**	**Symptomatic treatment options**	**症状**	**对症治疗措施**
temperature<br>headache<br>all-over muscle pain<br>pain in any area	Antipyretic analgesics including:<br>• Paracetamol<br>• Aspirin<br>• Ibuprofen*	发烧<br>头痛<br>全身肌肉疼痛<br>身体任何部位疼痛	解热镇痛药，包括：<br>• 对乙酰氨基酚/扑热息痛<br>• 阿司匹林<br>• 布洛芬*
blocked nose<br>hyperemia<br>edema in the nasal mucosa<br>sore throat	Compound decongestant and anti-allergenic medicine including:<br>• Pseudoephedrine<br>• 1% ephedrine nasal spray	鼻塞<br>充血<br>鼻粘膜水肿	复方抗充血剂和抗过敏药物，包括：<br>• 伪麻黄碱<br>• 1%麻黄素喷雾

For peer review only

		嗓子疼	
frequent sneezing<br>continual runny nose	Compound decongestant and anti-allergenic medicine including:<br>• chlorphenamine maleate<br>• loratadine<br>• diphenhydramine	频繁打喷嚏<br>持续流鼻涕	复方抗充血剂和抗过敏药物，包括：<br>• 马来酸氯苯那敏<br>• 氯雷他定<br>• 苯海拉明
Cough	Cough suppressants including:<br>• dextromethorphan hydrobromide<br>• codeine	咳嗽	止咳药，包括<br>• 右美沙芬<br>• 可待因
‘Inflammation’	TCM preparations may be effective<br>• yinqiao tablets<br>• shuanghuanglian<br>• antiviral granules<br>• yuxingcao (fish leaf)	‘炎症’	以下中成药或中药成分可能对你有效，包括：<br>• 银翘片<br>• 双黄连<br>• 抗病毒颗粒<br>• 鱼腥草

*Current Chinese national guidelines recommend using ibuprofen for symptoms of temperature, headache, all-over muscle pain and pain in any area; however, there is some research to show that the use of ibuprofen is unlikely to help the symptoms and is associated with their progression. Recent study has suggested that advising the use of ibuprofen may be unhelpful in managing RTIs.

*目前国家指南建议将布洛芬应用于发烧，头痛等呼吸道感染症状的对症治疗中。然而一些国外研究已经发现布洛芬对于其症状缓解没有帮助甚至会加重病情，已有研究提出布洛芬在呼吸系统感染治疗中是没有帮助的。

**Advice for all patients with diagnosis 1-5**	**对于所患疾病为 1-5 的病人的建议：**
The best way is to support your own body’s immune system to fight off the infection by, for example:<br>·Drink fluid (preferably water) at least 6 to 8 glasses of every day, particularly if you also have a fever;<br>·Keep warm and get plenty of rest;<br>·Eat a healthy, balanced diet containing plenty of fruit and vegetables.	最好的方法就是提高你自身的免疫力以预防感染，例如<br>• 每天至少喝 6 到 8 杯水（首选白开水），特别是发热的时候<br>• 保暖，多休息<br>• 健康均衡饮食，多吃水果蔬菜

**Things you can say to patients to help them understand that they do not need antibiotics**	**你可以给与病人以下建议，来帮助他们明白他们并不需要抗生素**
• Scientific research has found that antibiotics (e.g. Amoxicillin and Cephalosporin) do not help coughs, colds, sore throat or earache get better any faster;	• 科学研究发现抗生素（例如阿莫西林和头孢）并不能帮助咳嗽，感冒，嗓子疼，耳朵疼更快地好转
• Research has shown that in the past we took lots of antibiotics, such as Amoxicillin and Cephalosporin that we did not need	• 研究表明，在过去我们服用了很多我们不需要的抗生素，例如阿莫西林和头孢
• Taking antibiotics (e.g. Amoxicillin and Cephalosporin) may weaken your own body's methods for keeping you healthy, by killing the good bacteria in your gut.	• 服用抗生素（例如阿莫西林和头孢）会杀死肠道中的有益细菌，从而削弱身体保持健康的能力
• Taking antibiotics (e.g. Amoxicillin and Cephalosporin) for common infections now means that in the future antibiotics will work less well or not at all for you and your family	• 现在对常见的感染就使用抗生素（例如阿莫西林和头孢），意味着在未来，抗生素对你和你的家人的作用会更差或者根本不起作用
• Antibiotics can cause side effects (such as diarrhoea, rashes, feeling sick), which in rare cases can be very severe.	• 抗生素可以引起副作用(如腹泻、皮疹、恶心等)，在极少数情况下可能会非常严重

For peer review only

Page 5: Safety netting advice (when to come back to see the doctor)

第五页：复诊建议（何时再来看医生）

Finally, in the event of the following, please come back for treatment right away:	最后，如果出现以下现象请立即复诊:
. If your skin is very cold or has a strange colour, or you develop an unusual rash	• 如果你皮肤温度很低或者出现奇怪的颜色，或者出现异样皮疹
. If you feel confused have slurred speech, or become drowsy	• 出现头脑不清、言语不清、瞌睡
. If you have difficulty breathing including:<br>. hyperventilating<br>. your lips become purple<br>. skin between or above the ribs getting sucked or pulled in with every breath	• 出现呼吸困难，包括:<br>- 呼吸急促<br>- 嘴唇发紫<br>- 肋骨之间或之上的皮肤每次呼吸都会被吸入或拉入
. If you have a severe headache and are sick	• 出现严重头痛并感到恶心
. If you have a tight chest, chest pain,	• 出现胸闷、胸痛
. If you have difficulty swallowing or are drooling	• 出现吞咽困难或流口水
. If you cough up blood	• 出现咳血
. If you are feeling a lot worse	• 如果你感觉越来越差了
. If there is no improvement within (only one option will apply) – please note that most symptoms will be better before the listed length, but some can take this long and still get better without treatment<br>1. Acute upper respiratory tract infection – 14 days<br>2. Acute Tonsillitis / Pharyngitis – 8 days<br>3. Acute ear inflammation / Otitis Media – 8 days<br>4. Acute sinusitis – 21 days<br>5. Acute Tracheobronchitis – 21 days<br>6. Pneumonia – 1 week after treatment started	• 如果在正常恢复期内没有改善（每位患者只选一项）-请注意，大多数症状会提前好转，但是有些仍可能会持续到以下时间直至在没有治疗的情况下好转<br>1.急性上呼吸道感染 - 14 天<br>2.急性扁桃体炎/咽炎 - 8 天<br>3.急性耳朵炎症/中耳炎 - 8 天<br>4.急性鼻窦炎 - 21 天<br>5.急性气管支气管炎 - 21 天<br>6. 肺炎 - 已经接受治疗 1 周以后

For peer review only

Participant ID: ___________________

WP1 Clinical consultation doctor questionnaire

These questions must be answered by the **doctor** or by direct observation by the field worker

这部分问题要求**医生**回答或者通过调查员的观察完成 Consultation start time: __ h __ min (24-h clock)

开始接诊时间 ______________时________分（24 小时制）

1. Whether practitioner has checked with patient's symptoms? 医生是否询问症状

☐ Yes 是
☐ No 否

1.1 What are the patient's symptoms? 病人当前有何症状?

☐ Blocked/ runny nose	☐ 鼻塞/流涕
☐ Blocked nose<br>☐ Runny nose (clear/watery discharge)<br>☐ Snotty nose (yellow/green discharge)	☐ 鼻子不通<br>☐ 流清水鼻涕<br>☐ 流脓鼻涕 (黄/绿色鼻涕)
☐ Coughing	☐ 咳嗽
☐ Dry cough<br>☐ Cough with white sputum<br>☐ Cough with yellow/green sputum	☐ 干咳<br>☐ 咳嗽带白色痰<br>☐ 咳嗽带黄/绿色脓痰
☐ Throat problems	☐ 嗓子不舒服
☐ Sore throat<br>☐ Hyperemia/ swelling in throat<br>☐ Hyperemia/ enlargement of tonsils<br>☐ Pus on the tonsils<br>☐ Swollen lymph glands	☐ 咽痛<br>☐ 咽喉部充血/水肿<br>☐ 扁桃体充血/肿大<br>☐ 扁桃体化脓<br>☐ 下颌淋巴结肿大
☐ Breathing	☐ 呼吸
☐ Short of breath<br>☐ Tight chest<br>☐ Difficulty breathing<br>☐ Must sit erect to breathe<br>☐ Wet crackling in lungs	☐ 气促<br>☐ 胸闷<br>☐ 呼吸不畅/困难<br>☐ 端坐呼吸<br>☐ 肺部湿罗音
☐ Ear symptoms	☐ 耳部症状
☐ Blocked ears<br>☐ Tinnitus<br>☐ Pus/ fluid secretion<br>☐ Earache<br>☐ Loss of hearing	☐ 耳闷<br>☐ 耳鸣<br>☐ 流脓/液<br>☐ 耳痛<br>☐ 听力下降
☐ Fever	☐ 发热

For peer review only

Participant ID: ________________

☐ Low 37.3-38 ºC<br>☐ Moderate 38.1-39 ºC<br>☐ High >39 ºC	☐ 低热 37.3-38 摄氏度<br>☐ 中等度热 38.1-39 摄氏度<br>☐ 高热 39 摄氏度以上
☐ Pain	☐ 疼痛
☐ Headache<br>☐ Aching all-over<br>☐ Chest pain	☐ 头痛<br>☐ 全身酸痛<br>☐ 胸痛

Any other symptoms? If yes please state ______________________

除了以上症状，你还有其他不舒服的症状么？ ____________________________

1.2 What kinds of tests were ordered by practitioners? (Please tick and/or write down) （医生给病人开具了哪些检查，请选择或具体填写）

☐Blood test （血液检查）

☐X-Ray （X 射线）

☐CT （CT）

☐B Ultrasound (超声波诊断，B 超)

☐ECG （心电图检查）

☐None （没有）

Other (Please Specify) （其他的，请描述） _______________________

1.3 What kinds of tests were carried out by patient? (Please tick and/or write down) （病人实际做了哪些检查，请选择或具体填写）

☐Blood test （血液检查）

☐X-Ray （X 射线）

☐CT （CT）

☐B Ultrasound (超声波诊断，B 超)

☐ECG （心电图检查）

☐None （没有）

Other (Please Specify) _ （其他的，请描述） ______________________

1.4 Test results （检查结果）

Record practitioners' statement about test results as told to the patient, in his/her own words. If no test result is stated, then write down 'not stated'. 如果医生有告知病人的检查结果，请原话记录医生的描述。如果检查结果没有告知，请填写"未告知"。

____

For peer review only

Participant ID: ___________________

Diagnosis & Treatment 诊断和治疗

2. Whether practitioner has informed patient of the diagnosis?医生是否告知患者诊断

☐ Yes 是
☐ No 否

Tick one 选一种	**2.1 What is the diagnosis? (ask doctor to pick closest category from list of 6 RTI)**	**2.1 诊断是什么？（请医生在六类呼吸系统感染中选择最接近诊断结果的一项）**
	1. Acute upper respiratory tract infection including:<br>. Infectious cold / Acute rhinitis / upper respiratory tract catarrh<br>. Acute inflammation of the throat<br>. Acute viral pharyngitis<br>. Acute viral laryngitis	急性上呼吸道感染:<br>· 传染性感冒/急性鼻炎/上呼吸道粘膜炎<br>· 喉咙急性炎症<br>· 急性病毒性咽炎<br>· 急性病毒性喉炎
	2. Acute tonsil inflammation / Tonsillitis / Pharyngitis	急性化脓性扁桃体炎/ 扁桃体炎/咽炎
	3. Acute ear inflammation / Otitis Media	急性耳朵炎症/中耳炎
	4. Acute sinusitis	急性鼻窦炎
	5. Acute tracheal/ bronchial inflammation / Acute Tracheobronchitis / Bronchitis / Lower respiratory tract infection	急性气管/支气管炎症/急性气管支气管炎/支气管炎/下呼吸道感染
	6. Pneumonia	肺炎
	. Other please state [free text box]	其他请说明[自由文本框]

**2.2 One a scale of 0 to 10, how sick do you think this patient is? (where 10 is very sick and 0 is not sick)**	**2.2 在 1 到 6 的程度中，你认为这位病人有多不舒服？(6 表示非常不舒服， 1 表示一点点不舒服)**
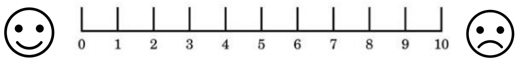	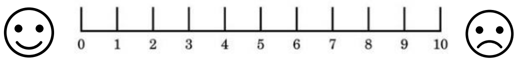

Whether practitioner has informed patient with medication? 医生是否告知患者用药情况

☐ Yes 是
☐ No 否

**2.3 Were any antibiotics prescribed?**	**2.3 抗生素是否开具?**
☐ Yes<br>☐ No	☐ 是<br>☐ 否

For peer review only

Participant ID: ___________________

If Yes, first tick the method of drug administration; then specify the name, total quantity and dosage in the table	如果是，请先勾选抗生素的给药方式；再在下表中具体说明药物通用名，总量和剂量
☐ IV Antibiotics prescribed<br>☐ Oral antibiotics prescribed	☐ 静脉注射抗生素<br>☐ 口服抗生素

2.4 Whether practitioner has informed patient with risks of antibiotics treatment 是否告知患者使用抗菌药物的危害

☐ Yes 告知
☐ No 未告知

IV Antibiotics prescribed:

Tick if prescribed	Medicine Name	Specification	Total quantity	Dosage
	Penicillin			
	Levofloxacin			
	Ampicillin			
	Clindamycin			
	Amikacin			
	Azithromycin			
	Metronidazole			
	Fosfomycin sodium			
	Amoxicillin			
	Erythromycin			
	Norfloxacin			
	Cefradine			

静脉注射抗生素：

如果有，请勾选	药物名称	规格	总量	剂量
	青霉素			
	左氧氟沙星			
	氨苄青霉素			
	克林霉素			
	阿米卡星(丁胺卡那霉素)			
	阿奇霉素			
	甲硝唑			
	磷霉素钠			
	阿莫西林			
	红霉素			

For peer review only

Participant ID: ___________________

	诺氟沙星			
	头孢拉定			

Oral antibiotics prescribed:

Tick if prescribed	Antibiotics Name	Specification	Total quantity	Dosage
	Amoxicillin			
	Erythromycin			
	Norfloxacin			
	Cefradine			

口服抗生素：

如果有，请勾选	药物名称	规格	总量	剂量
	阿莫西林			
	红霉素			
	诺氟沙星			
	头孢拉定			

Other medicines prescribed:

Tick if prescribed	Medicine Name	Specification	Total quantity	Dosage	Mode of administration
	Dexamethasone				
	Tetracaine				
	chlorpheniramine				
	Aminophylline				
	Ribavirin				
	Compound paracetamol amine capsules				
	Ambroxol				
	Asthma-calming and cough-stopping patch (*pingchuan zhike pian*)				
	Qing kailing(qinghai ling)				
	Galculus Bovis and Metronidazole Capsules				

For peer review only

Participant ID: ___________________

	(*rengong niuhuang jia xiaocuo jiaonang*:				

其它开具的药物:

如果有，请勾选	药物名称	规格	总数量	剂量	服药方式（吊水/口服/贴等）
	地塞米松				
	丁卡因				
	扑尔敏				
	氨茶碱				
	利巴韦林				
	复方氨酚胶囊				
	盐酸氨溴索				
	平喘止咳贴片				
	清开灵				
	人工牛黄甲硝唑胶囊				

**2.4 Did the patient ask for any of these treatments?**	**2.4 患者是否主动要求过以下治疗?**
☐ IV	☐ 输液
☐ Anti-inflammation medicine/antibiotics	☐ 抗生素/消炎药
☐ Other medicines	☐ 其他药物

Any further observation note of the consultation process (Researcher's observation):	其它有关诊疗咨询过程的观察记录?

For peer review only

Participant ID: ________________

Consultation end time: ___ h ___min (24-h clock)

结束接诊时间 _____________时_______分_（24 小时制）

Whether practitioner has informed patient with standard precautions in health care ? 医生是否向患者做疾病注意事项相关宣教

- ☐ Yes 是
- ☐ No 否

Whether practitioner has informed patient with standard precautions in infection control? 医生是否想患者做预防感染相关宣教?

- ☐ Yes 是
- ☐ No 否

For peer review only

Participant ID: ________________

WP1 Clinical consultation patient questionnaire

A field worker will administer this questionnaire at the clinical consultation at which the patient is recruited at both base line and follow up audit.

These questions must be answered by the **patient** or by direct observation by the field worker

这部分问题要求**病人**回答或者通过调查员的观察完成

1. Eligibility criteria 入选条件

Does patient have a suspected RTI?<br>. Yes<br>. No – exclude from study	病人是否疑似呼吸系统感染?<br>· 是<br>· 否—从本研究中排除
Is patient aged 18 years or older?<br>. Yes<br>. No – exclude from study	病人是否年满 18 岁?<br>· 是<br>· 否—从本研究中排除
Is today the first time the patient has sought treatment for this this illness or a repeat visit?<br>. First time consultation<br>. Repeat consultation – exclude from study	今天是病人首次还是再次为这个病来这里就诊?<br>· 首次就诊<br>· 再次就诊—从本研究中排除

Date completed: _________	调查日期: ______年____月____日
Name of field worker completing proforma: _________	调查员姓名: _______________

For peer review only

Participant ID: ________________

2. Participant Characteristics 研究对象基本情况

**2.1 Gender**<br>☐ Female<br>☐ Male	**2.1 性别:**<br>☐ 女性<br>☐ 男性
**2.2 Date of Birth (Year/Month/Day)**	**2.2 生日**<br>______年____月____日
**2.3 How many years of education have you completed?**<br>________________years<br>*[Write down the exact number]*	**2.3 你上了几年学?**<br>______年<br>(写下确切的数字)

**2.4 Where do you live?**<br>*Name of town or village:*<br>_____________________________________	**2.4 你住在哪里?**<br>乡镇/村的名字<br>_______________________________
**2.5 If you have lived outside your usual home residence for more than a month over the past 12 months, how many months have you been away?**<br>________months	**2.5 在过去一年，如果你离开家在外生活超过 1 个月，请给出你具体离家几个月?**<br>________个月
**2.6Is the place where you have been living urban or rural?**<br>☐ Urban<br>☐ Rural<br>☐ Not applicable	**2.7 你在外生活的地方是农村还是城市**<br>☐ 城市<br>☐ 农村<br>☐ 不适用
**2.7What is your Household registration status (*hukou*) for accessing health care benefits?**<br>☐ Urban<br>☐ Rural<br>☐ Other____________________	**2.8 目前跟你医保挂钩的是什么户口?**<br>☐ 城市<br>☐ 农村<br>☐ 其他____________________
**2.8What type of insurance do you have, if any?**<br>☐ New rural cooperative Medicalinsurance scheme<br>☐ Urban Employee's Medical Insurance<br>☐ Urban Residents' Medical Insurance<br>☐ Not Having Medical Insurance<br>☐ Other Type of Insurance *[please specify]*:<br>__________________________	**2.9 你参加了哪些医疗保险?**<br>☐ 新型农村合作医疗<br>☐ 城镇职工医疗保险<br>☐ 城镇居民医疗保险<br>☐ 没有医疗保险<br>☐ 其他医疗保险（请注明）：<br>_________________________

For peer review only

Participant ID: ___________________

3. Duration of illness and the severity 患病时长和严重程度

**Duration of your illness: ______ days**	**你的病已经持续了________天**
**One a scale of 0 to 10, how sick do you feel? (where 10 is very sick and 0 is not sick)**	**在 0 到 10 的程度中，你认为你有多不舒服？(10 表示非常不舒服， 0 表示没有不舒服)**
☺ 0 1 2 3 4 5 6 7 8 9 10 ☹	☺ 0 1 2 3 4 5 6 7 8 9 10 ☹

Only complete these questions if the symptoms have not been recorded on the clinician questionnaire.

请仅在医生问卷没有收集到下列症状的情况下，向病人询问下列问题。

What are yoursymptoms?您当前有何症状?

☐Blocked/ runny nose	☐鼻塞/流涕
☐ Blocked nose<br>☐ Runny nose (clear/watery discharge)<br>☐ Snotty nose (yellow/green discharge)	☐ 鼻子不通<br>☐ 流清水鼻涕<br>☐ 流脓鼻涕(黄/绿色鼻涕)
☐Coughing	☐咳嗽
☐ Dry cough<br>☐ Cough with white sputum<br>☐ Cough with yellow/green sputum	☐ 干咳<br>☐ 咳嗽带白色痰<br>☐ 咳嗽带黄/绿色脓痰
☐Throat problems	☐嗓子不舒服
☐Breathing	☐呼吸
☐ Short of breath<br>☐ Tight chest<br>☐ Difficulty breathing<br>☐ Must sit erect to breathe	☐ 气促<br>☐ 胸闷<br>☐ 呼吸不畅/困难<br>☐ 端坐呼吸<br>☐
☐Earsymptoms	☐耳部症状
☐ Blocked ears<br>☐ Tinnitus<br>☐ Pus/ fluid secretion<br>☐ Earache<br>☐ Loss of hearing	☐ 耳闷<br>☐ 耳鸣<br>☐ 流脓/液<br>☐ 耳痛<br>☐ 听力下降
☐Fever	☐发热
☐Pain	☐疼痛
☐ Headache<br>☐ Aching all-over<br>☐ Chest pain	☐ 头痛<br>☐ 全身酸痛<br>☐ 胸痛

For peer review only

Participant ID: __________________

除了以上症状，你还有其他不舒服的症状么？ ______________________________

Alongside above symptoms, did you have any other symptoms that make you unwell? ______

4. Medication （用药情况）

1	2	3	4	5
**Have you taken any medication for this illness before coming here? Yes/No** （在为这个病来就诊前，你有服用任何药物么？<br><br>**If yes, please answer column 2 and 3**<br>如果回答　是，　请回答问题 2 和 3<br><br>**If No, please answer column 4 and 5**<br><br>如果回答　没有，请回答问题 4 和 5	**Days have you taken it?**<br>（你服用药物几天？）	**Where did you got it?**<br>（你从那里获得服用的药物？）<br>1, pharmacies outside the hospitals（医院外的药房）<br>2, family or relatives or friends （家人和朋友给的）<br>3 Residual drugs at home （之前用药剩下的）	**Have you had the medication in the past six month before this illness?**<br>（在这个病的前 6 个月里，你有服用任何药物么？<br>**Yes/No　是/**否<br>**If yes, please answer column 5**<br>（回答是，请继续回答问题 5）	**How many course have you taken?**<br>（你服用几个疗程的药物）<br><br>1,1-3 times<br>1-3 个疗程<br>2, 4-6 times<br>4-6 个疗程<br>3, 6 times<br>6 个疗程<br>4, more than 6 times<br>超过 6 个疗程

For peer review only

5.Patient Satisfaction 患者满意度

One a scale of 0 to 10, how satisfied were you with your visit to the doctor today? (where 10 is very satisfied and 0 is not satisfied at all)	在 0 到 10 的程度中，你今天在这里看病满意吗？(10 表示非常满意， 0 表示完全不满意)
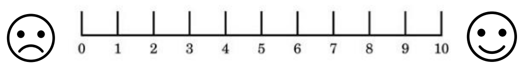	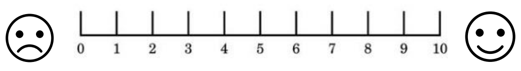

Participant ID: __________________

Health Questionnaire

English version for the UK

VERSION FOR INTERVIEWER ADMINISTRATION

Note to interviewer: although allowance should be made for the interviewer's particular style of speaking, the wording of the questionnaire instructions should be followed as closely as possible. In the case of the EQ-5D-5L descriptive system on page 2 of the questionnaire, the precise wording must be followed.

If the respondent has difficulty choosing a response, or asks for clarification, the interviewer should repeat the question word for word and ask the respondent to answer in a way that most closely resembles his or her thoughts about his or her health today.

INTRODUCTION

(Note to interviewer: please read the following to the respondent.)

We are trying to find out what you think about your health. I will explain what to do as I go along, but please interrupt me if you do not understand something or if things are not clear to you. There are no right or wrong answers. We are interested only in your personal view.

First, I am going to read out some questions. Each question has a choice of five answers. Please tell me which answer best describes your health TODAY.

Do not choose more than one answer in each group of questions.

For peer review only

Participant ID: ___________________

(Note to interviewer: first read all five options for each question. Then ask the respondent to choose which one applies to him/herself. Repeat the question and options if necessary. Mark the appropriate box under each heading. You may need to remind the respondent regularly that the timeframe is TODAY.)

EQ-5D DESCRIPTIVE SYSTEM

MOBILITY

First, I would like to ask you about mobility. Would you say that:

1. You have no problems in walking about? ❑
2. You have slight problems in walking about? ❑
3. You have moderate problems in walking about? ❑
4. You have severe problems in walking about? ❑
5. You are unable to walk about? ❑

SELF-CARE

Next, I would like to ask you about self-care. Would you say that:

1. You have no problems washing or dressing yourself? ❑
2. You have slight problems washing or dressing yourself? ❑
3. You have moderate problems washing or dressing yourself? ❑
4. You have severe problems washing or dressing yourself? ❑
5. You are unable to wash or dress yourself? ❑

USUAL ACTIVITIES

Next, I would like to ask you about usual activities, for example work, study, housework, family or leisure activities. Would you say that:

1. You have no problems doing your usual activities? ❑
2. You have slight problems doing your usual activities? ❑
3. You have moderate problems doing your usual activities? ❑
4. You have severe problems doing your usual activities? ❑
5. You are unable to do your usual activities? ❑

PAIN / DISCOMFORT

Next, I would like to ask you about pain or discomfort. Would you say that:

1. You have no pain or discomfort? ❑
2. You have slight pain or discomfort? ❑
3. You have moderate pain or discomfort? ❑

For peer review only

Participant ID: ___________________

4. You have severe pain or discomfort? ❑
5. You have extreme pain or discomfort? ❑

The best health you can imagine

ANXIETY / DEPRESSION

Finally, I would like to ask you about anxiety or depression. Would you say that:

1. You are not anxious or depressed? ❑
2. You are slightly anxious or depressed? ❑
3. You are moderately anxious or depressed? ❑
4. You are severely anxious or depressed? ❑
5. You are extremely anxious or depressed? ❑

For peer review only

EQ-5D VAS

- **Now, I would like to ask you to say how good or bad your health is TODAY.**

- **I would like you to try to picture in your mind a scale that looks like a thermometer.**
  (Note to interviewer: if interviewing face-to-face, please show the person the VAS scale.)

- **The best health you can imagine is marked 100 (one hundred) at the top of the scale and the worst health you can imagine is marked 0 (zero) at the bottom.**

- **I would now like you to tell me the point on this scale where you would put your health TODAY.**
  (Note to interviewer: mark the scale at the point indicating the respondent's 'health today'. Now, please write the number you marked on the scale in the box below.)

Participant ID: ___________________

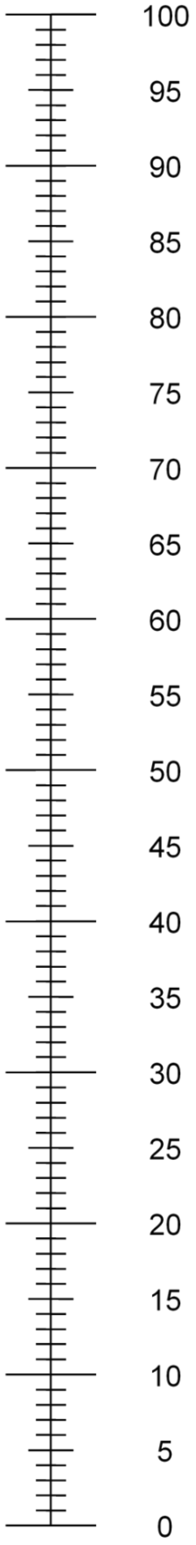

THE RESPONDENT'S HEALTH TODAY = ☐

Thank you for taking the time to answer these questions.

The worst
health you can
imagine

For peer review only

WP1 Patient telephone questionnaire – Day 7

A field worker will conduct phone interviews with the patients at 7, 14 and 21 days. Patients will also be asked the questions from the EQ5D.

调查员将在第 7， 14 和 21 天对病人进行电话访谈。除了以下问题，病人还会被问道一些 EQ5D 问卷中的问题。

1.Basic information 基本情况

**Patient ID**	病人编号
Days since baseline consultation<br>☐ 7 days<br>☐ 14 days<br>☐ 21 days	这是最初看病之后的第几天?<br>☐ 7 天<br>☐ 14 天<br>☐ 21 天
Date completed: __________	调查日期: ______年____月____日
Name of field worker completing proforma:<br>__________	调查员姓名: ________________

2. Continuing Illness Severity 疾病持续严重程度

**One a scale of 0 to 10, how sick do you feel? (where 10 is very sick and 0 is not sick)**	在 **0** 到 **10** 的程度中，你认为你有多不舒服？ **(10** 表示非常不舒服， **0** 表示没有不舒服**)**
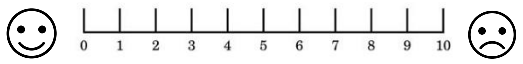 	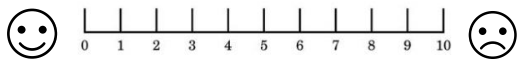

3. Patient view of leaflet 病人对宣传手册的看法

Did you have a look of the patient leaflet?<br>☐ Yes<br>☐ No<br>☐ Did not get leaflet	你是否看了病人宣传手册?<br>☐ 是<br>☐ 否<br>☐ 没有拿到
How easy was the patient leaflet to understand?<br>☐ Very easy, I understood all of it<br>☐ Easy, I understood most of it<br>☐ Not Easy, I only undersood a little<br>☐ Too hard, I did not understand any<br>☐ I am not able to read	你觉得病人宣传手册有多容易理解?<br>☐ 非常容易，我可以全部理解<br>☐ 容易，我可以理解大部分<br>☐ 不容易，我只能理解小部分<br>☐ 非常难，我完全不能理解<br>☐ 我读不了宣传手册

For peer review only

For peer review only - http://bmjopen.bmj.com/site/about/guidelines.xhtml

Did the leaflet change your ideas or beliefs about what illnesses need anti-inflammation medicine/ antibiotics?<br>☐ Yes<br>☐ No	病人宣传手册对您关于什么样的<br>疾病需要使用消炎药/抗生素的想法有改变吗?<br>☐ 有<br>☐ 没有

For peer review only

4. Antibiotic Consumption 抗生素服用情况

Were you prescribed oral anti-bacterial/anti-inflammation medicine in your baseline consultation?<br>☐ Yes (if yes, field worker will ask the two questions below in this table)<br>☐ No<br>☐ Not applicable	您在最初看病时是否有开口服抗菌药物/消炎药吗？<br>☐ 是（如果有，请继续问下面两个问题）<br>☐ 否<br>☐ 不清楚
How many days and times per day did you actually take a dose since you saw the Dr?<br>☐ [Number] ________of days<br>☐ [Number]________ times / day	从那之后你已经吃了多少天？每天实际吃多少次？<br>☐ （ ）天<br>☐ （ ）次/天
What was the dose did you take every time?	您每次吃的剂量是多少？
Have you stopped taking the anti-bacterial/anti-inflammation medicine that you were prescribed?<br>☐ Yes<br>☐ No<br>☐ Not applicable	你是否已经停止服用给你开的抗菌药物/消炎药了？<br>☐ 是<br>☐ 否<br>☐ 不清楚

For peer review only

For peer review only - http://bmjopen.bmj.com/site/about/guidelines.xhtml

EQ-5D-5L

Health Questionnaire

English version for the UK

VERSION FOR INTERVIEWER ADMINISTRATION

Note to interviewer: although allowance should be made for the interviewer's particular style of speaking, the wording of the questionnaire instructions should be followed as closely as possible. In the case of the EQ-5D-5L descriptive system on page 2 of the questionnaire, the precise wording must be followed.

If the respondent has difficulty choosing a response, or asks for clarification, the interviewer should repeat the question word for word and ask the respondent to answer in a way that most closely resembles his or her thoughts about his or her health today.

INTRODUCTION

(Note to interviewer: please read the following to the respondent.)

We are trying to find out what you think about your health. I will explain what to do as I go along, but please interrupt me if you do not understand something or if things are not clear to you. There are no right or wrong answers. We are interested only in your personal view.

First, I am going to read out some questions. Each question has a choice of five answers. Please tell me which answer best describes your health TODAY.

Do not choose more than one answer in each group of questions.

(Note to interviewer: first read all five options for each question. Then ask the respondent to choose which one applies to him/herself. Repeat the question and options if necessary. Mark the appropriate box under each heading. You may need to remind the respondent regularly that the timeframe is TODAY.)

For peer review only - http://bmjopen.bmj.com/site/about/guidelines.xhtml

EQ-5D DESCRIPTIVE SYSTEM

MOBILITY

First, I would like to ask you about mobility. Would you say that:

1. You have no problems in walking about? ❑
2. You have slight problems in walking about? ❑
3. You have moderate problems in walking about? ❑
4. You have severe problems in walking about? ❑
5. You are unable to walk about? ❑

SELF-CARE

Next, I would like to ask you about self-care. Would you say that:

1. You have no problems washing or dressing yourself? ❑
2. You have slight problems washing or dressing yourself? ❑
3. You have moderate problems washing or dressing yourself? ❑
4. You have severe problems washing or dressing yourself? ❑
5. You are unable to wash or dress yourself? ❑

USUAL ACTIVITIES

Next, I would like to ask you about usual activities, for example work, study, housework, family or leisure activities. Would you say that:

1. You have no problems doing your usual activities? ❑
2. You have slight problems doing your usual activities? ❑
3. You have moderate problems doing your usual activities? ❑
4. You have severe problems doing your usual activities? ❑
5. You are unable to do your usual activities? ❑

PAIN / DISCOMFORT

Next, I would like to ask you about pain or discomfort. Would you say that:

1. You have no pain or discomfort? ❑
2. You have slight pain or discomfort? ❑
3. You have moderate pain or discomfort? ❑
4. You have severe pain or discomfort? ❑
5. You have extreme pain or discomfort? ❑

ANXIETY / DEPRESSION

Finally, I would like to ask you about anxiety or depression. Would you say that:

1. You are not anxious or depressed? ❑
2. You are slightly anxious or depressed? ❑
3. You are moderately anxious or depressed? ❑
4. You are severely anxious or depressed? ❑
5. You are extremely anxious or depressed? ❑

For peer review only - http://bmjopen.bmj.com/site/about/guidelines.xhtml

EQ-5D VAS

- **Now, I would like to ask you to say how good or bad your health is TODAY.**

- **I would like you to try to picture in your mind a scale that looks like a thermometer.**
(Note to interviewer: if interviewing face-to-face, please show the person the VAS scale.)

- **The best health you can imagine is marked 100 (one hundred) at the top of the scale and the worst health you can imagine is marked 0 (zero) at the bottom.**

- **I would now like you to tell me the point on this scale where you would put your health TODAY.**
(Note to interviewer: mark the scale at the point indicating the respondent's 'health today'. Now, please write the number you marked on the scale in the box below.)

THE RESPONDENT'S HEALTH TODAY =

Thank you for taking the time to answer these questions.

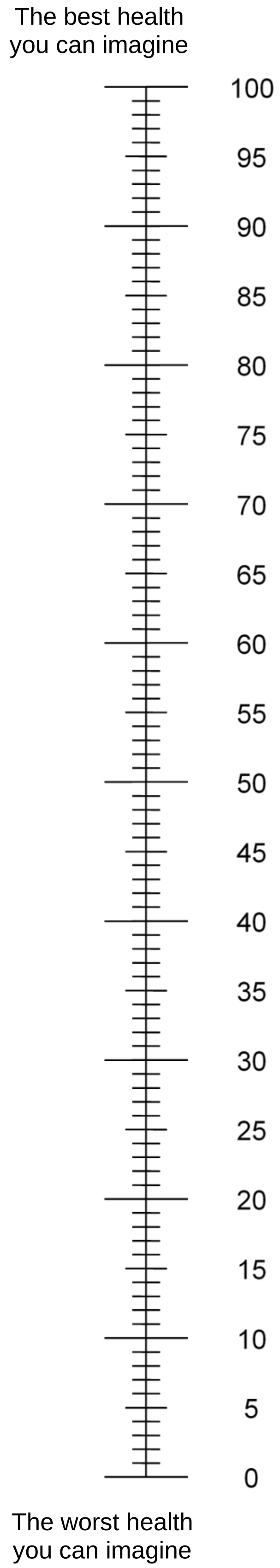

For peer review only

WP1 Patient telephone questionnaire – Day 14

A field worker will conduct phone interviews with the patients at 7, 14 and 21 days. Patients will also be asked the questions from the EQ5D.

调查员将在第 7， 14 和 21 天对病人进行电话访谈。除了以下问题，病人还会被问道一些 EQ5D 问卷中的问题。

1.Basic information 基本情况

**Patient ID**	**病人编号**
Days since baseline consultation<br>☐ 7 days<br>☐ 14 days<br>☐ 21 days	这是最初看病之后的第几天？<br>☐ 7 天<br>☐ 14 天<br>☐ 21 天
Date completed: __________	调查日期： ______年____月____日
Name of field worker completing proforma:<br>__________	调查员姓名： _______________

2. Continuing Illness Severity 疾病持续严重程度

**One a scale of 0 to 10, how sick do you feel? (where 10 is very sick and 0 is not sick)**	**在 0 到 10 的程度中，你认为你有多不舒服？（10 表示非常不舒服， 0 表示没有不舒服）**
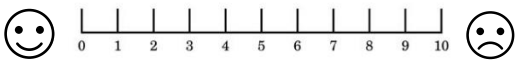	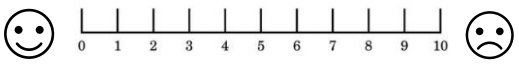

3. Antibiotic Consumption 抗生素服用情况

Were you prescribed oral anti-bacterial/anti-inflammation medicine in your baseline consultation?<br>☐ Yes (if yes, field worker will ask the two questions below in this table)<br>☐ No<br>☐ Not applicable	您在最初看病时是否有开口服抗菌药物/消炎药吗？<br>☐ 是（如果有，请继续问下面两个问题）<br>☐ 否<br>☐ 不清楚
How many days and times per day did you actually take a dose since you saw the Dr?<br>☐ [Number] _________of days<br>☐ [Number]_________ times / day	从那之后你已经吃了多少天？每天实际吃多少次？<br>☐ （ ） 天<br>☐ （ ） 次/天
What was the dose did you take every time?	您每次吃的剂量是多少？

For peer review only - http://bmjopen.bmj.com/site/about/guidelines.xhtml

Have you stopped taking the anti-bacterial/anti-inflammation medicine that you were prescribed?<br><br>☐ Yes<br>☐ No<br>☐ Not applicable	你是否已经停止服用给你开的抗菌药物/消炎药了？<br><br>☐ 是<br>☐ 否<br>☐ 不清楚

For peer review only - http://bmjopen.bmj.com/site/about/guidelines.xhtml

Health Questionnaire

English version for the UK

VERSION FOR INTERVIEWER ADMINISTRATION

Note to interviewer: although allowance should be made for the interviewer's particular style of speaking, the wording of the questionnaire instructions should be followed as closely as possible. In the case of the EQ-5D-5L descriptive system on page 2 of the questionnaire, the precise wording must be followed.

If the respondent has difficulty choosing a response, or asks for clarification, the interviewer should repeat the question word for word and ask the respondent to answer in a way that most closely resembles his or her thoughts about his or her health today.

INTRODUCTION

(Note to interviewer: please read the following to the respondent.)

We are trying to find out what you think about your health. I will explain what to do as I go along, but please interrupt me if you do not understand something or if things are not clear to you. There are no right or wrong answers. We are interested only in your personal view.

First, I am going to read out some questions. Each question has a choice of five answers. Please tell me which answer best describes your health TODAY.

Do not choose more than one answer in each group of questions.

(Note to interviewer: first read all five options for each question. Then ask the respondent to choose which one applies to him/herself. Repeat the question and options if necessary. Mark the appropriate box under each heading. You may need to remind the respondent regularly that the timeframe is TODAY.)

For peer review only - http://bmjopen.bmj.com/site/about/guidelines.xhtml

EQ-5D DESCRIPTIVE SYSTEM

MOBILITY

First, I would like to ask you about mobility. Would you say that:

1. You have no problems in walking about? ❑
2. You have slight problems in walking about? ❑
3. You have moderate problems in walking about? ❑
4. You have severe problems in walking about? ❑
5. You are unable to walk about? ❑

SELF-CARE

Next, I would like to ask you about self-care. Would you say that:

1. You have no problems washing or dressing yourself? ❑
2. You have slight problems washing or dressing yourself? ❑
3. You have moderate problems washing or dressing yourself? ❑
4. You have severe problems washing or dressing yourself? ❑
5. You are unable to wash or dress yourself? ❑

USUAL ACTIVITIES

Next, I would like to ask you about usual activities, for example work, study, housework, family or leisure activities. Would you say that:

1. You have no problems doing your usual activities? ❑
2. You have slight problems doing your usual activities? ❑
3. You have moderate problems doing your usual activities? ❑
4. You have severe problems doing your usual activities? ❑
5. You are unable to do your usual activities? ❑

PAIN / DISCOMFORT

Next, I would like to ask you about pain or discomfort. Would you say that:

1. You have no pain or discomfort? ❑
2. You have slight pain or discomfort? ❑
3. You have moderate pain or discomfort? ❑
4. You have severe pain or discomfort? ❑
5. You have extreme pain or discomfort? ❑

ANXIETY / DEPRESSION

Finally, I would like to ask you about anxiety or depression. Would you say that:

1. You are not anxious or depressed? ❑
2. You are slightly anxious or depressed? ❑
3. You are moderately anxious or depressed? ❑
4. You are severely anxious or depressed? ❑
5. You are extremely anxious or depressed? ❑

For peer review only

EQ-5D VAS

- **Now, I would like to ask you to say how good or bad your health is TODAY.**
- **I would like you to try to picture in your mind a scale that looks like a thermometer.**
  (Note to interviewer: if interviewing face-to-face, please show the person the VAS scale.)
- **The best health you can imagine is marked 100 (one hundred) at the top of the scale and the worst health you can imagine is marked 0 (zero) at the bottom.**
- **I would now like you to tell me the point on this scale where you would put your health TODAY.**
  (Note to interviewer: mark the scale at the point indicating the respondent's 'health today'. Now, please write the number you marked on the scale in the box below.)

THE RESPONDENT'S HEALTH TODAY = ☐

Thank you for taking the time to answer these questions.

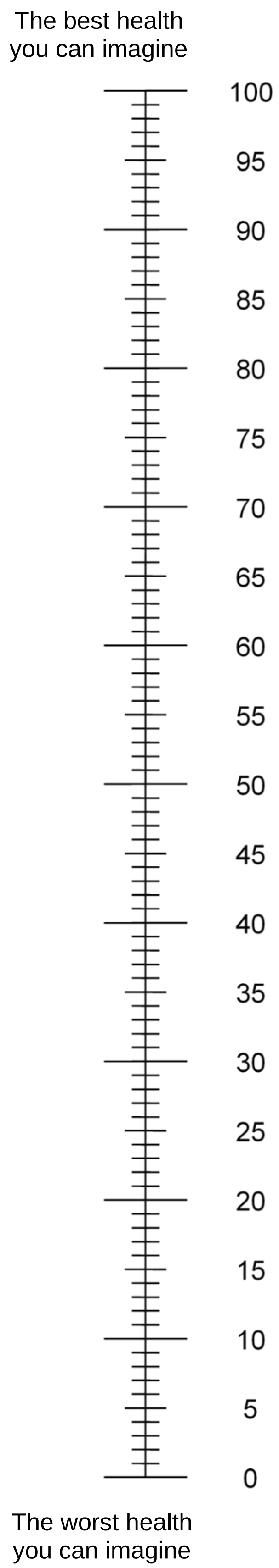

For peer review only

WP1 Patient telephone questionnaire – Day 21

A field worker will conduct phone interviews with the patients at 7, 14 and 21 days. Patients will also be asked the questions from the EQ5D.

调查员将在第 7，14 和 21 天对病人进行电话访谈。除了以下问题，病人还会被问道一些 EQ5D 问卷中的问题。

1.Basic information 基本情况

**Patient ID**	**病人编号**
Days since baseline consultation<br>☐ 7 days<br>☐ 14 days<br>☐ 21 days	这是最初看病之后的第几天?<br>☐ 7 天<br>☐ 14 天<br>☐ 21 天
Date completed: __________	调查日期：______年____月____日
Name of field worker completing proforma:<br>__________	调查员姓名：_______________

2. Continuing Illness Severity 疾病持续严重程度

**One a scale of 0 to 10, how sick do you feel? (where 10 is very sick and 0 is not sick)**	**在 0 到 10 的程度中，你认为你有多不舒服？（10 表示非常不舒服， 0 表示没有不舒服）**
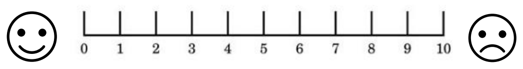	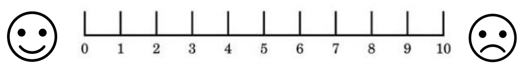

3. Antibiotic Consumption 抗生素服用情况

Were you prescribed oral anti-bacterial/anti-inflammation medicine in your baseline consultation?<br>☐ Yes (if yes, field worker will ask the two questions below in this table)<br>☐ No<br>☐ Not applicable	您在最初看病时是否有开口服抗菌药物/消炎药吗?<br>☐ 是（如果有，请继续问下面两个问题）<br>☐ 否<br>☐ 不清楚
How many days and times per day did you actually take a dose since you saw the Dr?<br>☐ [Number] _________of days<br>☐ [Number]_________ times / day	从那之后你已经吃了多少天？每天实际吃多少次?<br>☐ （ ）天<br>☐ （ ）次/天
Have you stopped taking the anti-	你是否已经停止服用给你开的抗菌药物/消炎

For peer review only

bacterial/anti-inflammation medicine that you were prescribed?<br>☐ Yes<br>☐ No<br>☐ Not applicable	药了？<br>☐ 是<br>☐ 否<br>☐ 不清楚

4. Resource Use for Economic Evaluation

Additional Health Care Utilisation 其他卫生服务的使用

Did you seek treatment for your illness from any of these since we talked to you in your consultation?	How many times did you visit there?	How much did you pay in total?	How much did you pay from your own pocket?
Pharmacy			
Other township health centre			
Village clinics			
Private clinics			

自从上次看病之后，你是否还因为你的病去下列地方寻医问药?	你去了几次?	总共花了多少钱?	你自费付了多少钱?
药店			
其他乡镇卫生院			
村卫生室			
个体诊所			

Did you seek secondary care for your illness since we talked to you in your consultation?	How many times did you visit?	How much did you pay in total	How much did you pay from your own pocket?
Outpatient			
Inpatient			
Accident and emergency			

自从上次看病之后，你是否因为你的病去医院就诊?	你去了几次?	总共花了多少钱?	你自费付了多少钱?
门诊			
住院			
急诊			

After the consultation when	Prescribed	Bought	Obtained from	How much	How much

For peer review only - http://bmjopen.bmj.com/site/about/guidelines.xhtml

we recruited you, have you had any treatment for this illness that wasn't prescribed in your consultation?	by other doctors	from pharmacy	other source (home, family, friends)	did you pay in total?	did you pay from your own pocket?
**Anti-bacterial/anti-inflammation medicine**					
☐ Amoxicillin	Y/N	Y/N	Y/N	¥	¥
☐ Erythromycin	Y/N	Y/N	Y/N	¥	¥
☐ Cefradine	Y/N	Y/N	Y/N	¥	¥
☐ Levofloxacin	Y/N	Y/N	Y/N	¥	¥
☐ Cefuroxime	Y/N	Y/N	Y/N	¥	¥
☐ Other anti-bacterial/anti-inflammation medicine [please specify]	Y/N	Y/N	Y/N	¥	¥
**Other medicines/treatment**					
☐ Paracetamol	Y/N	Y/N	Y/N	¥	¥
☐ Ganmaoling Granules （e.g. 999 cold remedy）	Y/N	Y/N	Y/N	¥	¥
☐ Qingkailing	Y/N	Y/N	Y/N	¥	¥
☐ Yinhuang Granules	Y/N	Y/N	Y/N	¥	¥
☐ Xiongju Shangqing	Y/N	Y/N	Y/N	¥	¥
☐ Lianhua (lotus) Qingwen capsules	Y/N	Y/N	Y/N	¥	¥
☐ Cough syrup	Y/N	Y/N	Y/N	¥	¥
☐ Ambroxol Hydrochloride oral suspension	Y/N	Y/N	Y/N	¥	¥
☐ Keteling	Y/N	Y/N	Y/N	¥	¥
☐ Compound liquorice tablets	Y/N	Y/N	Y/N	¥	¥
☐ Vitamin C	Y/N	Y/N	Y/N	¥	¥
☐ Crystal sugar snow pear	Y/N	Y/N	Y/N	¥	¥
☐ Ginger	Y/N	Y/N	Y/N	¥	¥
☐ Mugwort	Y/N	Y/N	Y/N	¥	¥
☐ Pipa (loquat)	Y/N	Y/N	Y/N	¥	¥
☐ Orange peel granules	Y/N	Y/N	Y/N	¥	¥
☐ Other [please specify]	Y/N	Y/N	Y/N	¥	¥

在最初看病之后，你是否自己用过了其他不是这次看病是所开具的药物？	其他医生开具	从药店购买	其他方法获得（家中储存，家人，朋友）	这些药总共多少钱？	你自费付了多少钱？
**抗菌药/消炎药**					
☐ 阿莫西林	是/否	是/否	是/否	¥	¥
☐ 红霉素	是/否	是/否	是/否	¥	¥
☐ 头孢拉定	是/否	是/否	是/否	¥	¥

For peer review only

☐ 左氧氟沙星	是/否	是/否	是/否	¥	¥
☐ 头孢呋辛	是/否	是/否	是/否	¥	¥
☐ 其他抗菌药/消炎药（请注明）	是/否	是/否	是/否	¥	¥
**其他药物**					
☐ 扑热息痛	是/否	是/否	是/否	¥	¥
☐ 感冒灵颗粒（如 999 感冒灵）	是/否	是/否	是/否	¥	¥
☐ 清开灵	是/否	是/否	是/否	¥	¥
☐ 银黄颗粒	是/否	是/否	是/否	¥	¥
☐ 芎菊上清	是/否	是/否	是/否	¥	¥
☐ 莲花清瘟胶囊	是/否	是/否	是/否	¥	¥
☐ 咳嗽糖浆	是/否	是/否	是/否	¥	¥
☐ 盐酸氨溴索口服液	是/否	是/否	是/否	¥	¥
☐ 咳特灵	是/否	是/否	是/否	¥	¥
☐ 复方甘草片	是/否	是/否	是/否	¥	¥
☐ 维生素 C	是/否	是/否	是/否	¥	¥
☐ 冰糖雪梨	是/否	是/否	是/否	¥	¥
☐ 姜	是/否	是/否	是/否	¥	¥
☐ 艾草	是/否	是/否	是/否	¥	¥
☐ 琵琶	是/否	是/否	是/否	¥	¥
☐ 陈皮颗粒	是/否	是/否	是/否	¥	¥
☐ 其他（请注明）	是/否	是/否	是/否	¥	¥

Productivity loss 生产力下降

**If you work, how many days in total have you taken off work due to this illness?**	如果你工作，那么因为这次生病你总共耽误了几天上班？
{number} days	（ ）天
**If you are in full time education, please state how many days in total have you missed your education?**	如果你是学生，那么因为这次生病你总共耽误了几天上学？
{number} days	（ ）天

For peer review only - http://bmjopen.bmj.com/site/about/guidelines.xhtml

Health Questionnaire

English version for the UK

VERSION FOR INTERVIEWER ADMINISTRATION

Note to interviewer: although allowance should be made for the interviewer's particular style of speaking, the wording of the questionnaire instructions should be followed as closely as possible. In the case of the EQ-5D-5L descriptive system on page 2 of the questionnaire, the precise wording must be followed.

If the respondent has difficulty choosing a response, or asks for clarification, the interviewer should repeat the question word for word and ask the respondent to answer in a way that most closely resembles his or her thoughts about his or her health today.

INTRODUCTION

(Note to interviewer: please read the following to the respondent.)

We are trying to find out what you think about your health. I will explain what to do as I go along, but please interrupt me if you do not understand something or if things are not clear to you. There are no right or wrong answers. We are interested only in your personal view.

First, I am going to read out some questions. Each question has a choice of five answers. Please tell me which answer best describes your health TODAY.

Do not choose more than one answer in each group of questions.

(Note to interviewer: first read all five options for each question. Then ask the respondent to choose which one applies to him/herself. Repeat the question and options if necessary. Mark the appropriate box under each heading. You may need to remind the respondent regularly that the timeframe is TODAY.)

For peer review only

EQ-5D DESCRIPTIVE SYSTEM

MOBILITY

First, I would like to ask you about mobility. Would you say that:

1. You have no problems in walking about? ❑
2. You have slight problems in walking about? ❑
3. You have moderate problems in walking about? ❑
4. You have severe problems in walking about? ❑
5. You are unable to walk about? ❑

SELF-CARE

Next, I would like to ask you about self-care. Would you say that:

1. You have no problems washing or dressing yourself? ❑
2. You have slight problems washing or dressing yourself? ❑
3. You have moderate problems washing or dressing yourself? ❑
4. You have severe problems washing or dressing yourself? ❑
5. You are unable to wash or dress yourself? ❑

USUAL ACTIVITIES

Next, I would like to ask you about usual activities, for example work, study, housework, family or leisure activities. Would you say that:

1. You have no problems doing your usual activities? ❑
2. You have slight problems doing your usual activities? ❑
3. You have moderate problems doing your usual activities? ❑
4. You have severe problems doing your usual activities? ❑
5. You are unable to do your usual activities? ❑

PAIN / DISCOMFORT

Next, I would like to ask you about pain or discomfort. Would you say that:

1. You have no pain or discomfort? ❑
2. You have slight pain or discomfort? ❑
3. You have moderate pain or discomfort? ❑
4. You have severe pain or discomfort? ❑
5. You have extreme pain or discomfort? ❑

ANXIETY / DEPRESSION

Finally, I would like to ask you about anxiety or depression. Would you say that:

1. You are not anxious or depressed? ❑
2. You are slightly anxious or depressed? ❑
3. You are moderately anxious or depressed? ❑
4. You are severely anxious or depressed? ❑
5. You are extremely anxious or depressed? ❑

For peer review only - http://bmjopen.bmj.com/site/about/guidelines.xhtml

EQ-5D VAS

- **Now, I would like to ask you to say how good or bad your health is TODAY.**

- **I would like you to try to picture in your mind a scale that looks like a thermometer.**
  (Note to interviewer: if interviewing face-to-face, please show the person the VAS scale.)

- **The best health you can imagine is marked 100 (one hundred) at the top of the scale and the worst health you can imagine is marked 0 (zero) at the bottom.**

- **I would now like you to tell me the point on this scale where you would put your health TODAY.**
  (Note to interviewer: mark the scale at the point indicating the respondent's 'health today'. Now, please write the number you marked on the scale in the box below.)

THE RESPONDENT'S HEALTH TODAY = ☐

Thank you for taking the time to answer these questions.

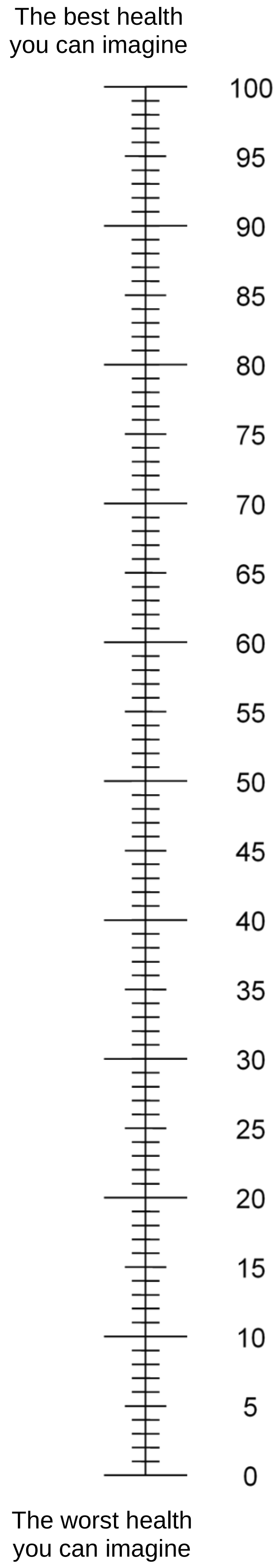

For peer review only - http://bmjopen.bmj.com/site/about/guidelines.xhtml

CONSORT 2010 checklist of information to include when reporting a randomised trial*

Section/Topic	Item No	Checklist item	Reported on page No
**Title and abstract**			
	1a	Identification as a randomised trial in the title	Page 1
	1b	Structured summary of trial design, methods, results, and conclusions (for specific guidance see CONSORT for abstracts)	Page 1
**Introduction**			
Background and objectives	2a	Scientific background and explanation of rationale	Page 2
	2b	Specific objectives or hypotheses	Page 3
**Methods**			
Trial design	3a	Description of trial design (such as parallel, factorial) including allocation ratio	Page 3
	3b	Important changes to methods after trial commencement (such as eligibility criteria), with reasons	Page 3
Participants	4a	Eligibility criteria for participants	Page 4
	4b	Settings and locations where the data were collected	Page 4 & 5
Interventions	5	The interventions for each group with sufficient details to allow replication, including how and when they were actually administered	Page 5 & 6
Outcomes	6a	Completely defined pre-specified primary and secondary outcome measures, including how and when they were assessed	Page 6 & 7
	6b	Any changes to trial outcomes after the trial commenced, with reasons	
Sample size	7a	How sample size was determined	Page 7 & 8
	7b	When applicable, explanation of any interim analyses and stopping guidelines	
Randomisation:			
Sequence generation	8a	Method used to generate the random allocation sequence	
	8b	Type of randomisation; details of any restriction (such as blocking and block size)	Page 5
Allocation concealment mechanism	9	Mechanism used to implement the random allocation sequence (such as sequentially numbered containers), describing any steps taken to conceal the sequence until interventions were assigned	Page 5
Implementation	10	Who generated the random allocation sequence, who enrolled participants, and who assigned participants to interventions	Page 5
Blinding	11a	If done, who was blinded after assignment to interventions (for example, participants, care providers, those	Page 5

For peer review only - http://bmjopen.bmj.com/site/about/guidelines.xhtml

		assessing outcomes) and how	
	11b	If relevant, description of the similarity of interventions	
Statistical methods	12a	Statistical methods used to compare groups for primary and secondary outcomes	Page 7
	12b	Methods for additional analyses, such as subgroup analyses and adjusted analyses	Page 7
**Results**			
Participant flow (a diagram is strongly recommended)	13a	For each group, the numbers of participants who were randomly assigned, received intended treatment, and were analysed for the primary outcome	See figure 1
	13b	For each group, losses and exclusions after randomisation, together with reasons	
Recruitment	14a	Dates defining the periods of recruitment and follow-up	
	14b	Why the trial ended or was stopped	
Baseline data	15	A table showing baseline demographic and clinical characteristics for each group	See figure 1
Numbers analysed	16	For each group, number of participants (denominator) included in each analysis and whether the analysis was by original assigned groups	
Outcomes and estimation	17a	For each primary and secondary outcome, results for each group, and the estimated effect size and its precision (such as 95% confidence interval)	See table 1 in page 12
	17b	For binary outcomes, presentation of both absolute and relative effect sizes is recommended	
Ancillary analyses	18	Results of any other analyses performed, including subgroup analyses and adjusted analyses, distinguishing pre-specified from exploratory	
Harms	19	All important harms or unintended effects in each group (for specific guidance see CONSORT for harms)	
**Discussion**			
Limitations	20	Trial limitations, addressing sources of potential bias, imprecision, and, if relevant, multiplicity of analyses	Page 2
Generalisability	21	Generalisability (external validity, applicability) of the trial findings	Page 2
Interpretation	22	Interpretation consistent with results, balancing benefits and harms, and considering other relevant evidence	
**Other information**			
Registration	23	Registration number and name of trial registry	Page 1
Protocol	24	Where the full trial protocol can be accessed, if available	NA
Funding	25	Sources of funding and other support (such as supply of drugs), role of funders	Page 10

*We strongly recommend reading this statement in conjunction with the CONSORT 2010 Explanation and Elaboration for important clarifications on all the items. If relevant, we also recommend reading CONSORT extensions for cluster randomised trials, non-inferiority and equivalence trials, non-pharmacological treatments, herbal interventions, and pragmatic trials. Additional extensions are forthcoming: for those and for up to date references relevant to this checklist, see www.consort-statement.org.

For peer review only - http://bmjopen.bmj.com/site/about/guidelines.xhtml

BMJ Open

A cluster randomised controlled trial to assess a tailored intervention to reduce antibiotic prescribing in rural China – Study protocol

Journal:	*BMJ Open*
Manuscript ID	bmjopen-2020-048267.R2
Article Type:	Protocol
Date Submitted by the Author:	16-Nov-2021
Complete List of Authors:	Cong, Wenjuan; University of Bristol, Population Health Sciences<br>Chai, Jing; Anhui Medical University, School of Health Service Management<br>Zhao, Linhai; Anhui Medical University, School of Health Services Management<br>Cabral, Christie; University of Bristol, Population Health Sciences<br>Yardley, Lucy; University of Bristol, School of Psychological Sciences<br>Yao, Guiqing; University of Leicester Department of Health Sciences<br>Zhang, Tingting; University of Bristol, Population Health Sciences<br>Cheng, Jing; Anhui Medical University, School of Health Services Management<br>Shen, XingRong; Anhui Medical University, School of Health Service Management<br>Liu, Rong; Anhui Medical University, School of Health Services Management<br>Little, Paul; University of Southampton, Primary Care and Population Science<br>Stuart, Beth; University of Southampton, Primary Care and Population Science<br>Hu, Xiaowen; Anhui Medical University, School of Health Service Management<br>Sun, Ye-Huan; Anhui Medical University, Department of Epidemiology and Biostatistics<br>Oliver, Isabel; Public Health England South Region, National Infection Service<br>Zheng, Bo; Peking University First Hospital, Institute of Clinical Pharmacology<br>Lambert, Helen; University of Bristol, Population Health Sciences<br>Wang, DeBin; Anhui Medical University, School of Health Services Management
**Primary Subject Heading**:	Public health
Secondary Subject Heading:	General practice / Family practice, Public health
Keywords:	PRIMARY CARE, Respiratory infections < THORACIC MEDICINE, GENERAL MEDICINE (see Internal Medicine)

A cluster randomised controlled trial to assess a tailored intervention to reduce antibiotic prescribing in rural China – Study protocol

Wenjuan Cong[1#], Jing Chai[2#], Linhai Zhao[2], Christie Cabral[1*], Lucy Yardley[3], Guiqing Yao[4], Tingting Zhang[1], Jing Cheng[2], XingRong Shen[2], Rong Liu[2], Paul Little[5], Beth Stuart[5], Xiaowen Hu[2], Ye-Huan Sun[6], Isabel Oliver[7], Bo Zheng[8], Helen Lambert[1], Debin Wang[2*]

[1] Population Health Sciences, University of Bristol, Bristol, UK
[2] School of Health Service Management, Anhui Medical University, Hefei, China
[3] School of Psychological Sciences, University of Bristol, Bristol, UK
[4] Department of Health Sciences, University of Leicester, Leicester, UK
[5] Primary Care and Population Science, University of Southampton, Southampton, UK
[6] Department of Epidemiology and Biostatistics, Anhui Medical University, Hefei, China
[7] Public Health England South Region, National Infection Service, Bristol, UK
[8] Institute of Clinical Pharmacology, Peking University First Hospital, Beijing, China
Corresponding Author: Prof Debin Wang; Contact Email: dbwang@vip.sina.com

For peer review only

UOB Confidential & Sensitive

Abstract

Introduction: Up to 80% of patients with respiratory-tract infections (RTI) attending healthcare facilities in rural areas of China are prescribed antibiotics, many of which are unnecessary. Since 2009, China has implemented several policies to try to reduce inappropriate antibiotic use; however antibiotic prescribing remains high in rural health facilities.

Methods and analysis: A cluster-randomised controlled trial will be carried out to estimate the effectiveness and cost effectiveness of a complex intervention in reducing antibiotic prescribing at township health centres in Anhui Province, China. 40 Township health centres will be randomised at a 1:1 ratio to the intervention or usual care arms. In the intervention group, practitioners will receive an intervention comprising: i) training to support appropriate antibiotic prescribing for RTI, ii) a computer-based treatment decision support system, iii) virtual peer support, iv) a leaflet for patients and v) a letter of commitment to optimise antibiotic use to display in their clinic.

The primary outcome is the percentage of antibiotics (intravenous and oral) prescribed for RTI patients. Secondary outcomes include patient symptom severity and duration, recovery status, satisfaction, antibiotic consumption. A full economic evaluation will be conducted within the trial period. Costs and savings for both clinics and patients will be considered and quality of life will be measured by EuroQoL (EQ-5D-5L).

A qualitative process evaluation will explore practitioner and patient views and experiences of trial processes, intervention fidelity and acceptability, and barriers and facilitators to implementation.

Ethics and dissemination: Ethical approval was obtained from the Biomedical Research Ethics Committee of Anhui Medical University (Ref: 20180259); the study has undergone due diligence checks and is registered at the University of Bristol (Ref: 2020-3137). Research findings will be disseminated to stakeholders through conferences and peer-reviewed journals in China, the UK and internationally.

Trial Registration: This trial has been registered at ISRCTN (ISRCTN30652037).

[#]WC and JC contributed equally

[*]DW and CC contributed equally

Corresponding Author: Prof Debin Wang; Contact Email: dbwang@vip.sina.com

For peer review only

UOB Confidential & Sensitive

Strengths and limitations of this study

- This is a large cluster-randomised trial to determine whether the intervention is effective in reducing antibiotic prescribing for patients with RTI in township health centres compared with usual care.
- This study will also evaluate the cost-effectiveness of an intervention to reduce antibiotic prescribing in rural areas.
- The quantitative and qualitative research in the trial could also provide recommendations on how to reduce antibiotics prescribing at village clinic level.
- The use of direct observation will ensure accurate assessment of antibiotic prescribing in both baseline and intervention phases.
- The study, though selected to be representative of rural populations in China, includes 10 counties from 4 regions across the Anhui Province, which may have limited generalisability.

Introduction

Antimicrobial resistance (AMR) specifically refers to the evolution of drug-resistant micro-organisms, including bacteria, which is driven by high rates of antibiotic use and is a pressing problem for global health and development.[1-3] If resistance continues to increase, common bacterial infections will eventually become untreatable.[4-5]

Since 2009, China has implemented the national essential medicines scheme and zero-mark-up policies to try to reduce inappropriate antibiotic use. [6-7] In 2012, the Chinese Ministry of Health issued a regulation to limit antibiotic prescribing to 20% of outpatient prescriptions in all patients.[8] However, the antibiotic prescribing rate in healthcare facilities in less developed or rural areas remains as high as 80%.[9-14]

In China patients can choose to go to the outpatient department of any health facility without a referral letter,[15] however, rural residents mainly seek health care from township health centres or village clinics, the two lowest-level health facilities in China's rural health care system. If patients develop severe symptoms, they are more likely to go directly to the outpatient clinic in the county hospital.[15-16] Many patients attend village clinics and township health centres with respiratory tract infections (RTIs) such as common cold, sore throat, and acute bronchitis and are frequently prescribed antibiotics even though most cases are likely to be viral and antibiotics are therefore unnecessary [17-20] Our previous study found that the antibiotic prescribing rate for RTI patients was up to 85% in rural Anhui Province.[21] In addition to the implications for AMR, there is a cost to patients [22-23] for extra health resource utilization (consultation fees, medication costs, loss of productivity costs), most of which would not be reimbursed through national medical insurance schemes. Therefore, reducing antibiotic prescribing in village clinics and township health centres is likely to be both safe and economically beneficial to patients. Since the start of the COVID-19 pandemic, village clinics have periodically been prohibited from providing treatment to RTI patients; therefore this trial focuses on provision of care in township health centres.

Township health centres in China are staffed with both qualified doctors and some health practitioners who are usually referred to as "village practitioners" but would not have a degree in medicine. Village practitioners are required to undergo basic training in health care provided by the local health care authority issued with a medical practice licence. Antibiotic prescribing in township health centres is

For peer review only

UOB Confidential & Sensitive

influenced by uncertainty about which RTI need antibiotics and by widespread beliefs that common infections should be treated with antibiotics.[15] Practitioners are also concerned about losing patients and believe that prescribing antibiotics helps maintain good relationships with their patients. Public campaigns on antimicrobial stewardship (AMS), [23-25] are very limited in rural areas and there is little technical support for village and township practitioners from senior clinical consultants and healthcare authorities, which makes it difficult for them to change norms of high antibiotic use.

This study will aim to determine whether the intervention safely reduces antibiotic prescribing for patients with RTI in township health centres. The study will provide evidence of effectiveness, value for money and barriers and facilitators to implementation that can inform policy and clinical practice. If pandemic conditions permit, the study will also pilot implementation of the same intervention in a limited number of village clinics to demonstrate proof of concept in lower level health facilities.

Methods and Analysis

Aims and objectives

This study aims to evaluate the effectiveness of a complex intervention aimed at healthcare practitioners and patients at township health centres, to reduce antibiotic prescribing for respiratory-tract infections (RTIs) compared with usual care in township health centres.

The primary outcome will be the percentage of antibiotics (intravenous and oral) prescribed for RTI patients.

Secondary objectives are to:

- estimate the cost-effectiveness of the intervention and the cost implications for both township health centres and patients.
- evaluate the impact of reduced antibiotic use on patient satisfaction with care.
- evaluate the impact of reduced antibiotic use on patient symptom severity, duration and recovery rates.
- investigate patient antibiotic consumption rates (prescribed or obtained from another source).
- understand practitioner and patient views of the intervention and barriers and facilitators to implementation.

Trial design

This is a pragmatic, two-armed (intervention *vs*. usual care) trial with an embedded cost-effectiveness and qualitative evaluations, with randomisation at township health centre level, using observed antibiotic prescribing to assess effectiveness.

Participants and recruitment procedures

Selection of township health centres

We originally started the trial recruitment at village clinic level. However, in May 2021 there was a small local COVID outbreak in Anhui Province, since when village clinics have not been allowed to treat RTI patients in Anhui and our trial recruitment had to be postponed. Currently patients with suspected Covid-19 symptoms (specifically patients with a temperature of more than 37.3 degree) must be referred to specialist COVID clinics or specialist COVID hospitals for treatment. We did some preliminary fieldwork in June 2021 and found it is still very difficult to recruit RTI patients at village clinic level and the research team assessed that this situation may not change within the next 12

For peer review only

UOB Confidential & Sensitive

months. However, we found that township health centres are still able to treat RTI patients regularly. In order to complete recruitment and maintain trial feasibility, we therefore decide to change the recruitment sites from village clinics to township health centres.

We will recruit 40 township health centres from 10 counties (4 township health centres per county), those counties will be randomly selected from 4 regions geographically dispersed across Anhui Province. We will randomly recruit 6 counties from 2 regions in densely populated North Anhui and 4 counties from another 2 regions from sparsely populated South Anhui. We will seek support from local county health authorities during the recruitment of township health centres. They will provide a list of townships in their area and the research team will randomly select 4 township health centres. Local county health authorities will screen the selected townships and may exclude selected townships, for which a reason will be recorded.

Recruitment of practitioners

The recruitment of health practitioners will be fair. Once a township health centre is recruited and has given permission for its staff to participate, all the practitioners working in the recruited township health centres will be invited to participate in the study. Once practitioners are identified and recruited, informed consent (Appendix 1, Supplementary material) will be obtained immediately.

Exclusion criteria
1, Practitioners who do not have basic computer skills
2, Practitioners who are not involved in clinical diagnosis and medicine prescribing

The background information of participating township health centres and practitioners will be collected. A senior practitioner in the recruited township health centre will complete a clinic background information questionnaire (Appendix 2, Supplementary material); and all practitioners working in the clinic will give their demographic data in a practitioner background information questionnaire (Appendix 3, Supplementary material).

A baseline audit of 400 RTI patients across the 40 township health centres will be conducted (10 RTI patients for each township health centre). This baseline audit will only collect primary outcome data, including the antibiotic prescribing rates at each clinic. Then all the township health centres will be randomised at a 1:1 ratio to usual care group vs intervention group, using the baseline antibiotic prescribing rates to balance high and low prescribing clinics between the arms. Each group will have 20 township health centres. (details see Fig. 1)

Recruitment of patients

All patients with RTIs who seek health care from the recruited health centres will be screened for eligibility and invited to participate in this study. Researchers from Anhui Medical University will undertake patient recruitment on-site until 30 participants have been recruited at each township health centre. Informed consent (Appendix 4, Supplementary material) will be obtained from all the patients who agree to participate in this study.

Currently, patients with suspected Covid-19 symptoms must be referred to specialist hospitals and township health centres are not expected to see suspected Covid-19 patients who present for treatment. However, some of these patients test negative for Covid-19 and are then referred back to their local clinic for treatment for their non-Covid RTI. These patients will be eligible for inclusion in our study.

For peer review only

UOB Confidential & Sensitive

Eligibility Criteria

Inclusion criteria

1. Adult (aged ≥18 years old)
2. Patient who is diagnosed with a RTIs in the recruitment consultation: blocked/runny nose, coughing with or without sputum, dry/sore throat, breathing problems, fever, ear inflammation (blocked ear, tinnitus, ear discharges, earache).

Exclusion criteria

1. Patients who have previously sought treatment for this illness from a normal clinic (not a specialist Covid clinic).
2. Patients who are unable to provide fully informed consent (eg dementia, psychosis, or severe depression)
3. Patients who are pregnant

Patients without bacterial infections, immunocompromised patients and patients with non-infective conditions are not excluded from this study if they present with any of the symptoms listed in the inclusion criteria. Patients who previously sought treatment for the same episode of illness in other health services (outpatient clinic in the county hospitals etc) will be excluded, simply because the treatment sought elsewhere may bring interference or contamination to our study findings by having previously obtained antibiotics elsewhere.

Randomisation, allocation & blinding

All the recruited township health centres will be stratified by above or below the average baseline antibiotic prescribing rate and randomised, in blocks of 4 and 6, at a 1:1 ratio to the intervention or usual care group. Antibiotic prescribing rate (%) of each township health centre will be measured by dividing the total number of RTI patients who are prescribed antibiotics (IV and oral) against the total number of RTI patients who are present to each township health centre.

All participating health centres will be allocated to a trial arm at the same time, after completion of baseline data collection. Due to the nature of the intervention, it is not possible to blind practitioners to their allocation to either intervention or usual care group. However, the statisticians conducting the analysis will remain blind to allocation until quantitative analysis is complete. The trial randomisation and blinding will be conducted by an independent statistician.

Intervention

The complex intervention comprises training, peer support, a computerised decision aid, a public commitment letter and a patient leaflet. These were co-designed with practitioners and patients using the person-based approach method for intervention development. [26]

We optimised the intervention tools using qualitative research to identify barriers to acceptability, feasibility and engagement that need to be addressed. We conducted four rounds of 'think-aloud' interviews with 37 practitioners (15 from township health centres and 22 from village clinics) and 17 Patients in rural Anhui. Think-aloud interviews obtained detailed user views by asking practitioners and patients to view contents of our intervention tools (training materials for practitioners, peer support groups, computer-based treatment decision support system, patient leaflet). Findings were immediately tabulated and analysed to identify changes required, modified materials used for the next round of think-aloud interviews and the process repeated until participant feedback indicated no further important, feasible and appropriate changes were needed. Through this process, we created

For peer review only

UOB Confidential & Sensitive

a feasible and generalisable intervention to reduce antibiotic prescribing tailored for the rural Chinese context. As an important element of the intervention, our computer-based treatment decision support system is adapted from a previous project where we set up a web-based just-in-time information system, and our colleagues had already tested the feasibility and acceptance of the system with a pilot study. [27] Additionally, we organised several trial steering group meetings where the intervention tools and its feasibility and generalisability were intensively discussed before we launched the trial.

Intervention practitioners will receive training delivered face to face in one session by a senior clinician from a tertiary hospital. The training covers: i) evidence about AMR in China and the link to their own practice; ii) addresses key concerns and misapprehensions; iii) details the current Chinese clinical guidelines on treatment of RTIs; iv) explains how to use the computer-based treatment decision support aid; and v) outlines communication strategies to improve patient acceptance of a non-antibiotic treatment decision.

Peer support groups will be established to connect the practitioners to others in the intervention group in order to provide peer support to each other during implementation. At the end of the training session intervention practitioners will be asked to organise themselves into peer support groups with a few practitioners in each and identify a lead for each peer support group. Peer support groups will meet via a widely used social media app in China (WeChat). The peer support group leader will schedule 2 structured Wechat meetings within a month after the training session. The group leader will be provided with a meeting guide to ensure a structured discussion and be responsible for setting up structured Wechat meetings of the peer support group, during which each member of the group will share their experience of applying the intervention (case discussion), discuss any problems and support each other to find solutions. Practitioners will be encouraged to contact each other outside of the scheduled meetings to ask for and provide support to each other. There will be another technical support group that will include the trial team and all intervention practitioners to provide technical assistance with intervention implementation. The senior clinician and two junior clinicians will be part of this group and be able to provide general guidance and reassurance that the intervention follows National Chinese prescribing guidance but will not provide specific clinical advice on cases.

A computer-based treatment decision support system will be installed on computers in consulting rooms in the intervention clinics. This will allow practitioners to record the patient's symptoms and the practitioner's diagnosis and will then provide the appropriate treatment guidance from the Chinese national guidelines. The treatment decision support system has been adapted from our previous project.[27] It contains the following sections (Appendix 5, Supplementary material): 1) patient eligibility criteria; 2) patient symptoms: allow practitioners to record what symptoms patients are suffering from; 3) diagnosis, patient reassurance & prognosis: allow practitioners to record their diagnosis, and provide appropriate patient reassurance and prognosis for each type of RTI patient; 4) treatment recommendations for each type of RTI patient from Chinese National Guidelines for Treating Acute Respiratory Tract Infections; 5) Table of symptomatic treatment options for RTI patients (also from Chinese National Guidelines); 6) Safety netting advice (when to come back to see the doctor) (also from the Chinese National Guidelines).

Practitioners will be required to sign a public declaration letter stating that they are committed to reducing unnecessary antibiotic prescribing and display this in a public area of their clinic.

Township health centres will be provided with a leaflet that can be given to RTI patients. This leaflet will address common patient beliefs and concerns about RTI, address common misapprehensions that can lead to an expectation of antibiotic treatment, provides advice on non-antibiotic symptomatic treatment options and safety-netting.

For peer review only

UOB Confidential & Sensitive

Data collection methods

Baseline data will be collected for 400 patients with RTI at the participating township health centres. Follow-up data collection will be collected for 1200 patients at the participating township health centres after implementation of the intervention.

In all recruited township health centre, direct observations will be carried out by a researcher from Anhui Medical University following the doctor questionnaire (Appendix 6, supplementary material) adapted from our previous study.[16] Observation will focus on the adult RTI clinical consultations and record patient symptoms, practitioner's diagnosis & treatment, sickness severity, any antibiotics or other medicines prescribed and mode of administration (IV or oral), name, dosage and quantity. Immediately after the consultation, the researcher will fill in a patient questionnaire (Appendix 7, supplementary material) by asking questions of patients directly. This questionnaire is also adapted from the previous study [16] and includes questions about patient demographic characteristics (gender, age, education level, residential area and medical insurance records), illness duration, symptom severity and patient satisfaction with the consultation; the latter two questions will be scored using a 10-point scale.

The researcher will conduct telephone interviews with recruited RTI patients at 7, 14, 21 days after their consultation. Structured questionnaires will be completed for each patient by the researcher during the telephone interview, with different secondary outcome data collected at different time points (see Table 1). Illness severity (scored on a 10-point scale) and symptom duration will be recorded at each time point. Patient attitude, understanding of the patient leaflet and beliefs will be recorded once at 7 days after consultation (Appendix 8, supplementary material). Patient continuing illness severity and recovery situation (when they feel better & when they feel they could get back to normal) will be checked at 7 days (Appendix 8, and 14 days after consultation (Appendix 9, Supplementary material). Patient consumption of antibiotics (including name, days, dosage and quantity) will be recorded at 7, 14 and 21 days after consultation (Appendix 10, Supplementary material). Through checking with patient's actual consumption of antibiotics (how many days they have taken the antibiotics, dosage etc) at different periods against the prescribed quantity of antibiotics (if applicable), patient adherence to follow practitioner's advice (%) could be estimated. Other healthcare use (clinic visits or consumption of other treatment) will be recorded at 21 days after consultation. Patient out-of-pocket spending due to this illness, including purchase of medication, fees for clinic attendance, days off-work , will be recorded at 21 days after consultation. Patient quality of life will be measured using the EQ5D-5L during the initial consultation, at 7, 14 & 21 days after consultation. (Use of paper interview administration mode of EQ5D-5L in Chinese version has been authorized by EuroQol Group (registration ID: 32880).

Data analysis

The primary analysis will use a generalised linear mixed model with a logit link and a random effect for clinic, allowing for the clustering of participants within clinics. The model will control for the clinic-level baseline antibiotic prescribing rate as well as potential confounders, and the full list of model covariates will be prespecified and set out in the statistical analysis plan. Results will be reported as odds ratios with 95% confidence intervals.

Missing data will be treated as missing at random and imputed using a chained-equations multiple imputation model. A complete cases analysis will be presented as a secondary analysis.

For peer review only - http://bmjopen.bmj.com/site/about/guidelines.xhtml

UOB Confidential & Sensitive

The analysis of secondary outcomes will use a similar modelling framework with distributions appropriate to the outcome, e.g. linear modelling for continuous outcomes and Poisson/negative binomial for count outcomes. These models will control for clustering and the factors listed above.

All results will be reported in line with the Cluster Randomised Trials extension to the CONSORT guidelines. No interim analyses are planned. Full details of the analyses to be undertaken will be set out in the Statistical Analysis Plan and approved by the Trial Steering Committee (TSC).

Sample size calculation

Our previous study recorded an antibiotic prescribing rate of 75% for rural township health centres in Anhui province [21, 28] and a previous pilot intervention showed a reduction in antibiotic prescribing of more than 20%. [27] There is no internationally agreed minimally important difference in antibiotic prescribing, however we anticipate that a reduction of at least 15% in antibiotic prescribing would represent a meaningful change at the population level given the very high rates of antibiotic prescribing.

To detect a possible absolute difference of 15% in prescribing rates between intervention (60%) and control practices (75%) with 90% power and alpha 0.05, we will need 203 patient consultations for infection RTI in each arm. Based on our previous study results the estimated ICC value was 0.05 and a design effect value would be 2.45. We would therefore aim to recruit at least 2.45*203*2=995 patients into the study. Allowing for a loss to follow-up rate of 10% and a cluster size of 30 patients per township health centre, we will recruit 40 township health centres into the study and each arm will have 20 township health centres.

Economic evaluation

The economic evaluation will be taken from a societal perspective and consider cost implications for patients as well as health services. Resource usage information will be costed using corresponding Chinese tariff.

All itemised resource usage data will be costed using appropriate data, with unit costs of visiting health care facilities based on medical service price items. Medication based on clinical consultation patient questionnaire and phone interviews with the patients at 7, 14 and 21 days; and the time spent costed at average wage in Anhui Province. Insurance coverage will be weighted to calculate private spending.

Resource use will be weighted by its unit cost over the study period. Accumulated costs per patient will be calculated for the study period. Quality of life data will be translated into a utility score using a Chinese tariff. Quality adjusted life years will be estimated using the area under the curve approach.

Missing data will be handled by multiple imputation methods. Difference in costs and QALYs will be compared using a generalised linear mixed model taking clinic as cluster level and by adjusting baseline clinical and quality of life variables. Bootstrapping methods will be used to estimate the incremental cost per QALYs gained and per percentage reduction of antibiotic prescribing. Major assumptions will be explored through sensitivity analyses. Completed cases analyses will be presented as one of such scenario analyses.

Qualitative Process Evaluation

A Mandarin-speaking qualitative researcher will conduct observations of the training process and intervention implementation. The key content related to participants during the training process, including any additions by the trainer, questions and answers, comments or discussion among

For peer review only - http://bmjopen.bmj.com/site/about/guidelines.xhtml

UOB Confidential & Sensitive

participants and non-verbal reactions displayed by participants will be recorded. The researcher will visit intervention clinics and observe how the intervention is being implemented.

A Mandarin-speaking qualitative researcher will conduct semi-structured interviews with the intervention practitioners. Short interviews will be conducted at the end of the training, to elicit intervention practitioners' views about the training. After intervention practitioners have had time to implement the intervention in their clinics, they will be invited to participate in a second semi-structured interview to understand their experiences of the intervention. They will be asked to describe how they used the intervention in their practice, any barriers and facilitators encountered in following the intervention and whether it influenced their views or practice.

A purposive sample of up to 40 patients who are recruited into the study will be interviewed. Patient will be selected to capture a range of socio-demographic characteristics and illness presentations. Some will be interviewed immediately after completing their consultation with health practitioners and some after they have completed the telephone questionnaires. Interviews will be semi-structured and guided by a topic guide that will cover experience of the consultation and understandings of the patient leaflet they received.

All semi-structured interviews will be transcribed. The qualitative data will be analysed thematically.

Data handling and repository

This trial will be monitored by a Trial Steering Committee and collected data will be monitored by data monitoring committee. There are no anticipated risks to patients from taking part in the study and participants have the right to withdraw from the study at any time without giving a reason.

Hard copies of documents with identifiable details of patient, practitioner and clinic will be stored in lockable cabinets on premises approved by the PI or relevant Co-Is at Anhui Medical University and any data created will be encrypted and stored on University secure servers until they can be uploaded or scanned and stored in the University of Bristol Research Data Storage Facility (RDSF) at the first available opportunity. Only authorised users can access data stored within the RDSF and procedures will be put in place to allow authenticated, external collaborators to view, add and/or edit data.

When possible, data will be exported directly to databases and consistency maintained via peer review and cross-checks with previous measurements. Manually inputted data will be double entered. Quality of questionnaire and interview data will be assured by training, provision of an interview manual and re-survey of 5% of subjects.

Patients and public involvement

Patients (and practitioners) contributed to the co-design of the intervention. They also provided feedback on piloted data collection forms, which led to modifications. The results will be disseminated to study participants and other stakeholders. At the end of the research project we will hold a high-level meeting to disseminate our findings to key stakeholders and develop a strategy for impact with relevant stakeholders.

ETHICS and dissemination

Ethical approval was obtained from the Biomedical Research Ethics Committee of Anhui Medical University (Ref: 20180259); the study has undergone due diligence checks and is registered at the University of Bristol (Case No: 2020-3137). This project is one of the three work packages in the UK-China AMR Centre Partnerships 2018 (Strategies to reduce the burden of antibiotic resistance in China)

For peer review only

UOB Confidential & Sensitive

supported by UKRI Medical Research Council (Grant Ref: MR/S013717/1)) and National Natural Science Foundation of China (Grant Ref: 81861138049). The protocol is rooted in a multidisciplinary collaboration between institutions in the UK and China.

Throughout the research project we will involve key stakeholders with roles in managing antibiotic use and antimicrobial stewardship. Research findings will be translated into short policy briefings with recommendations informed by existing policies in China and to influence current practice in health care settings. The credibility of research findings will be indicated to stakeholders through peer-reviewed publications in internationally recognised journals and presentations at academic conferences in China, the UK and internationally.

Author Contributions

HL, DW, CC, LY were responsible for developing the research questions and HL, DW, CC, LY, PL, BS, GY, IO, BZ were responsible for the study design. TZ, JC, JC, XS, RL, XH, YS are responsible for data collection and WC, JC & LZ are responsible for study management and coordination. WC, JC, BS, TZ, GY and CC drafted the paper. All authors read, commented on and approved the final manuscript.

Competing interests statement

None declared

Funding

The authors acknowledge MRC & Newton Fund through a UK-China AMR Partnership Hub award (MR/S013717/1), and National Natural Science Foundation of China award (81861138049).

References

1. Tenover FC. Mechanisms of Antimicrobial Resistance in Bacteria. *Am J Med*. 2006;119(6, Supp1):S3-S10.
2. Antimicrobial resistance: 2014 global report on surveillance. Geneva: World Health Organization; 2014.
3. Holmes AH, Moore LSP, Sundsfjord A, et al. Understanding the mechanisms and drivers of antimicrobial resistance. *Lancet*. 2016;387(10014):176-187.
4. Levy SB, Marshall B. Antibacterial resistance worldwide: causes, challenges, and responses. *Nat Med*. 2004;10(12):S122-S129.
5. Laxminarayan R, Matsoso P, Pant S, et al. Access to effective antimicrobials: a worldwide challenge. *Lancet*. 2016;387(10014):168-175.
6. Mao W, Chen W. CHINA: The Zero Mark-up Policy for essential medicines at primary level facilities. Health Systems, Governance & Financing, World Health Organization; 2015.
7. Xiao Y, Li L. China's national plan to combat antimicrobial resistance. *Lancet Infect Dis*. 2016;16(11):1216-1218.
8. Xiao Y, Li L. Legislation of clinical antibiotic use in China. *Lancet Infect Dis*. 2013;13(3):189-191.
9. Yang L, Liu C, Ferrier JA, et al. The impact of the National Essential Medicines Policy on prescribing behaviours in primary care facilities in Hubei province of China. *Health Policy Plan*. 2013;28(7):750-760.
10. Wang J, Wang P, Wang X, et al. Use and Prescription of Antibiotics in Primary Health Care Settings in China. *JAMA Intern Med*. 2014;174(12):1914-1920.
11. Wei X, Zhang Z, Walley JD, et al. Effect of a training and educational intervention for physicians and caregivers on antibiotic prescribing for upper respiratory tract infections in children at primary care facilities in rural China: a cluster-randomised controlled trial. *Lancet Glob Health*. 2017;5(12):e1258-e1267.
12. Guan X, Tian Y, Song J, et al. Effect of physicians' knowledge on antibiotics rational use in China's county hospitals. *Soc Sci Med*. 2019;224:149-155.

For peer review only

UOB Confidential & Sensitive

13. Zhan Q, Wang YL, Chen X. Evaluation of antibacterial use in outpatients of township and community primary medical institutions in a district of Sichuan Province, China. *J Glob Antimicrob Resist*. 2019;19:201-206.
14. Wang CN, Huttner BD, Magrini Net al. Pediatric Antibiotic Prescribing in China According to the 2019 World Health Organization Access, Watch, and Reserve (AWaRe) Antibiotic Categories. *J Pediatr*. 2020;220:125-131.e5.
15. Lambert H, Chen M, Cabral C. Antimicrobial resistance, inflammatory responses: a comparative analysis of pathogenicities, knowledge hybrids and the semantics of antibiotic use. *Palgrave Commun*. 2019;5(1):85.
16. Zhao L, Kwiatkowska RM, Chai J, et al. Pathways to optimising antibiotic use in rural China: identifying key determinants in community and clinical settings, a mixed methods study protocol. *BMJ Open*. 2019;9(8):e027819.
17. Gulliford MC, Dregan A, Moore MV, et al. Continued high rates of antibiotic prescribing to adults with respiratory tract infection: survey of 568 UK general practices. *BMJ Open*. 2014;4(10):e006245.
18. Zumla A, Al-Tawfiq JA, Enne VI, et al. Rapid point of care diagnostic tests for viral and bacterial respiratory tract infections—needs, advances, and future prospects. *Lancet Infect Dis*. 2014;14(11):1123-1135.
19. Godman B, Haque M, McKimm J, et al. Ongoing strategies to improve the management of upper respiratory tract infections and reduce inappropriate antibiotic use particularly among lower and middle-income countries: findings and implications for the future. *Curr Med Res Opin*. 2020;36(2):301-327.
20. Wang CN, Huttner BD, Magrini N, et al. Pediatric Antibiotic Prescribing in China According to the 2019 World Health Organization Access, Watch, and Reserve (AWaRe) Antibiotic Categories. *J Pediatr*. 2020;220:125-31.e5.
21. Chai J, Coope C, Cheng J, et al. Cross-sectional study of the use of antimicrobials following common infections by rural residents in Anhui, China. *BMJ Open* 2019; 9:e024856.
22. Song J-H, Thamlikitkul V, Hsueh P-R. Clinical and economic burden of community-acquired pneumonia amongst adults in the Asia-Pacific region. *Int J Antimicrob Agents*. 2011;38(2):108-117.
23. Wang X, Xuan Z, Storella TH, et al. Determinants of non-prescription antibiotic dispensing in Chinese community pharmacies from socio-ecological and health system perspectives. *Soc Sci Med*. 2020:113035.
24. Xiao Y, Shen P, Zheng B, et al. Change in Antibiotic Use in Secondary and Tertiary Hospitals Nationwide After a National Antimicrobial Stewardship Campaign Was Launched in China, 2011-2016: An Observational Study. *J Infect Dis*. 2020;221(2):S148-S155.
25. Xiao Y. Antimicrobial Stewardship in China: Systems, Actions and Future Strategies. *Clin Infect Dis*. 2018;67:S135-S141.
26. Yardley L, Morrison L, Bradbury K, et al. The person-based approach to intervention development: application to digital health-related behavior change interventions. *J Med Internet Res.* 2015;17(1):e30.
27. Shen X, Lu M, Feng R, et al. Web-Based Just-in-Time Information and Feedback on Antibiotic Use for Village Doctors in Rural Anhui, China: Randomized Controlled Trial. *J Med Internet Res*. 2018;20(2):e53.
28. Shen X, Shen J, Pan Y, et al. Clinical diagnosis and treatment of common respiratory tract infections in relation to microbiological profiles in rural health facilities in China: implications for antibiotic stewardship. *BMC Fam Pract* 2021; 22:87-95.

For peer review only

UOB Confidential & Sensitive

Table 1 List of the primary and secondary outcome measures and when they will be collected at follow-up audit.

Item	Outcome measure	Initial consultation	Patient Telephone interview after consultation		
			7 days	14 days	21 days
Primary outcome	Prescribing rate of antibiotics (%)	√			
Secondary outcomes	Patient satisfaction	√			
	Patient symptom severity and duration		√	√	√
	Patient recovery		√	√	
	Patient consumption of antibiotics		√	√	√
	Patient attitude, beliefs and understanding of the patient leaflet		√		
	Patient costs				√
	Patient quality of life (EQ5D-5L)	√	√	√	√

For peer review only

UOB Confidential & Sensitive

Figure legend

Fig 1 Trial flow chart

Supplementary material

Appendix 1 Health practitioner consent form
Appendix 2 Patient consent form
Appendix 3 Health practitioner consent form
Appendix 4 Practitioner background information questionnaire
Appendix 5 Computer-based treatment decision support system content
Appendix 6 Clinical consultation doctor questionnaire
Appendix 7 Clinical consultation patient questionnaire
Appendix 8 Telephone questionnaire – Day 7
Appendix 9 Telephone questionnaire – Day 14
Appendix 10 Telephone questionnaire – Day 21

For peer review only

UOB Confidential & Sensitive

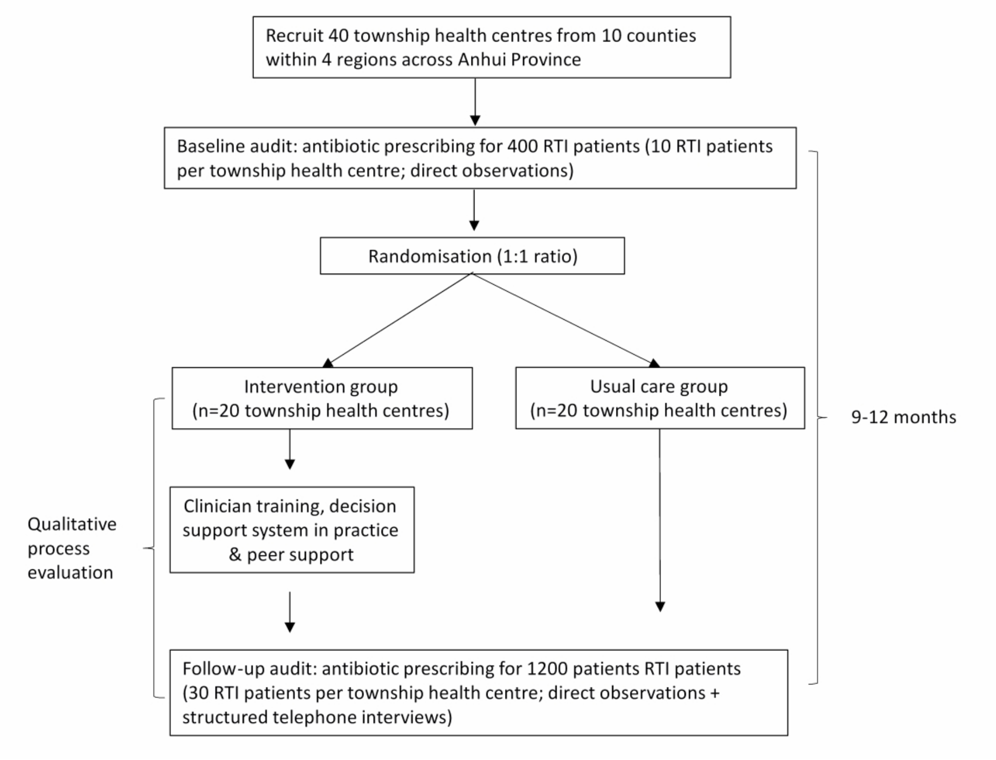

Fig 1 Trial flow chart

196x149mm (600 x 600 DPI)

Study Number:

Participated Clinic Identification Number for this trial:

CONSENT FORM

Title of Project: Design and evaluate a tailored intervention to modify antibiotic prescribing behaviour among health professionals and reduce antibiotic consumption: a cluster randomised controlled trial

Name of Researcher:

Please initial all boxes

1. I confirm that I have read and understand the information sheet dated [**DATE**] (version 1) for the above study. I have had the opportunity to consider the information, ask questions and have had these answered satisfactorily. ☐
2. I understand that my participation is voluntary and that I am free to withdraw at any time without giving any reason, without my medical care or legal rights being affected. ☐
3. I understand that any information I provide will be anonymised by removing all identifying details and will be kept strictly confidential and used only for research purposes. After the study, the anonymised information will be made publicly available for potential further research, but it will not be possible to identify me from the data. ☐
4. I understand that with my agreement, the interview may be audio-recorded but no one except the research team will hear this recording. I am aware that anonymised quotes from the interview may be included in reports of the study findings. ☐
5. I agree to take part in the above study. ☐

________________	________________	________________
**Name of Participant**	**Date**	**Signature**

________________	________________	________________
**Name of Person taking consent (if applicable)**	**Date**	**Signature**

________________	________________	________________
**Name of Researcher**	**Date**	**Signature**

Consent form date of issue: **[DATE]**
Consent form version number: **[VERSION]**

For peer review only - http://bmjopen.bmj.com/site/about/guidelines.xhtml

Township health centre (THC) background information questionnaire

乡镇卫生院基本情况调查表

This questionnaire should be filled by the head of the THC
这张问卷请由此乡镇卫生院的负责医生填写。

名称：_______（市）_______县（区）________乡（镇）__________村
_______township (town),_______county, _______city

一、乡镇卫生院的基本情况

Basic information of THC

1、按照设置规划，本乡镇卫生院服务<br>1．1 覆盖地域面积为_______平方公里；<br>1．2 服务覆盖总人口_________人；<br>1．3 其中，常住人口________人；	1.The information about services of the township health centre<br>1.1 What is the service coverage area? _________square kilometers;<br>1.2 What is the total population covered by the service? ______;<br>1.3 What is the number of permanent residents covered by the service? ______;
2、目前，本乡镇卫生院所服务的行政村，实际共有其他村级医疗机构______个，并请说明医疗机构名称_______	2. At present, the administrative townships served by the THC actually have ______other township level medical institutions, *[please specify their names]* __________
3、是否提供中医服务？（1）是 （2）否<br>3.1 包括哪些中医服务？<br>□草药；□针灸；□推拿；□其他______	3. Is TCM service provided here?<br>(1) Yes; (2) No<br>3.1 What TCM services are included?<br>□ herbal; □acupuncture; □ massage; □ other *[please specify]* _________
4、本乡镇卫生院能提供的医学检查有哪些？<br>□ 血液检查<br>□ X 射线<br>□ CT<br>□ B 超<br>□ 心电图检查<br>□ 没有<br>□ 其他检查手段，请描述______	4. What kinds of RTI related tests can this THC provide?<br>□Blood test<br>□X-Ray<br>□CT<br>□B Ultrasound<br>□ECG<br>□None<br>Other (Please Specify) _______
5、本乡镇卫生院有医生数量_________个.	5. The THC has_____ outpatient clinics; these clinics have _____doctors in total.

For peer review only

二、乡镇卫生院的医疗服务情况

Medical services in village clinics

2.过去一年接诊多少门诊病人？__________<br>2．1 其中，呼吸道感染的病人多少人？___	2. How many outpatients are there in the past year?<br>2.1 How many patients with respiratory infection?

For peer review only

Health practitioner background information questionnaire

乡镇卫生院医生基本情况调查表

This questionnaire should be filled by all clinicians working in this township health centre.

全部在此乡镇卫生院工作的医生都需要填写此表。

Gender?<br>□Male<br>□Female	性别?<br>□男<br>□女
Date of birth? ___________<br>Age: _____years.	出生日期：_____年_____月_____日；<br>或年龄？ ______岁
Education?<br>□ middle school and below;<br>□ high school /polytechnic school / technical school;<br>□ junior college;<br>□ undergraduate;<br>□ postgraduate and above;<br>□ others, please specify_____	学历？<br>□初中及以下；<br>□高中/中专/技校；<br>□专科；<br>□大学本科；<br>□研究生及以上；<br>□其他，请说明_____
How many years of medical training have you received? _______years；	你受到多少年的医学专业训练？<br>_______年；
What kind of medical training/learning do you have? What is your specialty?<br>□ Clinical medicine;<br>□ TCM;<br>□ Nursing;<br>□ Others, please specify_______	所学专业？<br>□临床医学；<br>□中医学；<br>□护理学；<br>□其他，请说明_______
How long have you worked as a physician? _______years；	您从事医生的工作年限？ _______年；
How long have you worked here? _______years；	你在这里工作多久了？ _______年；
Type of certificate you obtained:<br>□ licensed physician;<br>□ Certified assistant physician;<br>□ Registered nurse;<br>□ Village doctor;<br>□ No certificate	你获得证书类别：<br>□执业医师；<br>□执业助理医师；<br>□注册护士；<br>□乡村医生；<br>□无证书
Category of doctor practice:<br>□ Clinical;<br>□ Chinese medicine;<br>□ Public health<br>□ Others, please specify_______	医师执业类别：<br>□临床；<br>□中医；<br>□公共卫生<br>□其他，请说明_______
Do you have any further study experience? How many months in total?；<br>□ Yes (if yes, total months? _______months) ;<br>□ No;	你有没有进修的经历？<br>□有（如果有，共_____个月？）；<br>□无；
Have you ever participated in the training on the use of antibiotics? □Yes;□No;<br>If yes, how many times? _______times;<br>How many months in total? _______months.	有没有参加过关于抗菌药物使用的培训？<br>如果有，几次？<br>累计多少个月？

For peer review only

Study Number:

Patient Identification Number for this trial:

CONSENT FORM

Title of Study: Appropriate use of antibiotics for RTI in primary care: a cluster randomised controlled trial

Name of Researcher: XXX (contact details)

	**Please initial all boxes**
I confirm that I have read information sheet /went through information sheet with the researcher. I understand it and has had the opportunity to ask questions.	
I understand that my participation is voluntary and that I am free to withdraw at any time without giving any reason, without my medical care being affected.	
I understand that all the information is confidential. No name or personal information will be used when the results of this study is published.	
I agree to take part in the above study.	

**Name of Participant**	**Date**	**Signature**
**Name of Person taking consent (if applicable)**	**Date**	**Signature**
**Name of Researcher**	**Date**	**Signature**

**Participant contact details (used for telephone questionnaire)**	
Phone number	
WeChat ID	

Consent form date of issue: [DATE]
Consent form version number: [VERSION]

For peer review only - http://bmjopen.bmj.com/site/about/guidelines.xhtml

For peer review only

Decision Support System & in clinic data collection items – for HP to use within consultation 诊断支持系统

Page 1: Eligibility criteria

第一页：入选条件

**Does patient have a suspected RTI?**	**病人是否疑似呼吸系统疾病?**
☐ Yes<br>☐ No – exclude from study	☐ 是<br>☐ 否—从本研究中排除
**Is today the first time the patient has sought treatment for this illness or a repeat visit?**	**今天是病人为此病的首次还是再次就诊?**
☐ First time consultation<br>☐ Repeat consultation – exclude from study	☐ 首次就诊<br>☐ 再次就诊--从本研究中排除
**Does patient need to be transferred due to COVID-19 suspected symptoms?**	**病人是否因疑似新冠需要被转入发热门诊?**
☐ Yes – exclude from study<br>☐ No	☐ 是 – 从本研究中排除<br>☐ 否

Page 2: Patient Symptoms

第二页：病人症状

**Consultation – Respiratory illness – Initial consultation – Illness history**	
**What symptoms are patient suffering from?** (tick all that apply)	**病人具体都有哪些症状?** (勾选所有符合的症状)
☐ Nasal congestion and running nose<br>☐ Cough<br>☐ Expectoration<br>☐ Sore throat<br>☐ Congestion/swelling of tonsils<br>☐ Fever<br>☐ Headache<br>☐ Earache	☐ 鼻塞，流鼻涕<br>☐ 咳嗽<br>☐ 咳痰<br>☐ 嗓子疼<br>☐ 扁桃体肿大<br>☐ 发烧<br>☐ 头疼<br>☐ 耳朵疼

For peer review only

☐ Muscular stiffness<br>☐ Chest congestion or shortness of breath<br>☐ Others	☐ 肌肉僵硬<br>☐ 胸闷或呼吸急促<br>☐ 其他______________

Page 3 : Diagnosis, prognosis & patient reassurance

第三页：诊断，预后及病人安慰

Tick one	**What is the diagnosis?**	选一项打勾	**诊断是什么?**
	1. Acute upper respiratory tract infection including:<br>• Infectious cold / Acute rhinitis / upper respiratory tract catarrh<br>• Acute inflammation of the throat<br>• Acute viral pharyngitis<br>• Acute viral laryngitis		1.急性上呼吸道感染:<br>• 传染性感冒/急性鼻炎/上呼吸道粘膜炎<br>• 喉咙急性炎症<br>• 急性病毒性咽炎<br>• 急性病毒性喉炎
	2. Acute tonsil inflammation / Tonsillitis / Pharyngitis		2.急性化脓性扁桃体炎/ 扁桃体炎/咽炎
	3. Acute ear inflammation / Otitis Media		3.急性耳朵炎症/中耳炎
	4. Acute sinusitis		4.急性鼻窦炎
	5. Acute tracheal/ bronchial inflammation / Acute Tracheobronchitis / Bronchitis / Lower respiratory tract infection		5.急性气管/支气管炎症/急性气管支气管炎/支气管炎/下呼吸道感染
	6. Pneumonia		6.肺炎
	Other please state [free text box]		其他请说明

**Patient Reassurance**	**病人安慰**
• If you have diagnosed condition 1-5 reassure the patient that there is no sign of serious infection. Here are some things you can say to reassure patients:<br>○ There is no evidence of a serious infection<br>○ In your case the body can fight infections well on its own<br>○ You can take remedies to ease the symptoms<br>• If you have diagnosed Pneumonia, move straight on to Prognosis (next step) and Treatment (page 4).	• 如果您确诊病人所患疾病为 1-5 中之一，请确保该病人没有严重感染的迹象。以下是一些可以让患者放心的语句:<br>○ 没有严重感染的证据<br>○ 就你的情况而言，你的身体可以自己抵抗感染<br>○ 您可以使用一些疗法来缓解症状<br>• 如果您确诊病人所患疾病为肺炎，请直接进行预后（下一步）和治疗（第四页）

For peer review only

Prognosis (Note only one option will apply)			病人预后 （只选一项）		
Tick one	Most infections of this type are better by:		选一项打勾	此类感染一般情况下的好转时间是:	
	1. Acute upper respiratory tract infection	14 days		1.急性上呼吸道感染	14 天
	2. Acute tonsil inflammation / Tonsillitis / Pharyngitis	7-8 days		2. 急性化脓性扁桃体炎/ 扁桃体炎/咽炎	7-8 天
	3. Acute ear inflammation / Otitis Media	8 days		3. 急性耳朵炎症/中耳炎	8 天
	4. Acute sinusitis	14-21 days		4. 急性鼻窦炎	14-21 天
	5. Acute tracheal/ bronchial inflammation / Tracheobronchitis / Bronchitis / Lower respiratory tract infection	21 days		5. 急性气管/支气管炎症/急性气管支气管炎/支气管炎/下呼吸道感染	21 天
	6. Pneumonia	● 1 week: fever should have resolved<br>● 4 weeks: chest pain and sputum production should have substantially reduced<br>● 6 weeks: cough and breathlessness should have substantially reduced<br>● 3 months: most symptoms should have resolved but fatigue may still be present<br>● 6 months: most people will feel back to normal		6. 肺炎	• 1 周：发烧应该已经解决了<br>• 4 周：胸痛和痰的产生应该大幅减轻<br>• 6 周：咳嗽和呼吸困难应该大幅减轻<br>• 3 个月：大多数症状应该已经解决，但仍可能存在疲劳<br>• 6 个月：大多数人都会感觉恢复正常

For peer review only

Page 4: treatment recommendations (Note only one option will apply – select correct option for your patient)

第四页：治疗建议（只选一项， 请为您的病人选择正确的治疗建议）

**Treatment recommendation for each type of RTI (only one type of RTI below will appear based on the diagnosis choice in page 3)**	**每类呼吸道疾病的治疗建议（根据您第三页的诊断结果，以下六种疾病的治疗建议只有一种会出现）**
1. Acute upper respiratory tract infection<br>• Treatment should prioritize targeting symptoms and supporting the body to recover<br>• Antibiotics are usually not to be used and can be harmful, potentially causing side effects that can be severe, and increasing the risk of future serious antibiotic resistant infections in the patient, their family and the community<br>• See table below for symptomatic treatment options (there are just suggestions that come from Chinese National Guidelines)	1. 急性上呼吸道感染<br>• 治疗应优先针对症状并支持身体恢复<br>• 抗生素通常不被使用。其可能有害，并且可能导致严重的副作用并增加患者，其家人和社区未来严重抗生素耐药性感染的风险<br>• 有关症状治疗方案，请参见下表一（表中为国家指南的建议，供参考）
2. Acute tonsil inflammation / Tonsillitis / Pharyngitis<br>• Treatment should prioritize targeting symptoms and supporting the body to recover<br>• Antibiotics are usually not to be used and can be harmful, potentially causing side effects that can be severe, and increasing the risk of future serious antibiotic resistant infections in the patient, their family and the community<br>• See table 1 below for symptomatic treatment options<br>• Consider using antibiotics when:<br>○ There is suppuration of tonsils<br>○ Temperature of >38.5ºC	2. 急性扁桃体炎/扁桃体炎/咽炎<br>• 治疗应优先针对症状并支持身体恢复<br>• 抗生素通常不被使用。其可能有害，并且可能导致严重的副作用并增加患者、其家人和社区未来严重抗生素耐药性感染的风险<br>• 有关症状治疗方案，请参见表一<br>• 在下列情况下考虑使用抗生素<br>- 有扁桃体的化脓<br>- 体温> 38.5 ºC
3. Acute ear inflammation / Otitis Media<br>• Antibiotics are usually not to be used and can be harmful, potentially causing side effects that can be severe, and increasing the risk of future serious antibiotic resistant infections in the patient, their family and the community<br>• See table 1 below for symptomatic treatment options<br>• Consider using antibiotics if one or more of these symptoms are present (although note that the first three symptoms are common in viral ear infections):<br>○ loss of hearing<br>○ elevated temperature<br>○ progressive swelling and hyperemia in the tympanic membrane<br>○ perforation of the tympanic membrane accompanied by yellow exudate	3. 急性耳朵炎症/中耳炎<br>• 抗生素通常不被使用。其可能有害，并且可能导致严重的副作用并增加患者、其家人和社区未来严重抗生素耐药性感染的风险<br>• 有关症状治疗方案，请参见表一<br>• 如果存在以下一种或多种症状，请考虑使用抗生素（尽管研究已表明前三种症状在病毒性耳朵炎症中也是常见的）：<br>- 丧失听力<br>- 体温升高

For peer review only

	- 鼓膜中的进行性肿胀和充血<br>- 鼓膜穿孔伴有黄色渗出物
4. Acute sinusitis<br>• Antibiotics are usually not to be used and can be harmful, potentially causing side effects that can be severe, and increasing the risk of future serious antibiotic resistant infections in the patient, their family and the community<br>• See table 1 below for symptomatic treatment options<br>• Consider using antibiotics if one or more of these symptoms are present:<br>○ Symptoms for more than 10 days<br>○ Discoloured or purulent nasal discharge<br>○ Severe localised unilateral pain (particularly pain over teeth and jaw)<br>○ Fever<br>○ Marked deterioration after an initial milder phase	4. 急性鼻窦炎<br>• 抗生素通常不被使用。其可能有害，并且可能导致严重的副作用并增加患者、其家人和社区未来严重抗生素耐药性感染的风险<br>• 有关症状治疗方案，请参见表一<br>• 如果存在以下一种或多种症状，请考虑使用抗生素：<br>- 症状超过 10 天<br>- 褪色或化脓性鼻涕<br>- 严重的局部单侧疼痛（尤其是牙齿和下颌疼痛）<br>- 发烧<br>- 初始缓和阶段后出现明显恶化
5. Acute tracheal/ bronchial inflammation / Tracheobronchitis / Bronchitis / Lower respiratory tract infection<br>• Symptomatic treatment should be considered priority.<br>• Antibiotics should not be routinely used and can be harmful, potentially causing side effects that can be severe, and increasing the risk of future serious antibiotic resistant infections in the patient, their family and the community.<br>• Consider anti-microbial treatment in the following cases:<br>○ patients over 75 years of age with an elevated temperature;<br>○ patients with heart failure;<br>○ patients with insulin-dependent diabetes;<br>○ patients with severe diseases of the nervous system.	5. 急性气管/支气管炎症/气管支气管炎/支气管炎/下呼吸道感染<br>• 首先考虑对症治疗<br>• 抗生素不应常规使用。其可能有害，并且可能导致严重的副作用并增加患者、其家人和社区未来严重抗生素耐药性感染的风险<br>• 在以下情况下考虑抗生素治疗：<br>- 患者 75 岁以上并伴体温升高<br>- 心力衰竭患者<br>- 胰岛素依赖型糖尿病患者<br>- 患有严重神经系统疾病的患者
6. Pneumonia<br>• The decision for outpatient or inpatient treatment should be based on the severity of illness, and anti-infection therapy should be administered as early as possible.<br>• Patients with mild symptoms and normal GI function should receive treatment with oral medication<br>• Patients with heavy symptoms should receive IV treatment, and change to oral medication once there is clear improvement in clinical appearance and ability to receive oral medication.<br>○ Young adults, patients with no underlying conditions	6. 肺炎<br>• 门诊或住院治疗取决于疾病的严重程度，并应尽早进行抗感染治疗<br>• 症状轻微且胃肠功能正常的患者应接受口服药物治疗<br>• 症状较重的患者应接受静脉注射治疗，一旦临床表现和接受口服药物的能力明显改善应改为口服药物治疗<br>- 年轻人，没有潜在疾病的患者

For peer review only
For peer review only - http://bmjopen.bmj.com/site/about/guidelines.xhtml

        - Penicillin; Amoxicillin; Doxycycline, minocycline; 1st or 2nd gen cephalosporins; Respiratory quinolones (moxifloxacin, levofloxacin, and Gemifloxacin)
    - The elderly or patients with underlying conditions
        - Monotherapeutic usage of 2nd gen cephalosporins (cefuroxime axetil, cefprozil, cefaclor etc.) or combined usage with macrolides; Monotherapeutic usage of amoxicillin/clavulanic acid or ampicillin/sulbactam or combined usage with macrolides; Respiratory quinolones

Referral criteria (they are suggestions from Chinese National Guidelines; they may not all be useful or relevant to you):

- Combined with other underlying illnesses, such as chronic cardiac insufficiency (III-IV)
- Immunosuppression
- Initial treatment is failed but the vital signs are stable
- Local or systemic complications, such as pulmonary abscess, but vital signs are stable
- Older than 65 years of age with underlying illnesses, and assessed as having the infection risk of ESBL and other multi-resistant strains
- CAP identification is not clear and requires further diagnosis

        - 青霉素；阿莫西林；强力霉素，米诺环素；第一代或第二代头孢菌素；呼吸喹诺酮类药物（莫西沙星，左氧氟沙星和吉米沙星）
    - 老年人或有潜在疾病的患者
        - 第二代头孢菌素（头孢呋辛酯，头孢丙烯，头孢克洛等）的单药治疗或与大环内酯类联合使用；阿莫西林/克拉维酸或氨苄青霉素/舒巴坦的单药治疗用途或与大环内酯联合使用；呼吸喹诺酮类

普通转诊（下列是国家指南的建议；以下建议不一定全部有用或者符合您当时的情况）：

- 合并基础疾病较多，如慢性心功能不全 (Ⅲ。Ⅳ级)、慢性肾脏疾病 3 ~ 5 期、肝硬化失代偿、 糖尿病急症
- 免疫抑制宿主发生 CAP
- 初始治疗失败，生命体征稳定
- 出现局部或全身并发症，如脓胸、肺脓肿， 生命体征稳定
- 年龄 / >65 岁有基础疾病患者，评估有超广 谱β-内酰胺酶 (ESBL)菌等耐多药感染风险
- CAP 诊断尚未明确，需要进一步鉴别诊断

Table 1 Symptomatic treatment options for RTI

表一 呼吸道感染对症治疗措施

**Symptoms**	**Symptomatic treatment options**	**症状**	**对症治疗措施**
temperature<br>headache<br>all-over muscle pain<br>pain in any area	Antipyretic analgesics including:<br>• Paracetamol<br>• Aspirin<br>• Ibuprofen*	发烧<br>头痛<br>全身肌肉疼痛<br>身体任何部位疼痛	解热镇痛药，包括：<br>• 对乙酰氨基酚/扑热息痛<br>• 阿司匹林<br>• 布洛芬*
blocked nose<br>hyperemia<br>edema in the nasal mucosa<br>sore throat	Compound decongestant and anti-allergenic medicine including:<br>• Pseudoephedrine<br>• 1% ephedrine nasal spray	鼻塞<br>充血<br>鼻粘膜水肿	复方抗充血剂和抗过敏药物，包括：<br>• 伪麻黄碱<br>• 1%麻黄素喷雾

For peer review only

		嗓子疼	
frequent sneezing<br>continual runny nose	Compound decongestant and anti-allergenic medicine including:<br>• chlorphenamine maleate<br>• loratadine<br>• diphenhydramine	频繁打喷嚏<br>持续流鼻涕	复方抗充血剂和抗过敏药物，包括：<br>• 马来酸氯苯那敏<br>• 氯雷他定<br>• 苯海拉明
Cough	Cough suppressants including:<br>• dextromethorphan hydrobromide<br>• codeine	咳嗽	止咳药，包括<br>• 右美沙芬<br>• 可待因
'Inflammation'	TCM preparations may be effective<br>• yinqiao tablets<br>• shuanghuanglian<br>• antiviral granules<br>• yuxingcao (fish leaf)	'炎症'	以下中成药或中药成分可能对你有效，包括：<br>• 银翘片<br>• 双黄连<br>• 抗病毒颗粒<br>• 鱼腥草

*Current Chinese national guidelines recommend using ibuprofen for symptoms of temperature, headache, all-over muscle pain and pain in any area; however, there is some research to show that the use of ibuprofen is unlikely to help the symptoms and is associated with their progression. Recent study has suggested that advising the use of ibuprofen may be unhelpful in managing RTIs.

*目前国家指南建议将布洛芬应用于发烧，头痛等呼吸道感染症状的对症治疗中。然而一些国外研究已经发现布洛芬对于其症状缓解没有帮助甚至会加重病情，已有研究提出布洛芬在呼吸系统感染治疗中是没有帮助的。

**Advice for all patients with diagnosis 1-5**	**对于所患疾病为 1-5 的病人的建议：**
The best way is to support your own body's immune system to fight off the infection by, for example:<br>·Drink fluid (preferably water) at least 6 to 8 glasses of every day, particularly if you also have a fever;<br>·Keep warm and get plenty of rest;<br>·Eat a healthy, balanced diet containing plenty of fruit and vegetables.	最好的方法就是提高你自身的免疫力以预防感染，例如<br>• 每天至少喝 6 到 8 杯水（首选白开水），特别是发热的时候<br>• 保暖，多休息<br>• 健康均衡饮食，多吃水果蔬菜

For peer review only

**Things you can say to patients to help them understand that they do not need antibiotics**	**你可以给与病人以下建议，来帮助他们明白他们并不需要抗生素**
• Scientific research has found that antibiotics (e.g. Amoxicillin and Cephalosporin) do not help coughs, colds, sore throat or earache get better any faster;	• 科学研究发现抗生素（例如阿莫西林和头孢）并不能帮助咳嗽，感冒，嗓子疼，耳朵疼更快地好转
• Research has shown that in the past we took lots of antibiotics, such as Amoxicillin and Cephalosporin that we did not need	• 研究表明，在过去我们服用了很多我们不需要的抗生素，例如阿莫西林和头孢
• Taking antibiotics (e.g. Amoxicillin and Cephalosporin) may weaken your own body's methods for keeping you healthy, by killing the good bacteria in your gut.	• 服用抗生素（例如阿莫西林和头孢）会杀死肠道中的有益细菌，从而削弱身体保持健康的能力
• Taking antibiotics (e.g. Amoxicillin and Cephalosporin) for common infections now means that in the future antibiotics will work less well or not at all for you and your family	• 现在对常见的感染就使用抗生素（例如阿莫西林和头孢），意味着在未来，抗生素对你和你的家人的作用会更差或者根本不起作用
• Antibiotics can cause side effects (such as diarrhoea, rashes, feeling sick), which in rare cases can be very severe.	• 抗生素可以引起副作用(如腹泻、皮疹、恶心等)，在极少数情况下可能会非常严重

For peer review only

Page 5: Safety netting advice (when to come back to see the doctor)

第五页：复诊建议（何时再来看医生）

Finally, in the event of the following, please come back for treatment right away:	最后，如果出现以下现象请立即复诊:
. If your skin is very cold or has a strange colour, or you develop an unusual rash	• 如果你皮肤温度很低或者出现奇怪的颜色，或者出现异样皮疹
. If you feel confused have slurred speech, or become drowsy	• 出现头脑不清、言语不清、瞌睡
. If you have difficulty breathing including:<br>. hyperventilating<br>. your lips become purple<br>. skin between or above the ribs getting sucked or pulled in with every breath	• 出现呼吸困难，包括:<br>- 呼吸急促<br>- 嘴唇发紫<br>- 肋骨之间或之上的皮肤每次呼吸都会被吸入或拉入
. If you have a severe headache and are sick	• 出现严重头痛并感到恶心
. If you have a tight chest, chest pain,	• 出现胸闷、胸痛
. If you have difficulty swallowing or are drooling	• 出现吞咽困难或流口水
. If you cough up blood	• 出现咳血
. If you are feeling a lot worse	• 如果你感觉越来越差了
. If there is no improvement within (only one option will apply) – please note that most symptoms will be better before the listed length, but some can take this long and still get better without treatment<br>1. Acute upper respiratory tract infection – 14 days<br>2. Acute Tonsillitis / Pharyngitis – 8 days<br>3. Acute ear inflammation / Otitis Media – 8 days<br>4. Acute sinusitis – 21 days<br>5. Acute Tracheobronchitis – 21 days<br>6. Pneumonia – 1 week after treatment started	• 如果在正常恢复期内没有改善（每位患者只选一项）-请注意，大多数症状会提前好转，但是有些仍可能会持续到以下时间直至在没有治疗的情况下好转<br>1.急性上呼吸道感染 - 14 天<br>2.急性扁桃体炎/咽炎 - 8 天<br>3.急性耳朵炎症/中耳炎 - 8 天<br>4.急性鼻窦炎 - 21 天<br>5.急性气管支气管炎 - 21 天<br>6. 肺炎 - 已经接受治疗 1 周以后

For peer review only

Participant ID: ________________

WP1 Clinical consultation doctor questionnaire

These questions must be answered by the **doctor** or by direct observation by the field worker

这部分问题要求**医生**回答或者通过调查员的观察完成 Consultation start time: __ h __ min (24-h clock)

开始接诊时间 ____________时_______分（24 小时制）

1. Whether practitioner has checked with patient's symptoms? 医生是否询问症状

☐ Yes 是
☐ No 否

1.1 What are the patient's symptoms? 病人当前有何症状?

☐ Blocked/ runny nose	☐ 鼻塞/流涕
☐ Blocked nose<br>☐ Runny nose (clear/watery discharge)<br>☐ Snotty nose (yellow/green discharge)	☐ 鼻子不通<br>☐ 流清水鼻涕<br>☐ 流脓鼻涕 (黄/绿色鼻涕)
☐ Coughing	☐ 咳嗽
☐ Dry cough<br>☐ Cough with white sputum<br>☐ Cough with yellow/green sputum	☐ 干咳<br>☐ 咳嗽带白色痰<br>☐ 咳嗽带黄/绿色脓痰
☐ Throat problems	☐ 嗓子不舒服
☐ Sore throat<br>☐ Hyperemia/ swelling in throat<br>☐ Hyperemia/ enlargement of tonsils<br>☐ Pus on the tonsils<br>☐ Swollen lymph glands	☐ 咽痛<br>☐ 咽喉部充血/水肿<br>☐ 扁桃体充血/肿大<br>☐ 扁桃体化脓<br>☐ 下颌淋巴结肿大
☐ Breathing	☐ 呼吸
☐ Short of breath<br>☐ Tight chest<br>☐ Difficulty breathing<br>☐ Must sit erect to breathe<br>☐ Wet crackling in lungs	☐ 气促<br>☐ 胸闷<br>☐ 呼吸不畅/困难<br>☐ 端坐呼吸<br>☐ 肺部湿罗音
☐ Ear symptoms	☐ 耳部症状
☐ Blocked ears<br>☐ Tinnitus<br>☐ Pus/ fluid secretion<br>☐ Earache<br>☐ Loss of hearing	☐ 耳闷<br>☐ 耳鸣<br>☐ 流脓/液<br>☐ 耳痛<br>☐ 听力下降
☐ Fever	☐ 发热

For peer review only

Participant ID: ________________

☐ Low 37.3-38 ºC<br>☐ Moderate 38.1-39 ºC<br>☐ High >39 ºC	☐ 低热 37.3-38 摄氏度<br>☐ 中等度热 38.1-39 摄氏度<br>☐ 高热 39 摄氏度以上
☐ Pain	☐ 疼痛
☐ Headache<br>☐ Aching all-over<br>☐ Chest pain	☐ 头痛<br>☐ 全身酸痛<br>☐ 胸痛

Any other symptoms? If yes please state ______________________

除了以上症状，你还有其他不舒服的症状么？ ____________________________

1.2 What kinds of tests were ordered by practitioners? (Please tick and/or write down) （医生给病人开具了哪些检查，请选择或具体填写）

☐Blood test （血液检查）

☐X-Ray （X 射线）

☐CT （CT）

☐B Ultrasound (超声波诊断，B 超)

☐ECG （心电图检查）

☐None （没有）

Other (Please Specify) （其他的，请描述） _______________________

1.3 What kinds of tests were carried out by patient? (Please tick and/or write down) （病人实际做了哪些检查，请选择或具体填写）

☐Blood test （血液检查）

☐X-Ray （X 射线）

☐CT （CT）

☐B Ultrasound (超声波诊断，B 超)

☐ECG （心电图检查）

☐None （没有）

Other (Please Specify) _ （其他的，请描述） ______________________

1.4 Test results （检查结果）

Record practitioners' statement about test results as told to the patient, in his/her own words. If no test result is stated, then write down 'not stated'. 如果医生有告知病人的检查结果，请原话记录医生的描述。如果检查结果没有告知，请填写"未告知"。

____

For peer review only - http://bmjopen.bmj.com/site/about/guidelines.xhtml

Participant ID: ________________

Diagnosis & Treatment 诊断和治疗

2. Whether practitioner has informed patient of the diagnosis?医生是否告知患者诊断

☐ Yes 是
☐ No 否

Tick one 选一种	**2.1 What is the diagnosis? (ask doctor to pick closest category from list of 6 RTI)**	**2.1 诊断是什么？（请医生在六类呼吸系统感染中选择最接近诊断结果的一项）**
	1. Acute upper respiratory tract infection including:<br>. Infectious cold / Acute rhinitis / upper respiratory tract catarrh<br>. Acute inflammation of the throat<br>. Acute viral pharyngitis<br>. Acute viral laryngitis	急性上呼吸道感染：<br>· 传染性感冒/急性鼻炎/上呼吸道粘膜炎<br>· 喉咙急性炎症<br>· 急性病毒性咽炎<br>· 急性病毒性喉炎
	2. Acute tonsil inflammation / Tonsillitis / Pharyngitis	急性化脓性扁桃体炎/ 扁桃体炎/咽炎
	3. Acute ear inflammation / Otitis Media	急性耳朵炎症/中耳炎
	4. Acute sinusitis	急性鼻窦炎
	5. Acute tracheal/ bronchial inflammation / Acute Tracheobronchitis / Bronchitis / Lower respiratory tract infection	急性气管/支气管炎症/急性气管支气管炎/支气管炎/下呼吸道感染
	6. Pneumonia	肺炎
	. Other please state [free text box]	其他请说明[自由文本框]

**2.2 One a scale of 0 to 10, how sick do you think this patient is? (where 10 is very sick and 0 is not sick)**	**2.2 在 1 到 6 的程度中，你认为这位病人有多不舒服？（6 表示非常不舒服， 1 表示一点点不舒服）**
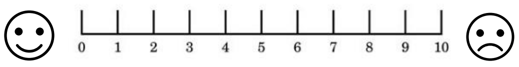	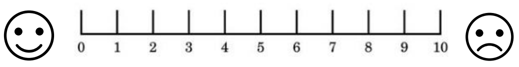

Whether practitioner has informed patient with medication? 医生是否告知患者用药情况

☐ Yes 是
☐ No 否

**2.3 Were any antibiotics prescribed?**	**2.3 抗生素是否开具？**
☐ Yes<br>☐ No	☐ 是<br>☐ 否

Participant ID: ___________________

**If Yes, first tick the method of drug administration; then specify** the name, total quantity and dosage in the table<br><br>☐ IV Antibiotics prescribed<br>☐ Oral antibiotics prescribed	**如果是，请先勾选抗生素的给药方式；再在下表中具体说明**药物通用名，总量和剂量<br><br>☐ 静脉注射抗生素<br>☐ 口服抗生素

2.4 Whether practitioner has informed patient with risks of antibiotics treatment 是否告知患者使用抗菌药物的危害

☐ Yes 告知
☐ No 未告知

IV Antibiotics prescribed:

**Tick if prescribed**	**Medicine Name**	**Specification**	**Total quantity**	**Dosage**
	Penicillin			
	Levofloxacin			
	Ampicillin			
	Clindamycin			
	Amikacin			
	Azithromycin			
	Metronidazole			
	Fosfomycin sodium			
	Amoxicillin			
	Erythromycin			
	Norfloxacin			
	Cefradine			

静脉注射抗生素：

如果有，请勾选	药物名称	规格	总量	剂量
	青霉素			
	左氧氟沙星			
	氨苄青霉素			
	克林霉素			
	阿米卡星(丁胺卡那霉素)			
	阿奇霉素			
	甲硝唑			
	磷霉素钠			
	阿莫西林			
	红霉素			

For peer review only

Participant ID: ___________________

	诺氟沙星			
	头孢拉定			

Oral antibiotics prescribed:

Tick if prescribed	Antibiotics Name	Specification	Total quantity	Dosage
	Amoxicillin			
	Erythromycin			
	Norfloxacin			
	Cefradine			

口服抗生素：

如果有，请勾选	药物名称	规格	总量	剂量
	阿莫西林			
	红霉素			
	诺氟沙星			
	头孢拉定			

Other medicines prescribed:

Tick if prescribed	Medicine Name	Specification	Total quantity	Dosage	Mode of administration
	Dexamethasone				
	Tetracaine				
	chlorpheniramine				
	Aminophylline				
	Ribavirin				
	Compound paracetamol amine capsules				
	Ambroxol				
	Asthma-calming and cough-stopping patch (*pingchuan zhike pian*)				
	Qing kailing(qinghai ling)				
	Galculus Bovis and Metronidazole Capsules				

For peer review only

Participant ID: ___________________

	(*rengong niuhuang jia xiaocuo jiaonang*:				

其它开具的药物：

如果有，请勾选	药物名称	规格	总数量	剂量	服药方式（吊水/口服/贴等）
	地塞米松				
	丁卡因				
	扑尔敏				
	氨茶碱				
	利巴韦林				
	复方氨酚胶囊				
	盐酸氨溴索				
	平喘止咳贴片				
	清开灵				
	人工牛黄甲硝唑胶囊				

**2.4 Did the patient ask for any of these treatments?**	**2.4 患者是否主动要求过以下治疗？**
☐ IV	☐ 输液
☐ Anti-inflammation medicine/antibiotics	☐ 抗生素/消炎药
☐ Other medicines	☐ 其他药物

Any further observation note of the consultation process (Researcher's observation):	其它有关诊疗咨询过程的观察记录？

For peer review only

Participant ID: ________________

Consultation end time: ___ h ___min (24-h clock)

结束接诊时间 _____________时_______分_（24 小时制）

Whether practitioner has informed patient with standard precautions in health care ? 医生是否向患者做疾病注意事项相关宣教

- ☐ Yes 是
- ☐ No 否

Whether practitioner has informed patient with standard precautions in infection control? 医生是否想患者做预防感染相关宣教?

- ☐ Yes 是
- ☐ No 否

For peer review only

Participant ID: ___________________

WP1 Clinical consultation patient questionnaire

A field worker will administer this questionnaire at the clinical consultation at which the patient is recruited at both base line and follow up audit.

These questions must be answered by the **patient** or by direct observation by the field worker

这部分问题要求**病人**回答或者通过调查员的观察完成

1. Eligibility criteria 入选条件

Does patient have a suspected RTI?<br>. Yes<br>. No – exclude from study	病人是否疑似呼吸系统感染？<br>· 是<br>· 否—从本研究中排除
Is patient aged 18 years or older?<br>. Yes<br>. No – exclude from study	病人是否年满 18 岁？<br>· 是<br>· 否—从本研究中排除
Is today the first time the patient has sought treatment for this this illness or a repeat visit?<br>. First time consultation<br>. Repeat consultation – exclude from study	今天是病人首次还是再次为这个病来这里就诊？<br>· 首次就诊<br>· 再次就诊—从本研究中排除

Date completed: __________	调查日期： ______年____月____日
Name of field worker completing proforma: __________	调查员姓名： ________________

For peer review only

Participant ID: ___________________

2. Participant Characteristics 研究对象基本情况

**2.1 Gender**<br>☐ Female<br>☐ Male	**2.1 性别:**<br>☐ 女性<br>☐ 男性
**2.2 Date of Birth**<br>**(Year/Month/Day)**	**2.2 生日**<br>_______年____月____日
**2.3 How many years of education have you completed?**<br>__________________years<br>*[Write down the exact number]*	**2.3 你上了几年学?**<br>_______年<br>(写下确切的数字)

**2.4 Where do you live?**<br>*Name of town or village:*<br>____	**2.4 你住在哪里?**<br>乡镇/村的名字<br>___________________________________
**2.5 If you have lived outside your usual home residence for more than a month over the past 12 months, how many months have you been away?**<br>_________months	**2.5 在过去一年，如果你离开家在外生活超过 1 个月，请给出你具体离家几个月?**<br>_________个月
**2.6Is the place where you have been living urban or rural?**<br>☐ Urban<br>☐ Rural<br>☐ Not applicable	**2.7 你在外生活的地方是农村还是城市**<br>☐ 城市<br>☐ 农村<br>☐ 不适用
**2.7What is your Household registration status (*hukou*) for accessing health care benefits?**<br>☐ Urban<br>☐ Rural<br>☐ Other______________________	**2.8 目前跟你医保挂钩的是什么户口?**<br>☐ 城市<br>☐ 农村<br>☐ 其他______________________
**2.8What type of insurance do you have, if any?**<br>☐ New rural cooperative Medicalinsurance scheme<br>☐ Urban Employee's Medical Insurance<br>☐ Urban Residents' Medical Insurance<br>☐ Not Having Medical Insurance<br>☐ Other Type of Insurance *[please specify]*:<br>_____________________________	**2.9 你参加了哪些医疗保险?**<br>☐ 新型农村合作医疗<br>☐ 城镇职工医疗保险<br>☐ 城镇居民医疗保险<br>☐ 没有医疗保险<br>☐ 其他医疗保险（请注明）：<br>____________________________

For peer review only

Participant ID: ________________

3. Duration of illness and the severity 患病时长和严重程度

**Duration of your illness: _____ days**	**你的病已经持续了_______天**
**One a scale of 0 to 10, how sick do you feel? (where 10 is very sick and 0 is not sick)**	**在 0 到 10 的程度中，你认为你有多不舒服？（10 表示非常不舒服， 0 表示没有不舒服）**
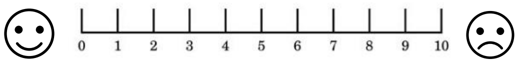	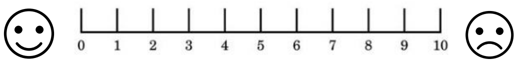

Only complete these questions if the symptoms have not been recorded on the clinician questionnaire.

请仅在医生问卷没有收集到下列症状的情况下，向病人询问下列问题。

What are yoursymptoms?您当前有何症状?

☐Blocked/ runny nose	☐鼻塞/流涕
☐ Blocked nose<br>☐ Runny nose (clear/watery discharge)<br>☐ Snotty nose (yellow/green discharge)	☐ 鼻子不通<br>☐ 流清水鼻涕<br>☐ 流脓鼻涕(黄/绿色鼻涕)
☐Coughing	☐咳嗽
☐ Dry cough<br>☐ Cough with white sputum<br>☐ Cough with yellow/green sputum	☐ 干咳<br>☐ 咳嗽带白色痰<br>☐ 咳嗽带黄/绿色脓痰
☐Throat problems	☐嗓子不舒服
☐Breathing	☐呼吸
☐ Short of breath<br>☐ Tight chest<br>☐ Difficulty breathing<br>☐ Must sit erect to breathe	☐ 气促<br>☐ 胸闷<br>☐ 呼吸不畅/困难<br>☐ 端坐呼吸<br>☐
☐Earsymptoms	☐耳部症状
☐ Blocked ears<br>☐ Tinnitus<br>☐ Pus/ fluid secretion<br>☐ Earache<br>☐ Loss of hearing	☐ 耳闷<br>☐ 耳鸣<br>☐ 流脓/液<br>☐ 耳痛<br>☐ 听力下降
☐Fever	☐发热
☐Pain	☐疼痛
☐ Headache<br>☐ Aching all-over<br>☐ Chest pain	☐ 头痛<br>☐ 全身酸痛<br>☐ 胸痛

For peer review only

Participant ID: ________________

除了以上症状，你还有其他不舒服的症状么？ ___________________________

Alongside above symptoms, did you have any other symptoms that make you unwell? ______

4. Medication （用药情况）

1	2	3	4	5
**Have you taken any medication for this illness before coming here? Yes/No** （在为这个病来就诊前，你有服用任何药物么？<br><br>**If yes, please answer column 2 and 3**<br>如果回答 是， 请回答问题 2 和 3<br><br>**If No, please answer column 4 and 5**<br><br>如果回答 没有，请回答问题 4 和 5	**Days have you taken it?** （你服用药物几天？）	**Where did you got it?** （你从那里获得服用的药物？）<br>1, pharmacies outside the hospitals （医院外的药房）<br>2, family or relatives or friends （家人和朋友给的）<br>3 Residual drugs at home （之前用药剩下的）	**Have you had the medication in the past six month before this illness?** （在这个病的前 6 个月里，你有服用任何药物么？<br>**Yes/No 是/**否<br>**If yes, please answer column 5** （回答是，请继续回答问题 5）	**How many course have you taken?** （你服用几个疗程的药物）<br><br>1,1-3 times<br>1-3 个疗程<br>2, 4-6 times<br>4-6 个疗程<br>3, 6 times<br>6 个疗程<br>4, more than 6 times<br>超过 6 个疗程

For peer review only

5.Patient Satisfaction 患者满意度

**One a scale of 0 to 10, how satisfied were you with your visit to the doctor today? (where 10 is very satisfied and 0 is not satisfied at all)**	**在 0 到 10 的程度中，你今天在这里看病满意吗？(10 表示非常满意， 0 表示完全不满意)**
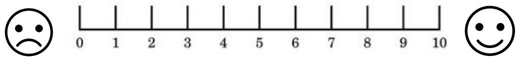	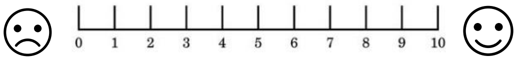

Participant ID: ___________________

Health Questionnaire

English version for the UK

VERSION FOR INTERVIEWER ADMINISTRATION

Note to interviewer: although allowance should be made for the interviewer's particular style of speaking, the wording of the questionnaire instructions should be followed as closely as possible. In the case of the EQ-5D-5L descriptive system on page 2 of the questionnaire, the precise wording must be followed.

If the respondent has difficulty choosing a response, or asks for clarification, the interviewer should repeat the question word for word and ask the respondent to answer in a way that most closely resembles his or her thoughts about his or her health today.

INTRODUCTION

(Note to interviewer: please read the following to the respondent.)

We are trying to find out what you think about your health. I will explain what to do as I go along, but please interrupt me if you do not understand something or if things are not clear to you. There are no right or wrong answers. We are interested only in your personal view.

First, I am going to read out some questions. Each question has a choice of five answers. Please tell me which answer best describes your health TODAY.

Do not choose more than one answer in each group of questions.

For peer review only

Participant ID: ________________

(Note to interviewer: first read all five options for each question. Then ask the respondent to choose which one applies to him/herself. Repeat the question and options if necessary. Mark the appropriate box under each heading. You may need to remind the respondent regularly that the timeframe is TODAY.)

EQ-5D DESCRIPTIVE SYSTEM

MOBILITY

First, I would like to ask you about mobility. Would you say that:

1. You have no problems in walking about? ❑
2. You have slight problems in walking about? ❑
3. You have moderate problems in walking about? ❑
4. You have severe problems in walking about? ❑
5. You are unable to walk about? ❑

SELF-CARE

Next, I would like to ask you about self-care. Would you say that:

1. You have no problems washing or dressing yourself? ❑
2. You have slight problems washing or dressing yourself? ❑
3. You have moderate problems washing or dressing yourself? ❑
4. You have severe problems washing or dressing yourself? ❑
5. You are unable to wash or dress yourself? ❑

USUAL ACTIVITIES

Next, I would like to ask you about usual activities, for example work, study, housework, family or leisure activities. Would you say that:

1. You have no problems doing your usual activities? ❑
2. You have slight problems doing your usual activities? ❑
3. You have moderate problems doing your usual activities? ❑
4. You have severe problems doing your usual activities? ❑
5. You are unable to do your usual activities? ❑

PAIN / DISCOMFORT

Next, I would like to ask you about pain or discomfort. Would you say that:

1. You have no pain or discomfort? ❑
2. You have slight pain or discomfort? ❑
3. You have moderate pain or discomfort? ❑

For peer review only

Participant ID: ___________________

4. You have <u>severe</u> pain or discomfort? ☐
5. You have <u>extreme</u> pain or discomfort? ☐

The best health you can imagine

ANXIETY / DEPRESSION

Finally, I would like to ask you about anxiety or depression. Would you say that:

1. You are <u>not</u> anxious or depressed? ☐
2. You are <u>slightly</u> anxious or depressed? ☐
3. You are <u>moderately</u> anxious or depressed? ☐
4. You are <u>severely</u> anxious or depressed? ☐
5. You are <u>extremely</u> anxious or depressed? ☐

EQ-5D VAS

- **Now, I would like to ask you to say how good or bad your health is TODAY.**

- **I would like you to try to picture in your mind a scale that looks like a thermometer.**
  (Note to interviewer: if interviewing face-to-face, please show the person the VAS scale.)

- **The best health you can imagine is marked 100 (one hundred) at the top of the scale and the worst health you can imagine is marked 0 (zero) at the bottom.**

- **I would now like you to tell me the point on this scale where you would put your health TODAY.**
  (Note to interviewer: mark the scale at the point indicating the respondent's 'health today'. Now, please write the number you marked on the scale in the box below.)

For peer review only

Participant ID: ___________________

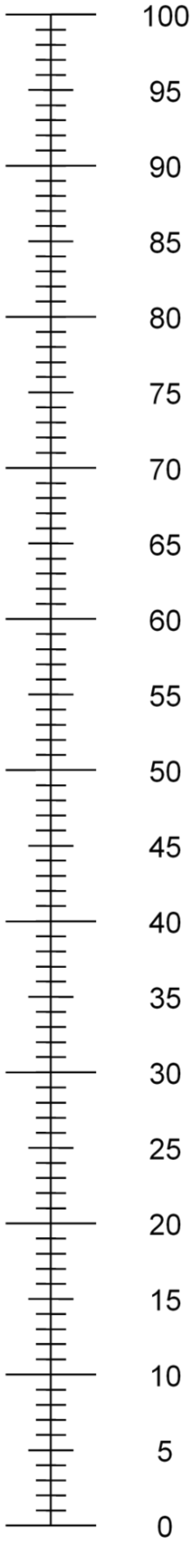

THE RESPONDENT’S HEALTH TODAY = ☐

Thank you for taking the time to answer these questions.

The worst health you can imagine

For peer review only

WP1 Patient telephone questionnaire – Day 7

A field worker will conduct phone interviews with the patients at 7, 14 and 21 days. Patients will also be asked the questions from the EQ5D.

调查员将在第 7， 14 和 21 天对病人进行电话访谈。除了以下问题，病人还会被问道一些 EQ5D 问卷中的问题。

1.Basic information 基本情况

**Patient ID**	**病人编号**
Days since baseline consultation<br>☐ 7 days<br>☐ 14 days<br>☐ 21 days	这是最初看病之后的第几天?<br>☐ 7 天<br>☐ 14 天<br>☐ 21 天
Date completed: __________	调查日期： ______年____月____日
Name of field worker completing proforma:<br>__________	调查员姓名： ________________

2. Continuing Illness Severity 疾病持续严重程度

**One a scale of 0 to 10, how sick do you feel? (where 10 is very sick and 0 is not sick)**	在 **0** 到 **10** 的程度中，你认为你有多不舒服？ **(10** 表示非常不舒服， **0** 表示没有不舒服**)**
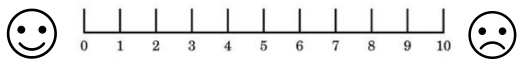	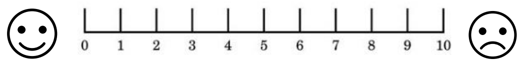

3. Patient view of leaflet 病人对宣传手册的看法

Did you have a look of the patient leaflet?<br>☐ Yes<br>☐ No<br>☐ Did not get leaflet	你是否看了病人宣传手册?<br>☐ 是<br>☐ 否<br>☐ 没有拿到
How easy was the patient leaflet to understand?<br>☐ Very easy, I understood all of it<br>☐ Easy, I understood most of it<br>☐ Not Easy, I only undersood a little<br>☐ Too hard, I did not understand any<br>☐ I am not able to read	你觉得病人宣传手册有多容易理解?<br>☐ 非常容易，我可以全部理解<br>☐ 容易，我可以理解大部分<br>☐ 不容易，我只能理解小部分<br>☐ 非常难，我完全不能理解<br>☐ 我读不了宣传手册

For peer review only - http://bmjopen.bmj.com/site/about/guidelines.xhtml

Did the leaflet change your ideas or beliefs about what illnesses need anti-inflammation medicine/ antibiotics?<br>☐ Yes<br>☐ No	病人宣传手册对您关于什么样的<br>疾病需要使用消炎药/抗生素的想法有改变吗?<br>☐ 有<br>☐ 没有

For peer review only

4. Antibiotic Consumption 抗生素服用情况

Were you prescribed oral anti-bacterial/anti-inflammation medicine in your baseline consultation?<br>☐ Yes (if yes, field worker will ask the two questions below in this table)<br>☐ No<br>☐ Not applicable	您在最初看病时是否有开口服抗菌药物/消炎药吗？<br>☐ 是（如果有，请继续问下面两个问题）<br>☐ 否<br>☐ 不清楚
How many days and times per day did you actually take a dose since you saw the Dr?<br>☐ [Number] ________of days<br>☐ [Number]________ times / day	从那之后你已经吃了多少天？每天实际吃多少次？<br>☐ （ ）天<br>☐ （ ）次/天
What was the dose did you take every time?	您每次吃的剂量是多少？
Have you stopped taking the anti-bacterial/anti-inflammation medicine that you were prescribed?<br>☐ Yes<br>☐ No<br>☐ Not applicable	你是否已经停止服用给你开的抗菌药物/消炎药了？<br>☐ 是<br>☐ 否<br>☐ 不清楚

For peer review only

For peer review only - http://bmjopen.bmj.com/site/about/guidelines.xhtml

Health Questionnaire

English version for the UK

VERSION FOR INTERVIEWER ADMINISTRATION

Note to interviewer: although allowance should be made for the interviewer's particular style of speaking, the wording of the questionnaire instructions should be followed as closely as possible. In the case of the EQ-5D-5L descriptive system on page 2 of the questionnaire, the precise wording must be followed.

If the respondent has difficulty choosing a response, or asks for clarification, the interviewer should repeat the question word for word and ask the respondent to answer in a way that most closely resembles his or her thoughts about his or her health today.

INTRODUCTION

(Note to interviewer: please read the following to the respondent.)

We are trying to find out what you think about your health. I will explain what to do as I go along, but please interrupt me if you do not understand something or if things are not clear to you. There are no right or wrong answers. We are interested only in your personal view.

First, I am going to read out some questions. Each question has a choice of five answers. Please tell me which answer best describes your health TODAY.

Do not choose more than one answer in each group of questions.

(Note to interviewer: first read all five options for each question. Then ask the respondent to choose which one applies to him/herself. Repeat the question and options if necessary. Mark the appropriate box under each heading. You may need to remind the respondent regularly that the timeframe is TODAY.)

For peer review only - http://bmjopen.bmj.com/site/about/guidelines.xhtml

EQ-5D DESCRIPTIVE SYSTEM

MOBILITY

First, I would like to ask you about mobility. Would you say that:

1. You have no problems in walking about? ❑
2. You have slight problems in walking about? ❑
3. You have moderate problems in walking about? ❑
4. You have severe problems in walking about? ❑
5. You are unable to walk about? ❑

SELF-CARE

Next, I would like to ask you about self-care. Would you say that:

1. You have no problems washing or dressing yourself? ❑
2. You have slight problems washing or dressing yourself? ❑
3. You have moderate problems washing or dressing yourself? ❑
4. You have severe problems washing or dressing yourself? ❑
5. You are unable to wash or dress yourself? ❑

USUAL ACTIVITIES

Next, I would like to ask you about usual activities, for example work, study, housework, family or leisure activities. Would you say that:

1. You have no problems doing your usual activities? ❑
2. You have slight problems doing your usual activities? ❑
3. You have moderate problems doing your usual activities? ❑
4. You have severe problems doing your usual activities? ❑
5. You are unable to do your usual activities? ❑

PAIN / DISCOMFORT

Next, I would like to ask you about pain or discomfort. Would you say that:

1. You have no pain or discomfort? ❑
2. You have slight pain or discomfort? ❑
3. You have moderate pain or discomfort? ❑
4. You have severe pain or discomfort? ❑
5. You have extreme pain or discomfort? ❑

ANXIETY / DEPRESSION

Finally, I would like to ask you about anxiety or depression. Would you say that:

1. You are not anxious or depressed? ❑
2. You are slightly anxious or depressed? ❑
3. You are moderately anxious or depressed? ❑
4. You are severely anxious or depressed? ❑
5. You are extremely anxious or depressed? ❑

For peer review only

For peer review only - http://bmjopen.bmj.com/site/about/guidelines.xhtml

EQ-5D VAS

- **Now, I would like to ask you to say how good or bad your health is TODAY.**
- **I would like you to try to picture in your mind a scale that looks like a thermometer.**
  (Note to interviewer: if interviewing face-to-face, please show the person the VAS scale.)
- **The best health you can imagine is marked 100 (one hundred) at the top of the scale and the worst health you can imagine is marked 0 (zero) at the bottom.**
- **I would now like you to tell me the point on this scale where you would put your health TODAY.**
  (Note to interviewer: mark the scale at the point indicating the respondent's 'health today'. Now, please write the number you marked on the scale in the box below.)

THE RESPONDENT'S HEALTH TODAY = [ ]

Thank you for taking the time to answer these questions.

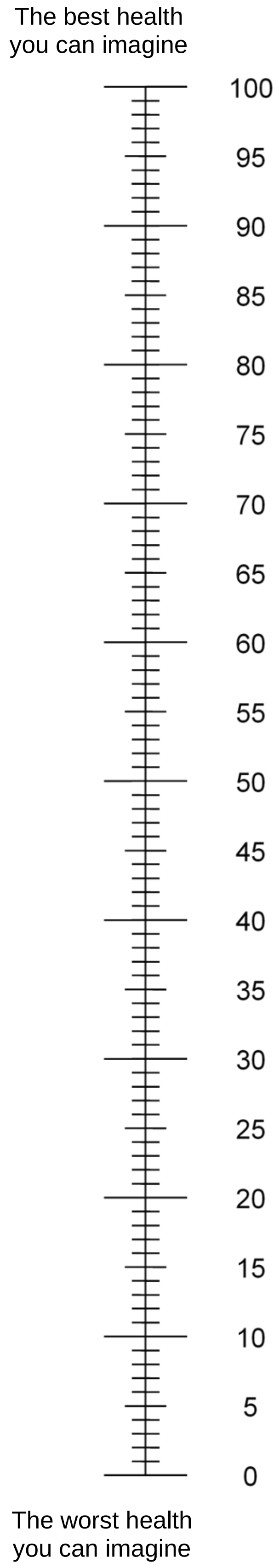

For peer review only

WP1 Patient telephone questionnaire – Day 14

A field worker will conduct phone interviews with the patients at 7, 14 and 21 days. Patients will also be asked the questions from the EQ5D.

调查员将在第 7， 14 和 21 天对病人进行电话访谈。除了以下问题，病人还会被问道一些 EQ5D 问卷中的问题。

1.Basic information 基本情况

**Patient ID**	**病人编号**
Days since baseline consultation<br>☐ 7 days<br>☐ 14 days<br>☐ 21 days	这是最初看病之后的第几天？<br>☐ 7 天<br>☐ 14 天<br>☐ 21 天
Date completed: __________	调查日期： ______年____月____日
Name of field worker completing proforma:<br>__________	调查员姓名： _______________

2. Continuing Illness Severity 疾病持续严重程度

**One a scale of 0 to 10, how sick do you feel? (where 10 is very sick and 0 is not sick)**	**在 0 到 10 的程度中，你认为你有多不舒服？（10 表示非常不舒服， 0 表示没有不舒服）**
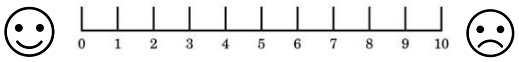 	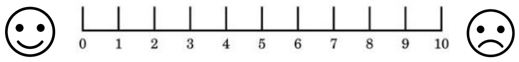

3. Antibiotic Consumption 抗生素服用情况

Were you prescribed oral anti-bacterial/anti-inflammation medicine in your baseline consultation?<br>☐ Yes (if yes, field worker will ask the two questions below in this table)<br>☐ No<br>☐ Not applicable	您在最初看病时是否有开口服抗菌药物/消炎药吗？<br>☐ 是（如果有，请继续问下面两个问题）<br>☐ 否<br>☐ 不清楚
How many days and times per day did you actually take a dose since you saw the Dr?<br>☐ [Number] _________of days<br>☐ [Number]_________ times / day	从那之后你已经吃了多少天？每天实际吃多少次？<br>☐ （ ） 天<br>☐ （ ） 次/天
What was the dose did you take every time?	您每次吃的剂量是多少？

For peer review only - http://bmjopen.bmj.com/site/about/guidelines.xhtml

Have you stopped taking the anti-bacterial/anti-inflammation medicine that you were prescribed?<br><br>☐ Yes<br>☐ No<br>☐ Not applicable	你是否已经停止服用给你开的抗菌药物/消炎药了？<br><br>☐ 是<br>☐ 否<br>☐ 不清楚

For peer review only

EQ-5D-5L

Health Questionnaire

English version for the UK

VERSION FOR INTERVIEWER ADMINISTRATION

Note to interviewer: although allowance should be made for the interviewer's particular style of speaking, the wording of the questionnaire instructions should be followed as closely as possible. In the case of the EQ-5D-5L descriptive system on page 2 of the questionnaire, the precise wording must be followed.

If the respondent has difficulty choosing a response, or asks for clarification, the interviewer should repeat the question word for word and ask the respondent to answer in a way that most closely resembles his or her thoughts about his or her health today.

INTRODUCTION

(Note to interviewer: please read the following to the respondent.)

We are trying to find out what you think about your health. I will explain what to do as I go along, but please interrupt me if you do not understand something or if things are not clear to you. There are no right or wrong answers. We are interested only in your personal view.

First, I am going to read out some questions. Each question has a choice of five answers. Please tell me which answer best describes your health TODAY.

Do not choose more than one answer in each group of questions.

(Note to interviewer: first read all five options for each question. Then ask the respondent to choose which one applies to him/herself. Repeat the question and options if necessary. Mark the appropriate box under each heading. You may need to remind the respondent regularly that the timeframe is TODAY.)

For peer review only - http://bmjopen.bmj.com/site/about/guidelines.xhtml

EQ-5D DESCRIPTIVE SYSTEM

MOBILITY

First, I would like to ask you about mobility. Would you say that:

1. You have no problems in walking about? ❑
2. You have slight problems in walking about? ❑
3. You have moderate problems in walking about? ❑
4. You have severe problems in walking about? ❑
5. You are unable to walk about? ❑

SELF-CARE

Next, I would like to ask you about self-care. Would you say that:

1. You have no problems washing or dressing yourself? ❑
2. You have slight problems washing or dressing yourself? ❑
3. You have moderate problems washing or dressing yourself? ❑
4. You have severe problems washing or dressing yourself? ❑
5. You are unable to wash or dress yourself? ❑

USUAL ACTIVITIES

Next, I would like to ask you about usual activities, for example work, study, housework, family or leisure activities. Would you say that:

1. You have no problems doing your usual activities? ❑
2. You have slight problems doing your usual activities? ❑
3. You have moderate problems doing your usual activities? ❑
4. You have severe problems doing your usual activities? ❑
5. You are unable to do your usual activities? ❑

PAIN / DISCOMFORT

Next, I would like to ask you about pain or discomfort. Would you say that:

1. You have no pain or discomfort? ❑
2. You have slight pain or discomfort? ❑
3. You have moderate pain or discomfort? ❑
4. You have severe pain or discomfort? ❑
5. You have extreme pain or discomfort? ❑

ANXIETY / DEPRESSION

Finally, I would like to ask you about anxiety or depression. Would you say that:

1. You are not anxious or depressed? ❑
2. You are slightly anxious or depressed? ❑
3. You are moderately anxious or depressed? ❑
4. You are severely anxious or depressed? ❑
5. You are extremely anxious or depressed? ❑

For peer review only

For peer review only - http://bmjopen.bmj.com/site/about/guidelines.xhtml

EQ-5D VAS

- **Now, I would like to ask you to say how good or bad your health is TODAY.**
- **I would like you to try to picture in your mind a scale that looks like a thermometer.**
  (Note to interviewer: if interviewing face-to-face, please show the person the VAS scale.)
- **The best health you can imagine is marked 100 (one hundred) at the top of the scale and the worst health you can imagine is marked 0 (zero) at the bottom.**
- **I would now like you to tell me the point on this scale where you would put your health TODAY.**
  (Note to interviewer: mark the scale at the point indicating the respondent's 'health today'. Now, please write the number you marked on the scale in the box below.)

THE RESPONDENT'S HEALTH TODAY = ☐

Thank you for taking the time to answer these questions.

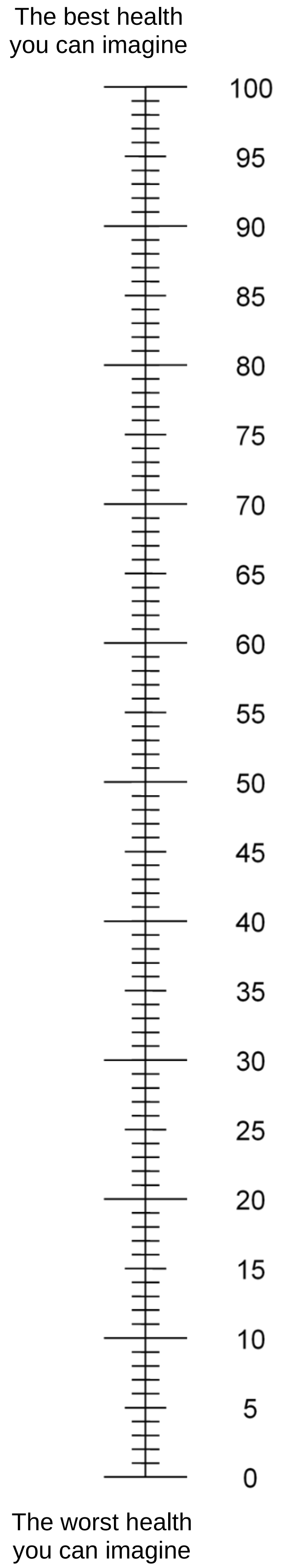

For peer review only

For peer review only - http://bmjopen.bmj.com/site/about/guidelines.xhtml

WP1 Patient telephone questionnaire – Day 21

A field worker will conduct phone interviews with the patients at 7, 14 and 21 days. Patients will also be asked the questions from the EQ5D.

调查员将在第 7，14 和 21 天对病人进行电话访谈。除了以下问题，病人还会被问道一些 EQ5D 问卷中的问题。

1.Basic information 基本情况

**Patient ID**	**病人编号**
Days since baseline consultation<br>☐ 7 days<br>☐ 14 days<br>☐ 21 days	这是最初看病之后的第几天？<br>☐ 7 天<br>☐ 14 天<br>☐ 21 天
Date completed: __________	调查日期：______年____月____日
Name of field worker completing proforma:<br>__________	调查员姓名：_______________

2. Continuing Illness Severity 疾病持续严重程度

**One a scale of 0 to 10, how sick do you feel? (where 10 is very sick and 0 is not sick)**	**在 0 到 10 的程度中，你认为你有多不舒服？（10 表示非常不舒服， 0 表示没有不舒服）**
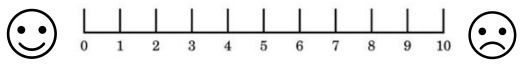	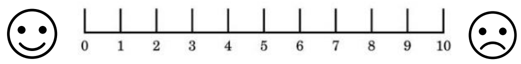

3. Antibiotic Consumption 抗生素服用情况

Were you prescribed oral anti-bacterial/anti-inflammation medicine in your baseline consultation?<br>☐ Yes (if yes, field worker will ask the two questions below in this table)<br>☐ No<br>☐ Not applicable	您在最初看病时是否有开口服抗菌药物/消炎药吗？<br>☐ 是（如果有，请继续问下面两个问题）<br>☐ 否<br>☐ 不清楚
How many days and times per day did you actually take a dose since you saw the Dr?<br>☐ [Number] _________of days<br>☐ [Number]_________ times / day	从那之后你已经吃了多少天？每天实际吃多少次？<br>☐ （ ）天<br>☐ （ ）次/天
Have you stopped taking the anti-	你是否已经停止服用给你开的抗菌药物/消炎

For peer review only - http://bmjopen.bmj.com/site/about/guidelines.xhtml

bacterial/anti-inflammation medicine that you were prescribed?<br>☐ Yes<br>☐ No<br>☐ Not applicable	药了？<br>☐ 是<br>☐ 否<br>☐ 不清楚

4. Resource Use for Economic Evaluation

Additional Health Care Utilisation 其他卫生服务的使用

Did you seek treatment for your illness from any of these since we talked to you in your consultation?	How many times did you visit there?	How much did you pay in total?	How much did you pay from your own pocket?
Pharmacy			
Other township health centre			
Village clinics			
Private clinics			

自从上次看病之后，你是否还因为你的病去下列地方寻医问药?	你去了几次?	总共花了多少钱?	你自费付了多少钱?
药店			
其他乡镇卫生院			
村卫生室			
个体诊所			

Did you seek secondary care for your illness since we talked to you in your consultation?	How many times did you visit?	How much did you pay in total	How much did you pay from your own pocket?
Outpatient			
Inpatient			
Accident and emergency			

自从上次看病之后，你是否因为你的病去医院就诊?	你去了几次?	总共花了多少钱?	你自费付了多少钱?
门诊			
住院			
急诊			

After the consultation when	Prescribed	Bought	Obtained from	How much	How much

For peer review only - http://bmjopen.bmj.com/site/about/guidelines.xhtml

we recruited you, have you had any treatment for this illness that wasn't prescribed in your consultation?	by other doctors	from pharmacy	other source (home, family, friends)	did you pay in total?	did you pay from your own pocket?
**Anti-bacterial/anti-inflammation medicine**					
☐ Amoxicillin	Y/N	Y/N	Y/N	¥	¥
☐ Erythromycin	Y/N	Y/N	Y/N	¥	¥
☐ Cefradine	Y/N	Y/N	Y/N	¥	¥
☐ Levofloxacin	Y/N	Y/N	Y/N	¥	¥
☐ Cefuroxime	Y/N	Y/N	Y/N	¥	¥
☐ Other anti-bacterial/anti-inflammation medicine [please specify]	Y/N	Y/N	Y/N	¥	¥
**Other medicines/treatment**					
☐ Paracetamol	Y/N	Y/N	Y/N	¥	¥
☐ Ganmaoling Granules （e.g. 999 cold remedy）	Y/N	Y/N	Y/N	¥	¥
☐ Qingkailing	Y/N	Y/N	Y/N	¥	¥
☐ Yinhuang Granules	Y/N	Y/N	Y/N	¥	¥
☐ Xiongju Shangqing	Y/N	Y/N	Y/N	¥	¥
☐ Lianhua (lotus) Qingwen capsules	Y/N	Y/N	Y/N	¥	¥
☐ Cough syrup	Y/N	Y/N	Y/N	¥	¥
☐ Ambroxol Hydrochloride oral suspension	Y/N	Y/N	Y/N	¥	¥
☐ Keteling	Y/N	Y/N	Y/N	¥	¥
☐ Compound liquorice tablets	Y/N	Y/N	Y/N	¥	¥
☐ Vitamin C	Y/N	Y/N	Y/N	¥	¥
☐ Crystal sugar snow pear	Y/N	Y/N	Y/N	¥	¥
☐ Ginger	Y/N	Y/N	Y/N	¥	¥
☐ Mugwort	Y/N	Y/N	Y/N	¥	¥
☐ Pipa (loquat)	Y/N	Y/N	Y/N	¥	¥
☐ Orange peel granules	Y/N	Y/N	Y/N	¥	¥
☐ Other [please specify]	Y/N	Y/N	Y/N	¥	¥

在最初看病之后，你是否自己用过了其他不是这次看病是所开具的药物？	其他医生开具	从药店购买	其他方法获得（家中储存，家人，朋友）	这些药总共多少钱？	你自费付了多少钱？
**抗菌药/消炎药**					
☐ 阿莫西林	是/否	是/否	是/否	¥	¥
☐ 红霉素	是/否	是/否	是/否	¥	¥
☐ 头孢拉定	是/否	是/否	是/否	¥	¥

For peer review only - http://bmjopen.bmj.com/site/about/guidelines.xhtml

☐ 左氧氟沙星	是/否	是/否	是/否	¥	¥
☐ 头孢呋辛	是/否	是/否	是/否	¥	¥
☐ 其他抗菌药/消炎药（请注明）	是/否	是/否	是/否	¥	¥
**其他药物**					
☐ 扑热息痛	是/否	是/否	是/否	¥	¥
☐ 感冒灵颗粒（如 999 感冒灵）	是/否	是/否	是/否	¥	¥
☐ 清开灵	是/否	是/否	是/否	¥	¥
☐ 银黄颗粒	是/否	是/否	是/否	¥	¥
☐ 芎菊上清	是/否	是/否	是/否	¥	¥
☐ 莲花清瘟胶囊	是/否	是/否	是/否	¥	¥
☐ 咳嗽糖浆	是/否	是/否	是/否	¥	¥
☐ 盐酸氨溴索口服液	是/否	是/否	是/否	¥	¥
☐ 咳特灵	是/否	是/否	是/否	¥	¥
☐ 复方甘草片	是/否	是/否	是/否	¥	¥
☐ 维生素 C	是/否	是/否	是/否	¥	¥
☐ 冰糖雪梨	是/否	是/否	是/否	¥	¥
☐ 姜	是/否	是/否	是/否	¥	¥
☐ 艾草	是/否	是/否	是/否	¥	¥
☐ 琵琶	是/否	是/否	是/否	¥	¥
☐ 陈皮颗粒	是/否	是/否	是/否	¥	¥
☐ 其他（请注明）	是/否	是/否	是/否	¥	¥

Productivity loss 生产力下降

**If you work, how many days in total have you taken off work due to this illness?**	如果你工作，那么因为这次生病你总共耽误了几天上班？
{number} days	（ ）天
**If you are in full time education, please state how many days in total have you missed your education?**	如果你是学生，那么因为这次生病你总共耽误了几天上学？
{number} days	（ ）天

For peer review only - http://bmjopen.bmj.com/site/about/guidelines.xhtml

Health Questionnaire

English version for the UK

VERSION FOR INTERVIEWER ADMINISTRATION

Note to interviewer: although allowance should be made for the interviewer's particular style of speaking, the wording of the questionnaire instructions should be followed as closely as possible. In the case of the EQ-5D-5L descriptive system on page 2 of the questionnaire, the precise wording must be followed.

If the respondent has difficulty choosing a response, or asks for clarification, the interviewer should repeat the question word for word and ask the respondent to answer in a way that most closely resembles his or her thoughts about his or her health today.

INTRODUCTION

(Note to interviewer: please read the following to the respondent.)

We are trying to find out what you think about your health. I will explain what to do as I go along, but please interrupt me if you do not understand something or if things are not clear to you. There are no right or wrong answers. We are interested only in your personal view.

First, I am going to read out some questions. Each question has a choice of five answers. Please tell me which answer best describes your health TODAY.

Do not choose more than one answer in each group of questions.

(Note to interviewer: first read all five options for each question. Then ask the respondent to choose which one applies to him/herself. Repeat the question and options if necessary. Mark the appropriate box under each heading. You may need to remind the respondent regularly that the timeframe is TODAY.)

For peer review only - http://bmjopen.bmj.com/site/about/guidelines.xhtml

EQ-5D DESCRIPTIVE SYSTEM

MOBILITY

First, I would like to ask you about mobility. Would you say that:

1. You have no problems in walking about? ❑
2. You have slight problems in walking about? ❑
3. You have moderate problems in walking about? ❑
4. You have severe problems in walking about? ❑
5. You are unable to walk about? ❑

SELF-CARE

Next, I would like to ask you about self-care. Would you say that:

1. You have no problems washing or dressing yourself? ❑
2. You have slight problems washing or dressing yourself? ❑
3. You have moderate problems washing or dressing yourself? ❑
4. You have severe problems washing or dressing yourself? ❑
5. You are unable to wash or dress yourself? ❑

USUAL ACTIVITIES

Next, I would like to ask you about usual activities, for example work, study, housework, family or leisure activities. Would you say that:

1. You have no problems doing your usual activities? ❑
2. You have slight problems doing your usual activities? ❑
3. You have moderate problems doing your usual activities? ❑
4. You have severe problems doing your usual activities? ❑
5. You are unable to do your usual activities? ❑

PAIN / DISCOMFORT

Next, I would like to ask you about pain or discomfort. Would you say that:

1. You have no pain or discomfort? ❑
2. You have slight pain or discomfort? ❑
3. You have moderate pain or discomfort? ❑
4. You have severe pain or discomfort? ❑
5. You have extreme pain or discomfort? ❑

ANXIETY / DEPRESSION

Finally, I would like to ask you about anxiety or depression. Would you say that:

1. You are not anxious or depressed? ❑
2. You are slightly anxious or depressed? ❑
3. You are moderately anxious or depressed? ❑
4. You are severely anxious or depressed? ❑
5. You are extremely anxious or depressed? ❑

For peer review only - http://bmjopen.bmj.com/site/about/guidelines.xhtml

EQ-5D VAS

- **Now, I would like to ask you to say how good or bad your health is TODAY.**
- **I would like you to try to picture in your mind a scale that looks like a thermometer.**
  (Note to interviewer: if interviewing face-to-face, please show the person the VAS scale.)
- **The best health you can imagine is marked 100 (one hundred) at the top of the scale and the worst health you can imagine is marked 0 (zero) at the bottom.**
- **I would now like you to tell me the point on this scale where you would put your health TODAY.**
  (Note to interviewer: mark the scale at the point indicating the respondent's 'health today'. Now, please write the number you marked on the scale in the box below.)

THE RESPONDENT'S HEALTH TODAY = ☐

Thank you for taking the time to answer these questions.

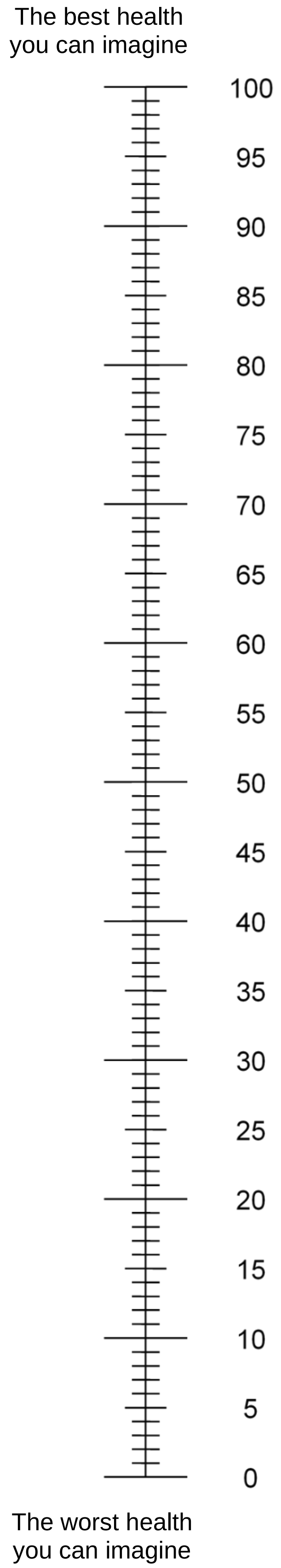

For peer review only

CONSORT 2010 checklist of information to include when reporting a randomised trial*

Section/Topic	Item No	Checklist item	Reported on page No
**Title and abstract**			
	1a	Identification as a randomised trial in the title	Page 2
	1b	Structured summary of trial design, methods, results, and conclusions (for specific guidance see CONSORT for abstracts)	Page 2
**Introduction**			
Background and objectives	2a	Scientific background and explanation of rationale	Page 3 & 4
	2b	Specific objectives or hypotheses	Page 4
**Methods**			
Trial design	3a	Description of trial design (such as parallel, factorial) including allocation ratio	Page 4
	3b	Important changes to methods after trial commencement (such as eligibility criteria), with reasons	Page 4 & 5
Participants	4a	Eligibility criteria for participants	Page 5 & 6
	4b	Settings and locations where the data were collected	Page 5 & 6
Interventions	5	The interventions for each group with sufficient details to allow replication, including how and when they were actually administered	Page 6 & 7
Outcomes	6a	Completely defined pre-specified primary and secondary outcome measures, including how and when they were assessed	Page 8 & 9
	6b	Any changes to trial outcomes after the trial commenced, with reasons	
Sample size	7a	How sample size was determined	Page 9
	7b	When applicable, explanation of any interim analyses and stopping guidelines	
Randomisation:			
Sequence generation	8a	Method used to generate the random allocation sequence	
	8b	Type of randomisation; details of any restriction (such as blocking and block size)	Page 6
Allocation concealment mechanism	9	Mechanism used to implement the random allocation sequence (such as sequentially numbered containers), describing any steps taken to conceal the sequence until interventions were assigned	Page 6
Implementation	10	Who generated the random allocation sequence, who enrolled participants, and who assigned participants to interventions	Page 6
Blinding	11a	If done, who was blinded after assignment to interventions (for example, participants, care providers, those	Page 6

For peer review only - http://bmjopen.bmj.com/site/about/guidelines.xhtml

		assessing outcomes) and how	
	11b	If relevant, description of the similarity of interventions	
Statistical methods	12a	Statistical methods used to compare groups for primary and secondary outcomes	Page 8 & 9
	12b	Methods for additional analyses, such as subgroup analyses and adjusted analyses	Page 9
**Results**			
Participant flow (a diagram is strongly recommended)	13a	For each group, the numbers of participants who were randomly assigned, received intended treatment, and were analysed for the primary outcome	See figure 1
	13b	For each group, losses and exclusions after randomisation, together with reasons	
Recruitment	14a	Dates defining the periods of recruitment and follow-up	
	14b	Why the trial ended or was stopped	
Baseline data	15	A table showing baseline demographic and clinical characteristics for each group	See figure 1
Numbers analysed	16	For each group, number of participants (denominator) included in each analysis and whether the analysis was by original assigned groups	
Outcomes and estimation	17a	For each primary and secondary outcome, results for each group, and the estimated effect size and its precision (such as 95% confidence interval)	See table 1 in page 13
	17b	For binary outcomes, presentation of both absolute and relative effect sizes is recommended	
Ancillary analyses	18	Results of any other analyses performed, including subgroup analyses and adjusted analyses, distinguishing pre-specified from exploratory	Page 9 & 10
Harms	19	All important harms or unintended effects in each group (for specific guidance see CONSORT for harms)	
**Discussion**			
Limitations	20	Trial limitations, addressing sources of potential bias, imprecision, and, if relevant, multiplicity of analyses	Page 3
Generalisability	21	Generalisability (external validity, applicability) of the trial findings	Page 3
Interpretation	22	Interpretation consistent with results, balancing benefits and harms, and considering other relevant evidence	
**Other information**			
Registration	23	Registration number and name of trial registry	Page 2
Protocol	24	Where the full trial protocol can be accessed, if available	NA
Funding	25	Sources of funding and other support (such as supply of drugs), role of funders	Page 11

*We strongly recommend reading this statement in conjunction with the CONSORT 2010 Explanation and Elaboration for important clarifications on all the items. If relevant, we also recommend reading CONSORT extensions for cluster randomised trials, non-inferiority and equivalence trials, non-pharmacological treatments, herbal interventions, and pragmatic trials. Additional extensions are forthcoming: for those and for up to date references relevant to this checklist, see www.consort-statement.org.

For peer review only - http://bmjopen.bmj.com/site/about/guidelines.xhtml

For peer review only
